# Immature O-glycans recognized by the macrophage glycoreceptor CLEC10A (MGL) are induced by 4-hydroxy-tamoxifen, oxidative stress and DNA-damage in breast cancer cells

**DOI:** 10.1186/s12964-019-0420-9

**Published:** 2019-08-27

**Authors:** Anna-Katharina Kurze, Sophia Buhs, Dennis Eggert, Leticia Oliveira-Ferrer, Volkmar Müller, Axel Niendorf, Christoph Wagener, Peter Nollau

**Affiliations:** 1 Research Institute Children’s Cancer Center and Department of Pediatric Hematology and Oncology, University Medical Center Hamburg-Eppendorf, Martinistr. 52, 20251 Hamburg, Germany; 2 Department of Otorhinolaryngology, University Medical Center Hamburg-Eppendorf, Martinistrasse 52, 20246 Hamburg, Germany; 3 Department of Gynecology, University Medical Center Hamburg-Eppendorf, Martinistr. 52, 20251 Hamburg, Germany; 4 MVZ Prof. Dr. med. A. Niendorf Pathologie Hamburg-West GmbH, Institut für Histologie, Zytologie und molekulare Diagnostik, Lornsenstraße 4, 22767 Hamburg, Germany

**Keywords:** Breast cancer, Hormone dependence, O-glycosylation, Tn-antigen

## Abstract

**Background:**

Ligands of the C-type lectin CLEC10A such as Tn and sialyl-Tn representing early intermediates of O-glycosylation are hallmarks of many human malignancies. A variety of regulatory mechanisms underlying their expression are being discussed.

**Methods:**

CLEC10A ligands were detected in various tissues and cells using the recombinant glycan-binding domain of CLEC10A. In normal breast and endometrium, presence of ligands was correlated to the female cycle. Estrogen- and stress dependent induction of CLEC10A ligands was analyzed in MCF7 and T47D cells exposed to 4-hydroxy-tamoxifen (Tam), zeocin and hydrogen peroxide. The expression and localization of CLEC10A ligands was analyzed by Western blot and immunofluorescence. In breast cancer patients CLEC10A ligand expression and survival was correlated by Kaplan-Meyer analysis.

**Result:**

We observed binding of CLEC10A in normal endometrial and breast tissues during the late phase of the female hormonal cycle suggesting a suppressive effect of female sex hormones on CLEC10A ligand expression. Accordingly, CLEC10A ligands were induced in MCF7- and T47D breast cancer cells after Tam treatment and accumulated on the cell surface and in the endosomal/lysosomal compartment. Phagocytosis experiments indicate that macrophages preferentially internalize CLEC10A ligands coated beads and Tam treated MCF7 cells. CLEC10A ligands were also expressed after the addition of zeocin and hydrogen-peroxide. Each substance induced the production of ROS indicating reactive oxygen species as a unifying mechanism of CLEC10A ligand induction. Mechanistically, increased expression of GalNAc-transferase 6 (GalNT6) and translocation of GalNT2 and GalNT6 from cis- towards trans-Golgi compartment was observed, while protein levels of COSMC and T-synthase remained unaffected. In breast cancer patients, positivity for CLEC10A staining in tumor tissues was associated with improved outcome and survival.

**Conclusion:**

CLEC10A ligands are inducible by hormone depletion, 4-hydroxy-tamoxifen and agents inducing DNA damage and oxidative stress. Our results indicate that CLEC10A acts as a receptor for damaged and dead cells and may play an important role in the uptake of cell debris by macrophages and dendritic cells.

**Graphical Abstract:**

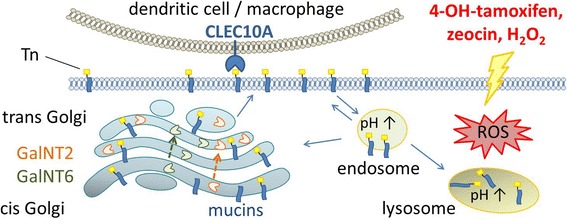

**Electronic supplementary material:**

The online version of this article (10.1186/s12964-019-0420-9) contains supplementary material, which is available to authorized users.

## Background

Breast cancer is the most frequent cancer among women worldwide [1]. Long-life exposure to estrogen is a major risk factor for breast cancer. Up to 80% of breast cancers express the estrogen receptor (ER) [2]. In ER-positive early-stage breast cancer, adjuvant endocrine therapy with ER receptor modulators such as tamoxifen or aromatase inhibitors substantially reduce the risk of loco-regional and distance recurrence [3]. Malignant transformation is accompanied by profound alterations in glycosylation [4, 5]. Glycans are biomolecules of extreme structural diversity exerting a great variety of biological functions like cell-cell and cell-matrix interactions, inflammation and signaling. Glycans protect membranes against proteolytic and glycolytic damage, assist protein folding and alter protein conformation. While N-linked glycan moieties tend to be hyper-sialylated in tumors, O-linked glycans often are truncated resulting in immature glycan structures such as the Tn-, STn- or T-antigens [6–8]. The Tn-antigen is defined as a GalNAc residue bound to serine or threonine by an α-glycosidic linkage [9]. The Tn-structure represents the initial step of O-glycan synthesis involving at least 20 different GalNAc-transferases (GalNTs) and may serve as an acceptor for the sialyltransferase ST6GalNAc-I, yielding sialyl-Tn (STn). Alternatively, T-synthase attaches galactose to GalNAc yielding the T-antigen. COSMC is a chaperone responsible for proper folding of T-synthase. Knockout or knockdown of COSMC or T-synthase, respectively, led to the accumulation of Tn-antigens on the cell surface [10]. Since COSMC- and T-synthase mutations are a rare event in breast cancer, other mechanisms likely contribute to the increased expression of truncated O-glycans. One potential mechanism involve a shift in the localization of glycosyltransferases in organelle compartments after stimulation with growth factors like EGF or PDGF leading to altered glycosylation [11, 12]. Also, elevated pH in the Golgi apparatus appears to be linked to T-antigen expression in cancer cells by affecting the distribution of Golgi-localized glycosyltransferases [13].

Lectins such as *Helix pomatia* agglutinin (HPA) and monoclonal antibodies have been used to correlate the presence of Tn and STn structures on breast cancer tissues with patients’ prognosis. It has been suggested that the expression of Tn and/or STn structures on tumor cells are accompanied by an increased rate of local recurrences and distant metastases [6]. However, frequencies of Tn−/STn- detection and correlation with patients’ outcome vary considerably between studies, which may be explained by the variable specificities of antibodies and lectins applied and the compositions of the patient cohorts [14]. As an alternative strategy for detection of these glycans in human tumors, we used the physiologically expressed glycoreceptor CLEC10A, a member of the family of C-type lectins. CLEC10A expressed by dendritic cells (DCs) and macrophages, preferentially binds terminal GalNAc structures such as the Tn- and STn-antigens [15–20]. Upon binding and internalization of pathogens or glycosylated self-proteins such as MUC1, DCs and macrophages modulate the activity of T-cells [21–23]. In the present study, we investigated CLEC10A ligands of normal tissue and breast cancer cells in dependence of estrogen-depletion and 4-hydroxy-tamoxifen treatment. Since tamoxifen has been reported to induce oxidative stress and DNA damage, we additionally analyzed the effects of hydrogen peroxide and zeocin on CLEC10A ligand synthesis [24, 25]. Our data suggest a link between the production of reactive oxygen species as a response to different cell damaging agents and an increase of CLEC10A ligands on the cell surface. Thus, CLEC10A ligands may serve as glycan danger structures, which act as “eat-me” signals on damaged cells [26, 27].

## Methods

### Cells

HEK293T cells expressing recombinant CLEC10A and the breast cancer cell lines MCF7, T47D and MDA-MB-231 were purchased form ATCC and maintained in Dulbecco’s Modified Eagle’s Medium (DMEM) containing 10% fetal calf serum (FCS) and 100 U/ml penicillin and 100 μg/ml streptomycin. The identity of cell lines was confirmed by STR analysis. For hormone depletion, cells were cultured in DMEM without phenol red supplemented with 10% heat-inactivated and charcoal-stripped fetal bovine serum (Gibco) for four days before 10 nM 17β-estradiol and / or progesterone (both from Sigma-Aldrich) were added for 24 h. For drug treatment, the active metabolite of tamoxifen, 4-hydroxy-tamoxifen (Tam; final concentration 2 or 4 μM; Sigma-Aldrich), zeocin (250 μg/ml; Thermo Fisher Scientific) and hydrogen peroxide (30 μM; Merck) were added to breast cancer cell lines for for 48 h to 72 h. After 24 h media was renewed.

For macrophage differentiation, peripheral blood mononuclear cells (PBMC) were isolated from buffy coats by gradient centrifugation. After washing with PBS containing 2 mM EDTA, 1.5 × 10^6^ PBMC per cm^2^ were seeded into multiwell plates and incubated in RPMI without serum for 2 h at 37 °C to allow monocytes to attach to the plastic surface. Subsequently, cells were washed and cultivated in RPMI with 10% heat inactivated FCS, 100 U/ml penicillin, 100 μg/ml streptomycin and 25 ng/ml M-CSF (PeproTech) for 7 days. In vitro differentiation of macrophages was confirmed by FACS determining the surface expression of CD16 and CLEC10A.

### Tissue and patient samples

Paraffin-embedded tissue microarrays (TMAs) of formalin-fixed human endometrium and various normal tissues were purchased from US Biomax (Rockville, MD). The normal tissue TMA was composed of 90 spots (diameter 1 μm) representing 45 normal human tissues. Histopathological characteristics of the human breast cancer samples provided by the MVZ Prof. Dr. med. A. Niendorf, Pathologie Hamburg-West GmbH are summarized in Additional file 1: Table S1. Normal breast tissues and corresponding serum samples were obtained from the Department of Gynecology at University Medical Center Hamburg-Eppendorf. Estrogen, progesterone, luteinizing hormone (LH) and follicle-stimulating hormone (FSH) were determined in corresponding serum samples of normal human breast tissues by Labor Lademannbogen (Hamburg, Germany). Survival analyses and Cox regression analysis were performed applying the Sigma Plot software package 11 (Systat Software Inc).

### Cloning and expression of soluble recombinant CLEC10A

The extracellular part of human C-type glycoreceptor CLEC10A was cloned and expressed as described before [18]. In brief, the extracellular part of CLEC10A was amplified by PCR from normal human lymphocyte cDNA. For secretion and detection, IgG kappa-leader and a c-myc-tag were fused to the N-terminus of CLEC10A and cloned into the pcDNA3.1 expression vector. For expression, HEK293T-cells were transiently transfected using Lipofectamine 2000 (Thermo Fisher Scientific) and recombinant CLEC10A was harvested from the supernatant.

### Western blotting and antibodies

Western blots were performed as described before using the antibodies indicated in Additional file 1: Table S2 [18]. For the detection of CLEC10A ligands, recombinant CLEC10A was complexed using biotinylated anti c-myc antibody and Streptavidin-HRP (Thermo Scientific), the complex was diluted 1:5 in TSM buffer (10 mM Tris-HCl, pH 7.4, 150 mM NaCl, 2 mM MgCl_2_, and 1 mM CaCl_2_, 0.1% Tween 20) and was added to the blocked membranes. After washing, signals were obtained by chemiluminescence as described previously.

### Cell surface biotinylation

Breast cancer cells were washed with ice-cold PBS and incubated with PBS containing 0.5 mg/ml non-permeable EZ-Link™ Sulfo-NHS-SS-Biotin (Thermo Scientific) at 4 °C on ice for one hour. Cells were washed and harvested by scraping in PBS containing 0.2% Triton-X100 and protease inhibitors (Proteinase Inhibitor Cocktail, Pierce). For pulldown of biotinylated proteins, 200 μg total protein extract were incubated with 20 μl high capacity streptavidin agarose (Pierce) overnight at 4 °C. Beads were washed, eluted by incubation in Laemmli buffer containing DTT at 95 °C for 5 min and analyzed by Western blot.

### Acridine Orange staining

Acridine Orange was added to the growth medium to a final concentration of 2 μg/ml and cells were incubated for 20 min at 37 °C, washed twice with PBS and directly analyzed by fluorescence microscopy.

### ROS assay

MCF7- and T47D cells were seeded in 12 well plates for 48 h. Immediately prior use a fresh 10 mM stock solution of H2DCFDA (Thermo Fisher Scientific) dissolved in dimethyl sulfoxide was prepared. For the measurement of short-term ROS production, cells were washed with PBS and stained with 5 μM H2DCFDA in serum free media for 30 min at 37 °C protected from light. Afterwards cells were washed with PBS and incubated for 2.5 h at 37 °C in the dark with media containing ethanol [E],Tam (4 μM) [T], zeocin (250 μg/ml) [Z] or hydrogen peroxide (30 μM) [H], respectively. For ROS measurement after 48 h of treatment, which is the time period used for most of the experiments, cells were stained with 5 μm H2DCFDA for 30 min after treatment with ethanol, Tam zeocin or hydrogen peroxide.

After staining, the cells were processed for FACS analysis. Cells were washed with PBS, removed from the plate using trypsin and transferred to FACS tubes. After washing with PBS, FITC intensities of the cells were measured in quadruplicates by flow cytometry. Signals of unstained cells served as background controls and were subtracted from signals from stained cells. Results of stained, treated cells (E, T, Z, H) were normalized to stained, non-treated cells (N). The averages and standard deviations were calculated and Student’s t-test was performed for determination of significance.

### Phagocytosis assay

10 μl Neutravidin-coated microspheres (1 μm) (Molecular probes/Invitrogen) were washed three times with PBS, 0.1% BSA. For coupling, beads were diluted in 700 μl PBS, 0.1% BSA and 3 μg biotinylated Tn antigen or biotinylated spacer control (Lectinity) were added and incubated at 4 °C for 16 h. Beads were washed two times with PBS, 0.1% BSA and resuspended in 1 ml PBS, 0.1% BSA. Macrophages were incubated with 100 μl bead solution for 2 h at 37 °C. As a control for calcium-dependent uptake, analyses were additionally performed in the presence of EDTA (5 mM). The number of fluorescently labeled macrophages was determined by FACS.

To investigate phagocytosis of cells, MCF7 cells were labeled by 5(6)-Carboxyfluorescein diacetate N-succinimidyl ester (CFSE, Sigma Aldrich) and incubated with macrophages. Cell numbers of macrophages, which internalized MCF7 cells, were detected by FACS as CFSE- and CLEC10A-positive cells (Additional file 1: Table S2). In brief, 4-hydroxy-tamoxifen or solvent treated MCF7 cells were washed with PBS and incubated with 5 μM CFSE in PBS for 20 min at 37 °C. Cells were washed, detached by trypsin and resuspended in DMEM with 10% FCS. To investigate the impact of CLEC10A ligands on the clearance of dead cells, aliquots of labelled cells were treated by three freeze and thaw cycles. Target cells were incubated with macrophages for 2 h at 37 °C. Non-ingested target cells were removed by washing and macrophages were stained with an APC-labeled anti–CLEC10A after blocking Fc-receptors (Human TruStain FcX™; Biolegend). An APC-labeled mouse IgG2a isotype antibody (Biolegend) was used as control.

All FACS analyses were performed on a BD FACSCantoTM flow cytometer and data were analyzed by the FACSDiva software (BD Biosciences) and Flowing Software 2.5.1 (Turku University, Finland). The gating strategy is given in detail in Additional file 1: Figure S2B.

### Immunofluorescence

Cells were fixed with 3% formaldehyde (FA) or methanol (M) depending on the antibodies applied (Additional file 1: Table S2). FA-fixed cells were permeabilized with 0.1% Triton X-100 in PBS and blocked with 2% BSA in PBS. Cells were incubated with primary antibodies diluted in blocking solution. After washing fluorescently labeled secondary antibodies were added. After additional washing Nuclei were stained by DAPI. Slides were mounted with Prolong GOLD antifade reagent (Life Technologies). For staining of glycans by PNA (Vector Labs) or CLEC10A, incubation was performed in TSM buffer (10 mM Tris pH 7.4, 150 NaCl, 1 mM CaCl_2_, 2 mM MgCl_2_). PNA was preincubated with Strep-Cy3 and recombinant CLEC10A was preincubated with 3 μg/ml monoclonal anti c-myc-antibody. After incubation and washing with TSM goat anti-mouse Alexa 488 secondary antibody for detection of bound CLEC10A was applied. Images were acquired either using a NikonTi2 microscope equipped with a DS-Qi2 camera and Plan Apo λ 60x and 100x objectives or a Leica DMIL epifluorescence microscope with the DFC420C camera and HCX PL Fluotar 63x and 100x objectives. Mander’s coefficient for co-localization was determined from 10 images applying the ImageJ plugin JACOP; levels of significance were calculated by the Student’s t-test.

### Histochemical staining using recombinant CLEC10A

Tissue sections were stained with recombinant CLEC10A as described previously [28]. Briefly, sections were deparaffinized and antigen retrieval was achieved by boiling in 0.1 M sodium citrate buffer (pH 5.0). Slides were blocked by 3% hydrogen peroxide and with TSM buffer in the presence of 0.2% BSA, 10% fetal calf serum and 0.3% Triton X-100. Tissue sections were incubated with complexed CLEC10A consisting of myc-tagged CLEC10A, streptavidin-horseradish peroxidase (HRP) conjugate (Thermo Fisher Scientific), and the biotinylated anti cmyc antibody 9E10 (Santa Cruz Biotechnology). After washing (3× for 5 min each) in TSM buffer, staining was performed with 3, 3′-diaminobenzidine chromogen solution (DAB; Dako) Nuclei were counterstained by hematoxylin. Stained tissue sections were coverslipped applying Glycergel Mounting Medium (Dako). Images were acquired using an Olympus BX43 microscope.

### Pull-down of CLEC10A ligands

Recombinant CLEC10A carrying a myc-tag was incubated with anti c-myc agarose beads overnight at 4 °C. 300 μg of total protein lysate isolated from two CLEC10A-positive tumors were incubated with 50 μl of CLEC10A coupled c-myc-agarose beads in 500 μl binding buffer (TBS, 0.1% Triton-X100, 1 mM CaCl_2_, 2 mM MgCl_2_). Beads were washed with binding buffer before glycoproteins were eluted with TBS containing 10 mM EDTA (pH 7.4). For mass spectrometry, eluates were separated by SDS PAGE gel electrophoresis followed by silver staining (Silver Staining Kit, Proteome Factory). Gel lanes were cut, divided into three slices and proteins bound by CLEC10A were identified by mass spectrometry (Proteome Factory).

## Results

### Hormone-dependent expression of CLEC10A positive glycan structures in normal human tissues

**Fig. 1:**
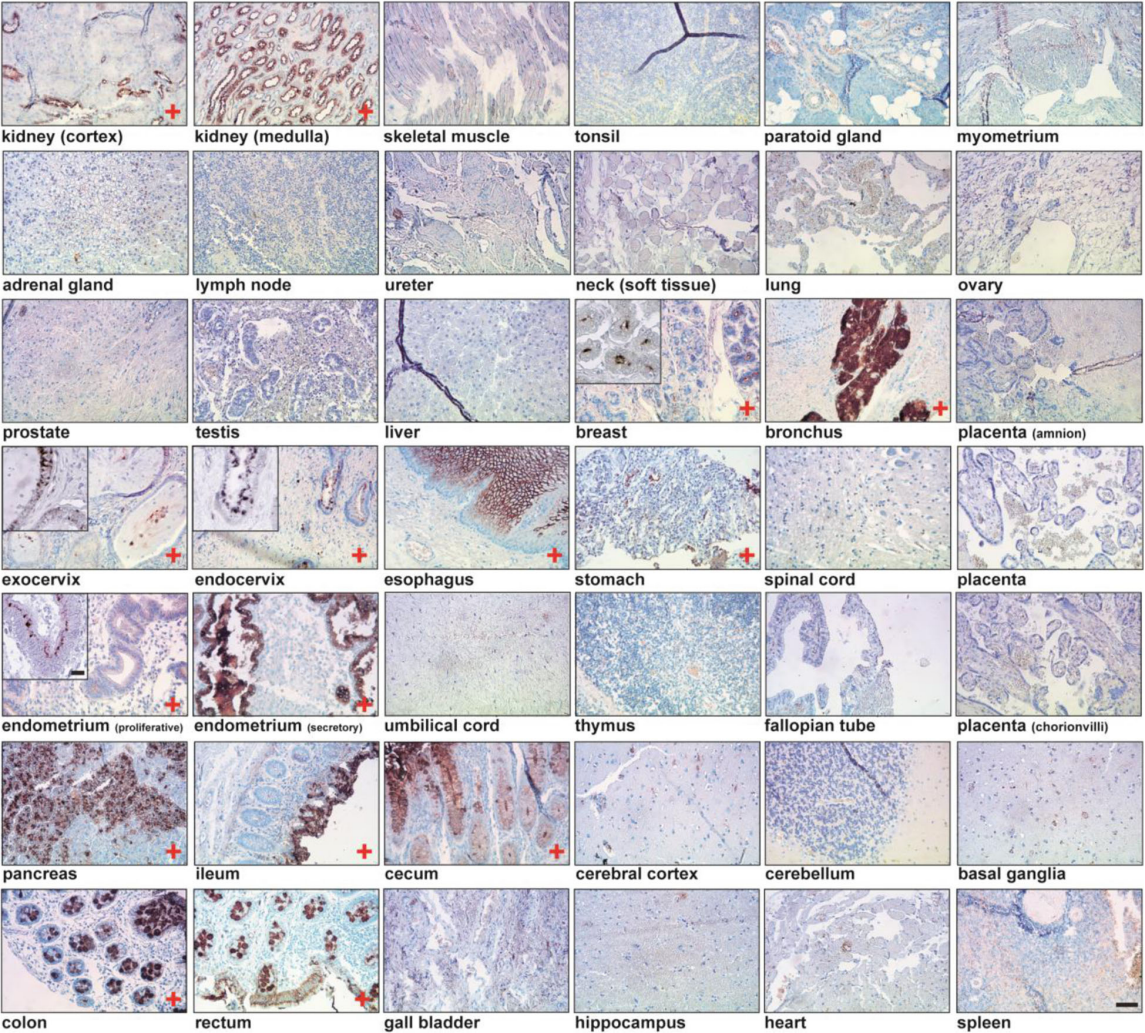
CLEC10A staining of various normal human tissues arranged on a tissue microarray. Protein domain histochemistry was performed after complexing of recombinant, myc-tagged CLEC10A with a biotinylated anti-myc antibody conjugated to streptavidin-horseradish peroxidase. 3,3′-diamino-benzidine (DAB) was used as chromogenic substrate and tissues were counterstained with hematoxylin. Tissues stained positive for CLEC10A are marked with “+“. Scale Bar: 100 μm. Inserts with higher magnification of representative tissue areas are given for breast, cervical and endometrium tissues (scale bar: 10 μm)

**Fig. 2:**
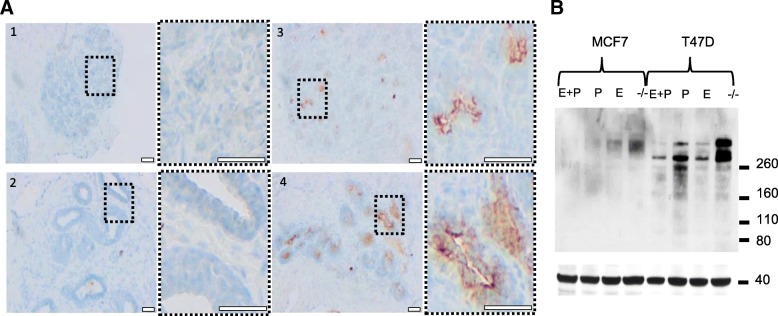
Expression of CLEC10A positive glycan structures in normal human breast tissue and breast cancer cell lines is dependent on female sex hormones. **a** Applying protein domain histochemistry, cryosections of normal human breast tissue obtained during the proliferative phase (#1 + #2) or luteal phase (#3 + #4) of the female hormonal cycle were stained with CLEC10A as described in Fig. 1. Scale bar: 100 μm. Magnifications of the marked areas are given to the right of each overview; scale bar: 100 μm. To determine the phase of the female hormonal cycle, levels of the female sex hormones FSH, LH, 17β-estradiol (E) and progesterone (P) in corresponding serum samples were determined as follows: #1 (age 34 y) FSH: 7.5 IU/ml, LH: 3.9 U/l, E: 57 pg/ml, P: 0.1 ng/ml; #2 (age 43 y) FSH: 6.2 IU/ml, LH: 7.4 U/l, E: 142 pg/ml, P: 0.3 ng/ml; #3 (age 43 y) FSH: 4.1 IU/ml, LH: 5.0 U/l, E: 57 pg/ml, P: 5.3 ng/ml; #4 (age 44 y) FSH: 4.2 IU/ml, LH: 1.2 U/l, E: 116 pg/ml, P: 12.2 ng/ml. Samples were assigned to the luteal phase when serum levels of progesterone were *P* > 2 ng/ml, FSH < 8 IU/ml and LH in the range between 1 and 11 U/l. **b** Far Western Blot analysis of total protein extracts (20 μg/lane) from hormone depleted (−/−) MCF7 and T47D cells using recombinant CLEC10A as probe. Cells were cultivated in hormone-depleted medium 4 days before estrogen (E) or progesterone (P) or a combination of both hormones (E + P) was added for 24 h. β-actin served as loading control (lower panel)

Based on protein domain histochemistry, we previously investigated the binding of the glycoreceptor CLEC10A to breast cancer tissue. Besides frequent staining of carcinoma cells, CLEC10A binding to lobular epithelial cells of normal breast tissues was observed with variable frequency, preferentially localized at the luminal part of the acinus [28]. Here, we extended our investigations on CLEC10A binding to different normal human tissues arranged on a tissue microarray (Fig. 1). Binding of CLEC10A was detected in glandular epithelial cells of the breast, the gastrointestinal tract as well as of the bronchus, kidney and cervix; in general, CLEC10A positivity was confined preferentially to the apical portions of the cells. Surprisingly, normal, glandular epithelial cells of the endometrium obtained during the secretory phase of the female hormonal cycle stained strongly positive for CLEC10A, while endometrium of the proliferative phase was largely negative or only weakly positive. This finding and the previously observed, variable positivity for CLEC10A staining in normal breast tissues prompted us to speculate that the expression of CLEC10A-ligands may be regulated in normal breast and endometrial tissues by female sex hormones. An extension of our studies on human endometrial tissues obtained during different phases of the female cycle confirmed that glycan structures recognized by CLEC10A are preferentially expressed during the final, secretory phase of the menstrual cycle (Additional file 1: Figure S1). To further study the hormone dependency of CLEC10A ligand expression in normal human breast tissues, we collected normal mammary tissues from breast reduction surgery of premenopausal women. In parallel and to assign the samples to the different phases of the female hormonal cycle, levels of female sex hormones were determined in the corresponding serum samples. In accordance with our findings in the endometrium, pronounced CLEC10A staining of normal lobular breast epithelial cells was observed during the luteal but not during the follicular phase indicating that CLEC10A positive glycan structures are expressed during the late phase of the female hormonal cycle presumably when levels of estrogen and progesterone are declining (Fig. 2a). To substantiate our hypothesis that CLEC10A ligand expression is regulated by female sexual hormones, we studied the induction of glycan structures recognized by CLEC10A in the two estrogen and progesterone receptor (ER/PR) positive breast cancer cell lines T47D and MCF7 after withdrawal and re-addition of estrogen and progesterone, respectively (Fig. 2b). Cells were cultivated in hormone-depleted medium for 4 days followed by the re-addition of estrogen, progesterone or a combination of both hormones for 24 h. Western blot analysis of T47D and MCF7 cells revealed that hormone depletion resulted in strong induction of CLEC10A-positive glycan structures. This effect was reversed by the re-addition of estrogen or progesterone, respectively. We conclude that the expression of glycan structures recognized by CLEC10A in normal breast and endometrium as well as breast cancer cell lines depends on female sex hormones. To our knowledge, this finding has not been reported so far.

### CLEC10A positive glycan structures are induced by 4-hydroxy-tamoxifen and associated with alterations in glycan synthesis and glycoprotein processing

**Fig. 3:**
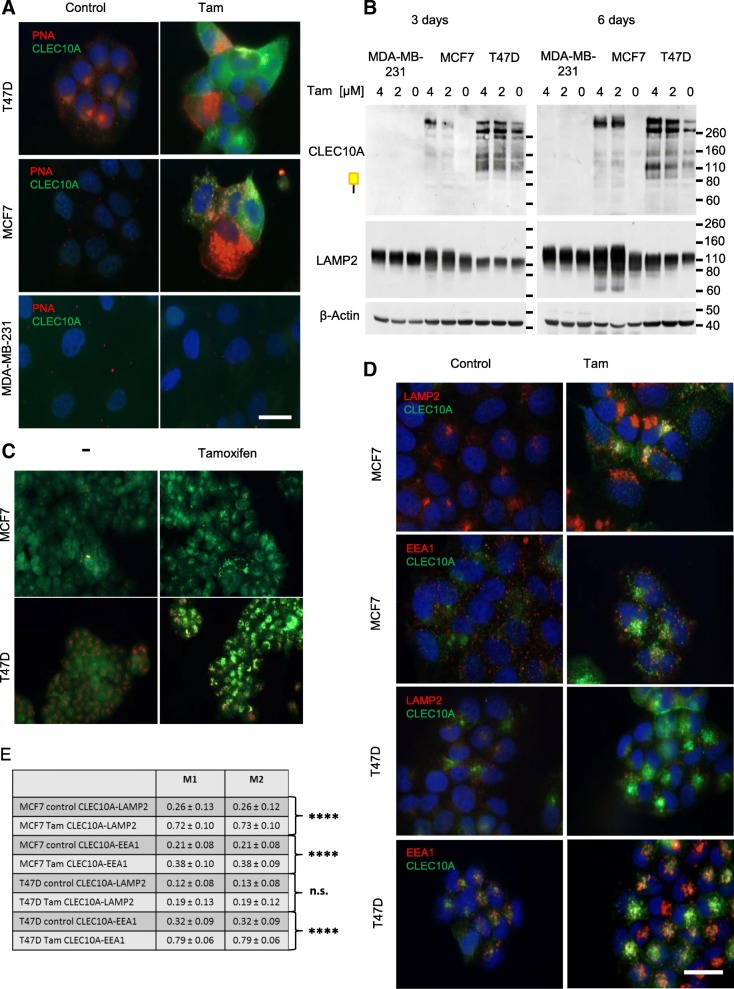
Impaired glycan synthesis and endosomal/lysosomal processing results in the accumulation of CLEC10A positive glycan structures after Tam treatment of breast cancer cell lines. **a** Immunofluorescence of MCF7, T47D and MDA-MB-231 cells treated by Tam (4 μM) or ethanol (control) for 72 h. Cells were fixed and stained with recombinant CLEC10A using an anti c-myc antibody and a secondary anti-mouse antibody labeled with Alexa 488 (green) or PNA labeled with Streptavidin-Cy3 (red); nuclei (blue) were counterstained by DAPI. Scale bar: 20 μm. **b** Western blot analysis of whole cellular extracts of MCF7, T47D and MDA-MB-231 treated with 2 μM and 4 μM Tam for 3 and 6 days, respectively. Lysosomal membrane protein LAMP2 was detected using a monoclonal antibody. β-actin served as control for equal loading. **c** MCF7 and T47D cells stained with acridine orange after treatment with 4 μM Tamoxifen (Tam) for 48 h; ethanol treated cells served as control (−). Upon exposure to Tam intracellular vesicles increase in size and stain green or yellow suggesting lysosomal swelling and an increase in lysosomal pH; scale bar: 20 μm. **d** Co-localization of CLEC10A ligands (Alexa 488, green) with LAMP2-positive lysosomes or EEA1-positive early endosomes (Alexa 555, red). MCF7 and T47D cells were treated with 4 μM Tamoxifen or ethanol (control) for 48 h, respectively. Yellow indicates co-localization in merged images. Nuclei (blue) were visualized by DAPI staining. Scale bar: 20 μm. **e** Mander’s coefficient of the co-localization of CLEC10A with early endosomes and lysosomes. For statistical anaylsis, co-localization was determined in 10 different areas of the image applying JACOB. Averages and standard deviations are given. *P*-values were calculated by Student’s t-test. **** *P* < 0.0001

To further study the mechanism of hormone dependent induction of CLEC10A ligands, we treated MCF7 and T47D breast cancer cells as well as the ER−/PR-negative breast cancer cell line MDA-MB-231 with the estrogen receptor modulator 4-hydroxy-tamoxifen (Tam). As shown by protein domain histochemistry and in line with our estrogen/progesterone depletion experiments, Tam induced a strong increase of CLEC10A binding in both ER/PR-positive cell lines while the hormone independent MDA-MB-231 cell line stained negative (Fig. 3a). Positive staining for CLEC10A occurred at the cell surface and in large intracellular vesicles in both cell lines possibly resembling enlarged endosomes, lysosomes or autophagosomes, respectively. Interestingly, Tam treated MCF7 and T47D cells stained also positive for the plant lectin PNA (Peanut Agglutinin). PNA recognizes terminal galactose structures such as the T-antigen commonly present in early intermediates during O-glycosylation. The accumulation of immature glycan structures suggest that glycan elongation may be impaired by Tam, leading to the accumulation of early intermediates of glycan synthesis such as the Tn- and T-antigen detected by CLEC10A and PNA, respectively.

To confirm our findings on the induction of glycan structures by Tam, we analyzed the time- and dose-dependent expression of CLEC10A positive glycan structures by Western blot (Fig. 3b). Treatment of MCF7 and T47D cells for 3 days and 6 days, respectively, by Tam at concentrations of 2 μM and 4 μM resulted in the induction of CLEC10A positive glycan structures increasing over time of treatment; in contrast, no induction was observed in hormone independent MDA-MB-231 cells. Western blot analysis of the lysosomal membrane protein LAMP2 (lysosome-associated membrane protein 2), regulating lysosomal stability and autophagy, revealed increased protein levels over time in MCF7 and T47D cells. (Fig. 3b). Since protein levels of LAMP2 increase also in lysosomal storage diseases such as neuronal ceroid lipofuscinosis or after treatment with lysosomotropic drugs [29], these results suggest that the endosomal/lysosomal pathway is also affected by Tam. In addition, acridine orange staining was used to analyze vesicular structures and the intracellular pH of living cells after Tam treatment (Fig. 3c) [30]. The color detected by immunofluorescence serves as an indicator of the intracellular pH ranging from green (neutral) over weakly acidic (yellow) to acidic (red). Staining of Tam treated MCF7 and T47D cells by acridine orange showed an accumulation and enlargement of vesicular structures accompanied by intracellular alkalization. To gain further insights into the vesicular accumulation of CLEC10A positive glycan structures, we investigated the co-localization of CLEC10A positive glycoproteins with LAMP2 and the early endosomal antigen 1 (EEA1) by immunofluorescence (Fig. 3d+e). In accordance with our Western blot data, CLEC10A ligands significantly accumulated in LAMP2 positive lysosomes in MCF7 cells whileco-localization between CLEC10A and EEA1 in endosomes was observed to a lesser extent. In contrast, LAMP2 staining was diffuse and relatively weak in T47D cells and no significant co-localization of CLEC10A-positive glycoproteins and LAMP2 was observed. Instead, CLEC10A positive glycan structures significantly co-localized with EEA1 in T47D cells indicating accumulation of CLEC10A ligands in endosomes, which may be caused by a defect in lysosome formation or by a perturbed transport of CLEC10A ligands from endosomes to lysosomes. Taken together, our findings indicate that inhibition of the endosomal/lysosomal compartment is part of the mechanism leading to the accumulation of CLEC10A positive glycan structures after estrogen receptor blockade by Tam.

**Fig. 4:**
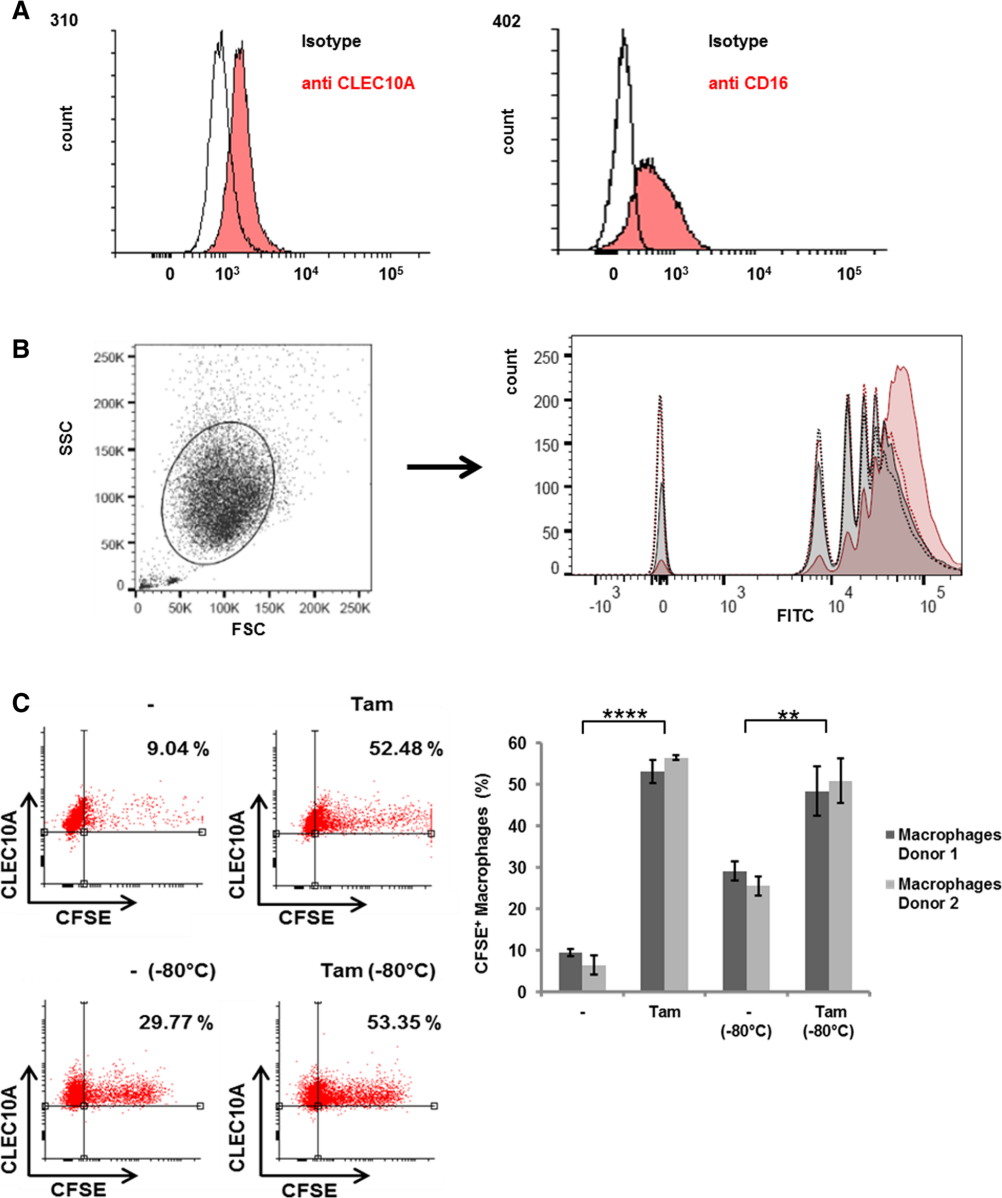
Increased phagocytosis of CLEC10A ligands by macrophages. **a** Surface expression of CLEC10A and CD16 on macrophages derived from human PBMC of healthy donors after differentiation by M-CSF. Subsequent blocking of Fc-receptors, macrophages were stained by using an APC labeled anti-CLEC10A antibody and a PerCP-Cy5 labeled anti CD16-antibody, respectively (red filled histograms). Fluorescence intensity was compared to cells stained with the corresponding isotype controls (non-filled histogram). **b** Increased uptake of fluorospheres carrying Tn-antigen (red, filled histogram) in macrophages compared to control beads carrying the spacer (grey, filled histogram). As a control for calcium-dependent internalization, the uptake of Tn- and control beads was investigated in the presence of EDTA (red dotted line and grey dotted line). The different peaks are due to the uptake of distinct numbers of particles per macrophage. **c** Macrophages from two independent donors were incubated with CFSE-labelled MCF7 cells treated with 4 μM Tamoxifen (Tam) for 48 h or with cells treated with solvent control (−). To analyze engulfment of dead cells by macrophages, aliquots of labelled cells were treated by freeze and thaw cycles (− 80 °C). The amount of CFSE and CLEC10A double positive cells were measured by flow cytometry. Dot plots of four representative measurements are given. Error bars depict the standard deviation of three technical replicates. *p* values: donor 1 – vs. Tam = 0.000014; donor 1 – (− 80 °C) vs. Tam (− 80 °C) = 0,0061; donor 2 – vs. Tam = 0.0000035; donor 2 – (− 80 °C) vs. Tam (− 80 °C) = 0.0018

To investigate the effect of CLEC10A ligands on phagocytosis by macrophages, we incubated CLEC10A positive macrophages with fluorescent beads coated by the Tn antigen attached to a spacer and biotin (Fig. 4). As control, fluorescent beads coated only with the biotinylated spacer moiety were used. In addition, to control for calcium-dependent uptake we performed phagocytosis experiments in the presence of EDTA. Fluorescence intensity was determined by flow cytometry and demonstrated an increased, calcium-dependent uptake of Tn-beads compared to controls (Fig.4a). Since macrophages engulf cells and cell debris, phagocytosis of Tam-treated MCF7 cells was examined in comparison to untreated cells (Fig. 4b). For this purpose, MCF7 cells labeled by CFSE (Additional file 1: Figure S2A) were incubated with macrophages generated by in vitro differentiation of peripheral blood monocytes of healthy donors (Fig. 4b). After phagocytosis the number of macrophages positive for CFSE and CLEC10A was measured by flow cytometry (Fig. 4b. and Additional file 1: Figure S2B). Macrophages internalized Tam treated cells preferentially as compared to control cells (Fig. 4 a + b). To exclude that this result is caused by uptake of dead cells increased in the Tam treated sample, the experiment was performed in parallel with MCF7 cells destroyed by freeze and thaw cycles after CFSE-labeling (Fig. 4c). Comparable to our previous results, uptake of dead MCF7 cells by macrophages was increased after Tamtreatment indicating that CLEC10A ligands are enhancers for the internalization of cells and cell debris.

### CLEC10A positive glycan structures accumulate on the cell surface, are inducible by zeocin and hydrogen peroxide, and are associated with altered expression and localization of GalNTs

**Fig. 5:**
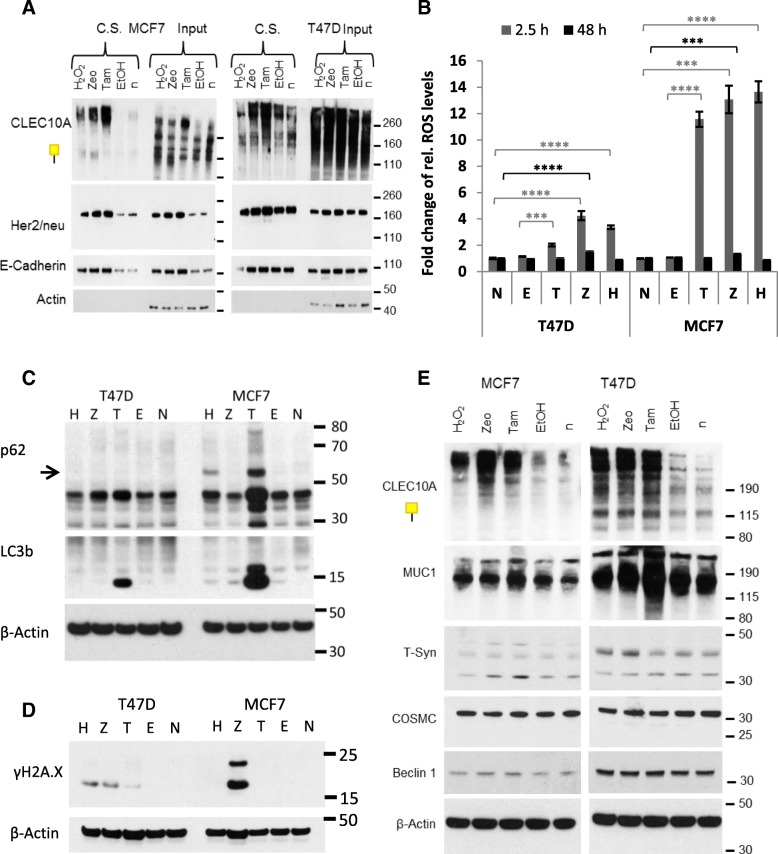
Cell surface localization of CLEC10A ligands and analysis of the expression of different components of the O-glycosylation machinery (**a**) Cell surface proteins of MCF7 and T47D cells were biotinylated with non-cell-permeable sulfo-NHS-SS-biotin after 48 h treatment by Tam (4 μM), Zeocin (Zeo; 250 μg/ml) and hydrogen peroxide (H_2_O_2_; 30 μM), respectively. Non-treated cells (n) and cells treated by ethanol (EtOH) served as controls. After cell lysis, biotinylated surface proteins were precipitated by streptavidin agarose. Western blot analyses were performed with CLEC10A and monoclonal antibodies directed against Her2/neu (ERBB2), and E-cadherin; C.S.: cell surface. **b** ROS measurements in MCF7 and T47D cells after 2.5 h and 48 h, respectively. FITC intensities of the cells were measured in quadruplicates by flow cytometry. Signals of unstained cells served as background controls and were subtracted from signals from stained cells. Results of stained, treated cells (E, T, Z, H) were normalized to stained, non-treated cells (N). The averages and standard deviations were calculated and Student’s t-test was performed for determination of significance. *** *P* < 0.001, **** *P* < 0.0001. **c** Western blot analysis of proteins LC3b and p62 involved in autophagy in Tam, zeocin and hydrogen peroxide treated cells. Untreated (n) and ethanol (EtOH) treated cells served as controls. MCF7 and T47D cells were treated for 48 h, lysed, and 20 μg of total protein was subjected to SDS-PAGE. β-actin served as loading control. **d** Western Blot analysis of γH2A.X as a marker for DNA damages. β-actin served as loading control. **e** Western blot analysis of levels of MUC1, T-synthase, COSMC and Beclin 1 protein expression in Tam, zeocin and hydrogen peroxide treated MCF7 and T47D cells in comparison to untreated controls (n). Cells were treated as described above. β-actin served as loading control

Given that glycoproteins recognized by CLEC10A are intracellularly accumulating after Tam treatment due to impaired endosomal/lysosomal processing and incomplete glycosylation, we asked whether CLEC10A positive glycan structures are also accumulating on the cell surface. To test for this, MCF7 and T47D cells were treated by Tam, cell surface proteins were biotinylated and enriched by streptavidin subsequent to cell lysis (Fig. 5a). Western blot analysis demonstrated that CLEC10A positive glycan structures are strongly enriched on the cell surface of MCF7 and T47D cells after Tam treatment. To address the question whether CLEC10A positive glycan structures are also induced by cell damaging substances, we tested the effects of phleomycin D1 (zeocin) and hydrogen peroxide in addition to Tam (Fig. 5a). Zeocin is a glycopeptide antibiotic of the bleomycin family causing DNA damage, and hydrogen peroxide causes oxidative stress by oxidation of proteins, membrane lipids and DNA by the peroxide ions [31–33]. Interestingly and comparable to Tam, treatment of MCF7 cells by zeocin or hydrogen peroxide resulted in a strong expression of CLEC10A positive glycan structures on the cell surface. Increased expression of CLEC10A ligands on the cell surface was also observed after treatment of T47D cells by zeocin while hydrogen peroxide had no significant effect. We additionally analyzed the surface expression of the membrane proteins ERBB2 (HER2/neu) and E-cadherin. Strong accumulation of both membrane proteins was observed in MCF7 cells after treatment by Tam, zeocin and hydrogen peroxide, respectively, and to a lesser extent in T47D cells after Tam and zeocin treatment. As demonstrated by acridine orange staining and in line with our previous results, treatment of MCF7 and T47D cells by zeocin resulted in the enlargement of intracellular vesicles; alkalization of acidic organelles was predominantly observed in MCF7 cells suggesting that membrane proteins are broadly accumulating on the cell surface as a consequence of impaired endosomal trafficking and lysosomal degradation (Additional file 1: Figure S3).

To further investigate the mechanisms underlying the increased expression of CLEC10A ligands induced by Tam, zeocin and hydrogen peroxide, respectively, we analyzed the generation of ROS in MCF7- and T47D cells after 2.5 h and 48 h of treatment (Fig. 5b). After 2.5 h each of the substances increased the intracellular ROS levels in both cell lines, being more pronounced in MCF7 cells compared to T47D. With the exception of zeocin treatment, ROS levels decreased to baseline after 48 h.

To test the effects of Tam, zeocin and hydrogen peroxide treatment on cell viability MTT assays were performed. In T47D cells, all substances significantly decreased viability 24 h and 48 h after treatment. In MCF7 cells, Tam had a positive effect on viability after 24 h, but not after 48 h. zeocin and hydrogen peroxide reduced viability after 24 h and 48 h (Additional file 1: Figure S4). In MCF7 cells Tam and hydrogen peroxide strongly inhibited migration, whereas zeocin had no effect. Since the migratory activity of T47D was particularly low, no effect of the different substances on migration was detectable (Additional file 1: Figure S5).

To get further insights into the cellular effects of the different substances we analyzed the protein levels of LC3b, p62 and Beclin1 as markers of autophagy in Western blots. Beclin1 is a marker of initiation and phagophore nucleation, p62 acts as autophagy receptor during cargo sequestration, and LC3 is involved in cargo sequestration, membrane sealing and autophagosome maturation [34]. Tam treatment led to a pronounced increase of p62 levels in MCF7 cells, and to a lesser extent in T47D cells (Fig. 5c). Similarly, Tam lead to increased levels of LC3b in MCF7 cells and, to a lesser degree, in T47D cells, whereas the protein levels of Beclin1 remained unaffected (Fig. 5c+e and Additional file 1: Figure S6A + B), indicating rather the inhibition of autophagosome degradation than enhanced induction of autophagy. In zeocin and hydrogen peroxide treated MCF7 and T47D cells no changes in autophagy were observed. In order to investigate DNA damage, γH2A.X was analyzed by western blot. γH2A.X belongs to the histone protein family and serves as a sensor for DNA double strand breaks. As expected, increased levels of γH2A.X were detected after zeocin treatment in both cell lines. Additionally Tam- and hydrogen peroxide treatment led to an increase of γH2A.X in T47D cells (Fig. 5d Additional file 1: Figure S6B).

To further elucidate alterations in the glycosylation pathway, we investigated the expression of different components of the O-glycosylation machinery by Western blot (Fig. 5e). We did not observe significant changes in the protein levels of MUC1, T-synthase or COSMC after treatment of MCF7 cells by Tam, zeocin and hydrogen peroxide, respectively. Also contrary to the presumption that elongation of glycan chains could be impaired by poor expression of COSMC or T-synthase, protein level of COSMC was not altered in T47D cells and levels of T-synthase were even increased after zeocin and hydrogen peroxide treatment. Interestingly, MUC1 levels were substantially elevated in T47D cells after Tam treatment providing evidence that upregulation of the carrier protein MUC1 may additionally contribute to the accumulation of CLEC10A positive glycan structures in T47D cells. Patterns of RNA levels differed between MCF7 and T47D cells and did not correlate with protein levels, indicating that accumulation of CLEC10A ligands are not simply caused by alterations of the transcription of the respective genes, instead changes in protein stability are likely involved (Additional file 1: Figure S6D).

**Fig. 6:**
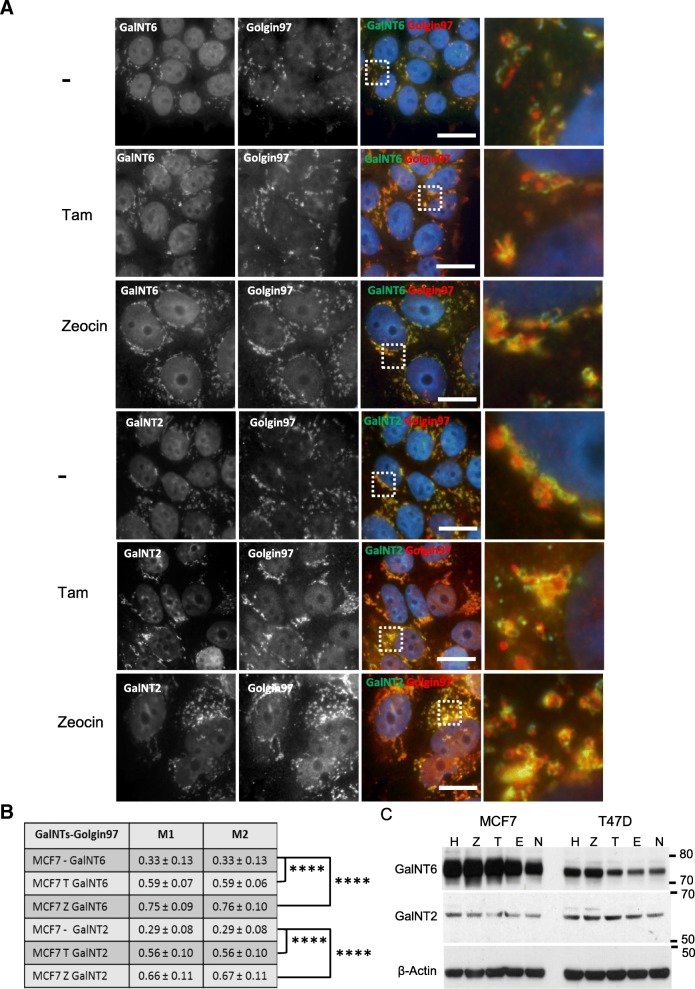
Translocation of GalNT2 and GalNT6 towards the trans Golgi after Tamoxifen and Zeocin treatment. **a** Localization and distribution of GalNT2 and GalNT6 (Alexa 488, green) in the Golgi apparatus of MCF7 cells using the trans-Golgi marker Golgin97 (Alexa 555, red) in comparison to untreated cells. Both cell lines were treated for 48 h by Tam (4 μM) and zeocin (250 μg/ml), respectively. Nuclei colored in blue were stained by DAPI. Scale bar: 20 μm. Magnification of the marked areas are shown. **b** Mander’s coefficient of the co-localization of GalNTs with the trans Golgi protein Golgin97. Averages and standard deviations of 10 co-localization measurements are given. *P-*values were calculated with Student’s t-test. **** *P* < 0.0001. **c** Western blot analysis of levels of GalNT6 and GalNT2 protein expression in Tam, zeocin and hydrogen peroxide treated MCF7 and T47D cells in comparison to untreated controls (n). Cells were treated as described above. β-Actin served as loading control

To further test for alterations in the glycosylation pathway, we analyzed GalNT2 and GalNT6 from the family of N-acetylgalactosaminyltransferases (GalNTs) in MCF7 and T47D cells after treatment with Tam, zeocin and hydrogen peroxide (Fig. 6). We investigated the localization of GalNT2 and GalNT6 together with the Golgi-associated protein golgin A1 (Golgin-97) as a marker of the trans-Golgi network after treatment with Tam or zeocin, respectively (Fig. 6a+b). GalNT2 and GalNT6 are preferentially localized in the cis-Golgi apparatus and generate the Tn-antigen by catalyzing the transfer of N-acetylgalactosamine (GalNAc) to serine or threonine residues. Relocation of GALNT2 from Golgi to endoplasmic reticulum enhances Tn antigen expression in breast cancer [35], GalNT6 is upregulated in a majority of breast cancers and is initiating the *O*-glycosylation of MUC 1 [36]. In MCF7 cells, GalNT2 and GalNT6 translocated from cis-Golgi to trans-Golgi network after treatment with Tam or zeocin, respectively, accompanied by an increase of levels of GalNT6 expression. In untreated T47D GalNT2 was already located in trans-Gogi compartment. GalNT6 translocated to the trans-Golgi after treatment with Tam or zeocin; in parallel increased protein levels were observed. In contrast, only minor variations of GalNT2 expression levels were found by Western blot analysis in both cells lines.

### CLEC10A positivity in breast cancer tissue is associated with improved disease free and overall survival

**Fig. 7:**
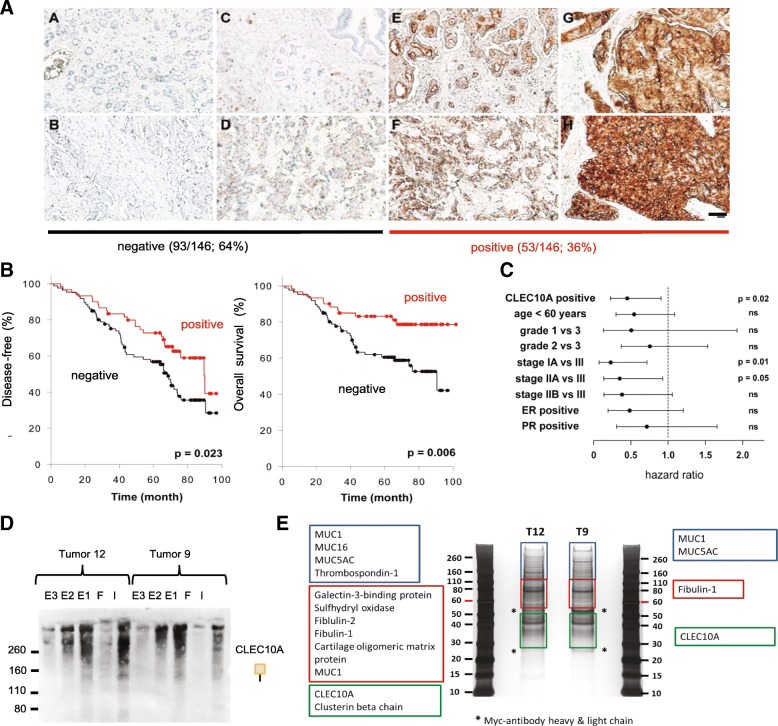
Positivity for CLEC10A ligands is an independent prognostic marker in breast cancer and is associated with favorable outcome. **a** Applying protein histochemistry, 146 paraffin-embedded human breast cancer samples were stained by recombinant CLEC10A. Based on staining intensity and shown by two representative examples, tumors were assigned to the following two groups: no (*A*, *B*) to weak staining (*C*, *D*) and moderate (*E*, *F*) to strong staining (*G*, *H*). Two representative cases are shown for each of the different staining categories. DAB was used for detection and sections were counterstained by hematoxylin. (Scale bar: 100 μm). **b** Log-rank Kaplan Meier analysis of disease-free survival and overall survival of the 146 breast cancer patients. For CLEC10A positive tumors, disease free survival was increased on average by approximately 13 month from 62,1 months (95% confidence interval: 55,2–68,9 months) to 75,8 months (95% ci: 67,5 - 84,2). Overall survival increased by approximately 19 months from 67,5 months (95% ci: 60,1–74,9 months) to 86,7 months (95% ci: 78,6 - 94,8). **c** Hazard ratios of CLEC10A staining and other clinico-pathological parameters for overall survival in breast cancer determined by Cox regression (proportional hazards model); levels of significance are given on the right (ns = not significant). **d** CLEC10A Far Western Blot analysis of input (**i**), flow through (**f**) and eluate fractions (E1-E3) after CLEC10A pull down using two different breast cancer tumors positive for CLEC10A. **e** Silver staining of protein fractions eluted by EDTA after CLEC10A pull down. Gel slices marked by colored boxes were excised and analyzed by mass spectrometry

Given that positive staining for CLEC10A can be induced by hormone depletion or cell damaging agents, we investigated the clinical impact of CLEC10A positivity on breast cancer progression and survival (Fig. 7). For this purpose, paraffin-embedded tissue sections of a representative cohort of 146 breast cancer patients were stained by CLEC10A composed of subtypes of invasive ductal breast cancer comparable to the frequency general frequency of breast cancer subtypes. Tissue was obtained at surgery before any systemic therapy for breast cancer. Patients were treated for early breast cancer without distant metastases by standard hormone therapy or chemotherapy according to guidelines after surgical removal of the carcinoma (Additional file 1: Table S1). Staining intensities of tumor areas were semi-quantitatively assessed by image analysis, and patients were separated in two groups (negative/weak vs. moderate/strong staining for CLEC10A) based on the bimodal distribution of signal intensities applying the Cutoff Finder algorithm (Fig. 7a); group assignments were independently confirmed by two pathologists. Moderate and strong positivity of CLEC10A staining was observed in 36% (*n* = 53) of breast cancer specimens while 64% (*n* = 93) of tumors were weakly positive or stained negative. Kaplan Meier analysis revealed that positive staining by CLEC10A was significantly associated with increased disease free and overall survival (Fig. 7b). For CLEC10A positive tumors, disease free survival was increased on average by approximately 13 month from 62,1 months (95% confidence interval: 55,2–68,9 months) to 75,8 months (95% ci: 67,5 - 84,2). Overall survival increased by approximately 19 months from 67,5 months (95% ci: 60,1–74,9 months) to 86,7 months (95% ci: 78,6 - 94,8). Multivariate Cox regression analysis demonstrated that positivity for CLEC10A is an independent prognostic marker for overall survival (hazard ratio: 0,45 (95% ci: 0,23 - 0,90; Fig. 7c) in addition to advanced stages of breast cancer disease.

For the identification of glycoproteins bound by CLEC10A, pull down experiments with immobilized CLEC10A were performed on whole cellular extracts of two breast cancer samples positively stained by CLEC10A (Fig. 7d). Bound proteins were subsequently eluted and characterized by mass spectrometry (Fig. 7e). Among several glycoproteins of the extracellular matrix such as thrombospondin and fibulin, different members of the mucin protein family (MUC1, MUC5AC and MUC16) were identified, confirming that O-glycan structures recognized by CLEC10A are preferentially attached to mucins in breast cancer cells as previously published [37, 38].

## Discussion

**Fig. 8:**
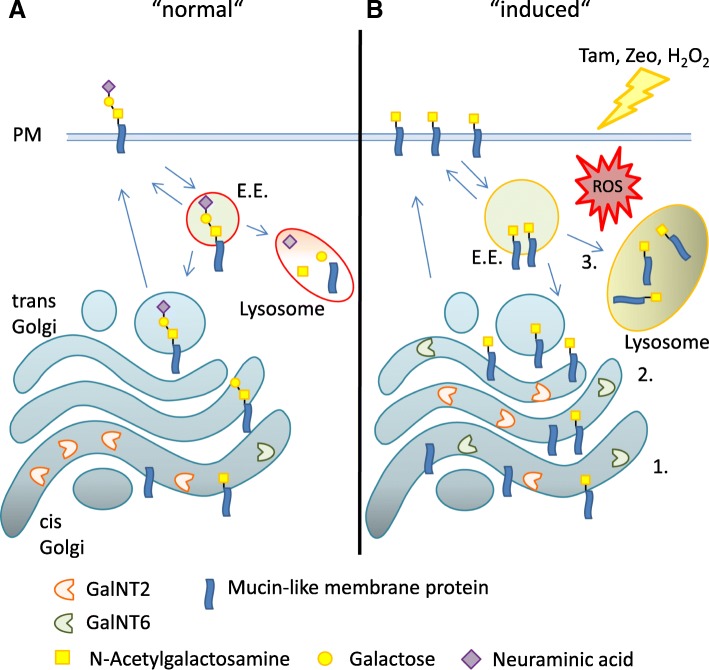
Scheme of the proposed cellular mechanisms involved in the induction and accumulation of CLEC10A ligands. **a** Under normal conditions, glycan structures of newly synthesized mucin-like proteins are elongated in the Golgi compartment and transported to the plasma membrane (PM). For degradation, proteins are internalized and delivered via early endosomes (E.E.) to the lysosome or are recycled. **b** Estrogen depletion, 4-hydroxy-tamoxifen or cell stress inducing substances lead to the accumulation of CLEC10A ligands at the plasma membrane through several mechanisms: 1) increase in levels of acceptor protein such as MUC1, 2) increased expression of GalNT2 and GalNT6 and translocation of GalNTs to the trans Golgi compartment and 3) impaired degradation due to dysfunctional endosomes and lysosomes

CLEC10A is a human glycoreceptor expressed on macrophages and dendritic cells [15, 16]. Major ligands of CLEC10A in human normal and neoplastic tissues are the Tn and sialyl Tn structures. Here we demonstrate that estrogen depletion and treatment of breast cancer cell lines by Tam, zeocin and hydrogen peroxide lead to the enhanced presentation of CLEC10A ligands. Since each of these compounds caused an increase of ROS, reactive oxygen species may be a common denominator in the induction of CLEC10A ligands, resulting in a complex interplay of biosynthesis and degradation, both at the glycan and protein levels (Fig. 8). On the level of synthesis, we observed increased protein levels of GalNT6 known to synthesize the Tn-antigen after treatment of breast cancer cells. GalNT6 is up-regulated in breast and ovarian cancer initiating the *O*-glycosylation of MUC1 [36], which we identified as a major ligand of CLEC10A (Fig. 7 d + e). In addition to the increased protein levels, we observed a shift of GalNT6 from the cis to the trans-Golgi compartment in MCF7 and T47D cells, whereas translocation of GalNT2 was observed in MCF7 only. These alterations may result in incomplete O-glycosylation and accumulation of immature glycoproteins on the plasma membrane. Comparable results regarding changes in GALNTs compartmentalization and levels of Tn-expression were observed in breast cancer cells after stimulation by growth factors [35].

The enzymes of the Golgi *N*- and *O*-glycosylation pathways form enzymatically active homo- and/or heteromeric complexes [39]. Complex formation required for efficient synthesis of cell surface glycans is dependent on Golgi acidity, and elevated Golgi pH correlates with the expression of T-antigen in breast and colorectal cancer cells [13]. Thus, the alkalization of acidic organelles most pronounced in our experiments after Tam treatment may affect the activity of glycosyltransferases involved in glycan chain elongation. Moreover, disruption of the Golgi organization observed after zeocin treatment may disturb protein sorting and transport of membrane proteins.

Enhanced CLEC10A-binding may also be caused by an increase of glycosylated acceptor proteins such as MUC1. A significant increase in MUC1 protein levels was observed after Tam treatment but not by the other agents. Thus, elevated acceptor protein levels appear not to be a general mechanism. It has been reported that enhanced Tn-antigen expression can also be triggered by the loss of COSMC accompanied by a decreased activity of T-synthase [40, 41]. However, our experiments demonstrate that protein levels of COSMC and T-synthase were not reduced after addition of Tam, Zeo or H_2_O_2_, respectively, neither in the MCF7 nor the T47D cell line (Fig. 5 c). Therefore, the accumulation of Tn-antigens is not caused by reduced levels of COSMC and T-synthase, respectively. This result is consistent with the finding that, in the presence of Tam, increased binding of PNA was observed (Fig. 3a). PNA binds to the T antigen, but not to Tn, demonstrating that T-antigen synthesis is not blocked.

Beyond alterations in the synthesis of glycoproteins, defects in the endosomal/lysomal compartments were observed in MCF7 and T47D cells, affecting the degradation and/or recycling of CLEC10A ligands. Increased amounts of the lysosome-specific glycoprotein LAMP2, enlargement of lysosomes, accumulation of autophagy related proteins (LC3b and p62) and increase in pH indicate lysosomal dysfunction resulting in the enrichment of glycosylated membrane proteins. Furthermore, accumulation of undigested material in the lysosome may slow down membrane trafficking and sorting, thereby affecting the endocytosis of membrane proteins from the cell surface. Consequently glycoproteins accumulate at the plasma membrane.

As shown here, the presence of CLEC10A ligands in the tumor tissues of breast cancer patients was associated with a better prognosis compared to patients with low or no expression (Fig. 7b). According to multivariate Cox regression analysis positivity for CLEC10A is an independent prognostic marker for overall survival (Fig. 7c). Studies on breast cancer patients, which rely on the expression of Tn- and/or STn-structures analyzed by antibodies, mostly reported a negative prognostic value of Tn / STn expression [6]. According to Julien et al. data on the prevalence of these structures in tumor tissues vary considerably [14]. The discrepant results were traced back in part to different specificities of antibodies used. The positive correlation between the expression of CLEC10A ligands and patients’ prognosis as described here is supported by a recent study of a spontaneous breast tumor mouse model with a deletion of *C1galt1* in mammary epithelium. In these mice, delayed onset and progression of breast cancer development was observed. As a result of impaired O-glycosylation, tumors of *c1galt1* ^*−/−*^ mice were Tn positive, in contrast to tumors of *c1galt1* wild type mice [42].

## Conclusion

Our results indicate that CLEC10A ligands like Tn- and sialy-Tn are induced in normal and cancerous breast cells by estrogen depletion, 4-hydroxy-tamoxifen-, zeocin- and hydrogen peroxide treatment. We suggest that several molecular and subcellular mechanisms, triggered by ROS generation, are responsible for the accumulation of immature glycostructures due to altered glycosylation and glycan degradation. These changes lead to the accumulation of truncated glycans at the cell surface, which consequently may result in increased phagocytosis by macrophages. Therefore we suppose that CLEC10A is an innate immune receptor for damaged and dead cells, similar to the C-type lectins CLEC9A and CLEC12A [43, 44]. The uptake of damaged cells is mediated by CLEC10A on DC and macrophages during cell renewal and antigen presentation. The biological and immunological functions of CLEC10A and CLEC10A ligands in breast cancer are pertinent questions to be resolved in future studies.

## Additional files

Additional file 1: **Figure S1.** Positivity for CLEC10A ligands correlates with the secretory phase in human endometrium samples. **Figure S2.** Phagocytosis of CFSE-labelled MCF7cells by macrophages. **Figure S3.** Zeocin leads to accumulation of CLEC10A ligands in the endosomal/lysosomal pathway and in autophagosomes. **Figure S4.** Effects of tamoxifen, zeocin and hydrogen peroxide on cell viability of breast cancer cell lines. **Figure S5.** Scratch assay of 4-hydroxy-tamoxifen-, zeocin- and hydrogen peroxide treated breast cancer cell lines. **Figure S6.** Independent reproduction of western blots and mRNA expression analysis of mucin 1 (MUC1), COSMC and T-synthase (T-Syn) in MCF7 and T47D cells. **Figure S7.** Immunofluorescence of GalNT2 and GalNT6 in T47D breast cancer cells. **Table S1.** Clinico-pathological characteristics of the 146 invasive ductal breast carcinomas. **Table S2.** Antibodies and the corresponding dilutions used for western blot and immunofluorescence. (PDF 2784 kb)

## Data Availability

All data generated in this study are included in this publication and its supplementary data files.

## Abbreviations

ER: estrogen receptor
MUC1: Mucin 1
PR: progesterone receptor
STn: sialyl Tn-antigen
T: T-antigen
Tam: 4-hydroxy-tamoxifen
Tn: Tn-antigen
Zeo: zeocin
