# Supplementary material for: Immature O-glycans recognized by the macrophage glycoreceptor CLEC10A (MGL) are induced by 4-hydroxy-tamoxifen, oxidative stress and DNA-damage in breast cancer cells
Source: Cell Commun Signal. 2019 Aug 27;17:107. doi: 10.1186/s12964-019-0420-9 (PMC6712659; doi:10.1186/s12964-019-0420-9)
Supplement: Supplementary file 1 — Figure S1. Positivity for CLEC10A ligands correlates with the secretory phase in human endometrium samples. Figure S2. Phagocytosis of CFSE-labelled MCF7cells by macrophages. Figure S3. Zeocin leads to accumulation of CLEC10A ligands in the endosomal/lysosomal pathway and in autophagosomes. Figure S4. Effects of tamoxifen, zeocin and hydrogen peroxide on cell viability of breast cancer cell lines. Figure S5. Scratch assay of 4-hydroxy-tamoxifen-, zeocin- and hydrogen peroxide treated breast cancer cell lines. Figure S6. Independent reproduction of western blots and mRNA expression analysis of mucin 1 (MUC1), COSMC and T-synthase (T-Syn) in MCF7 and T47D cells. Figure S7. Immunofluorescence of GalNT2 and GalNT6 in T47D breast cancer cells. Table S1. Clinico-pathological characteristics of the 146 invasive ductal breast carcinomas. Table S2. Antibodies and the corresponding dilutions used for western blot and immunofluorescence. (PDF 2784 kb) [file 12964_2019_420_MOESM1_ESM.pdf]

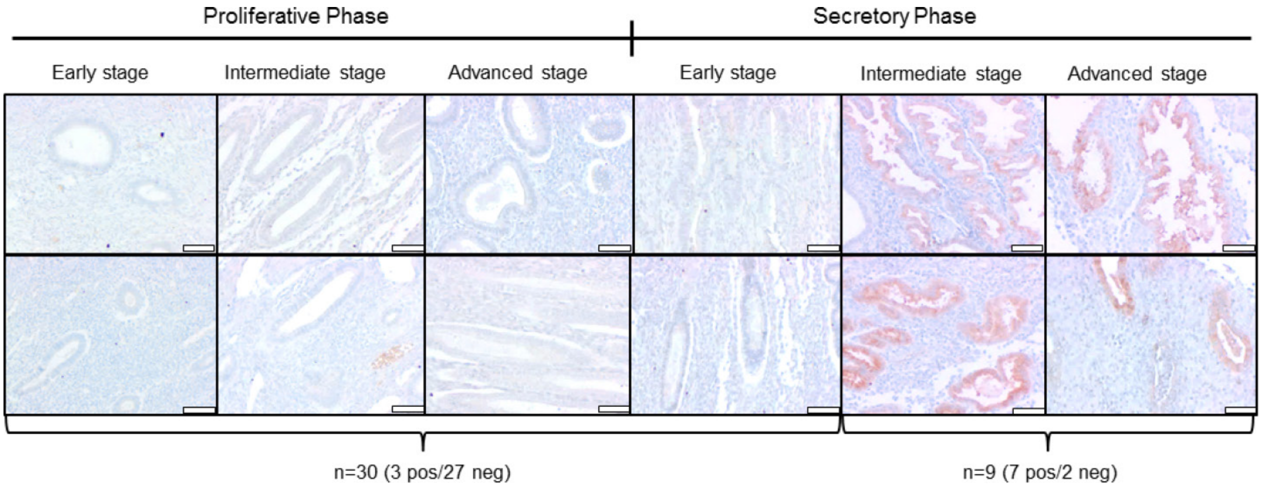

Suppl. Fig. 1: Positivity for CLEC10A ligands correlates with the secretory phase of the female hormonal cycle. 39 premenopausal human endometrium samples were stained by recombinant CLEC10A. 12 representative cases are shown. DAB was used for detection and sections were counterstained by hematoxylin. Scale bar: 100 µm.

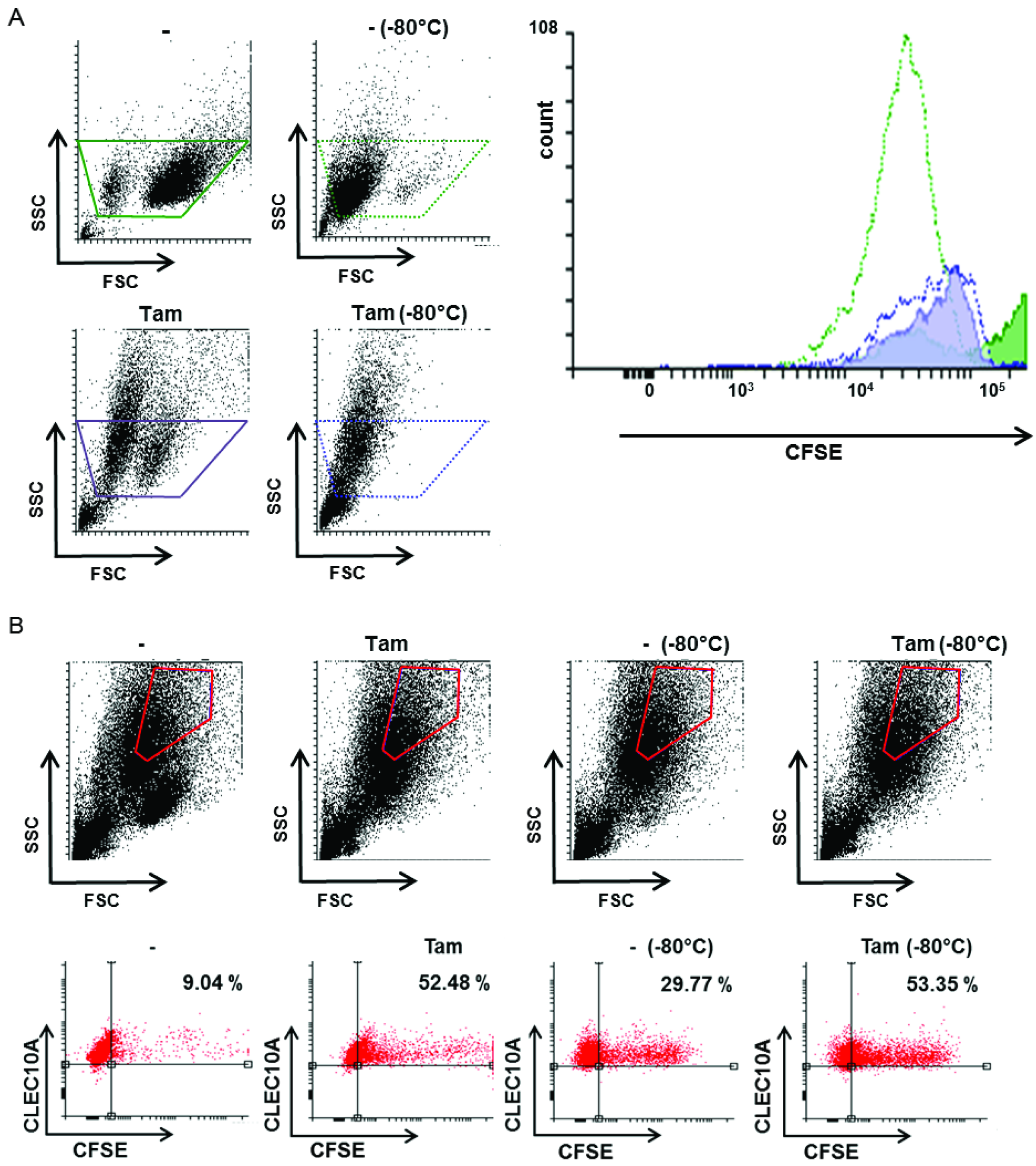

Suppl. Figure 2: Phagocytosis of CFSE-labelled MCF7cells. A) MCF7 cells were treated with tamoxifen (Tam, blue solid line) or ethanol as solvent control (green, solid line). Subsequnetly, cells were labeled with CFSE. Aliquots were killed by freeze and thaw cycles (-80°C). The fluorescence intensity of CFSE labelled cells is shown in the overlay-histogram (-: green filled histogram; Tam: blue filled histogram; -(-80°C): green dotted line; Tam (-80°C): blue dotted line). B) Examples of four representative measurements. Macrophages were incubated for 2 h with CFSE labelled MCF7 cells. CFSE-positive macrophages in the red gate were detected using anti CLEC10A antibody and double positive cells were counted.

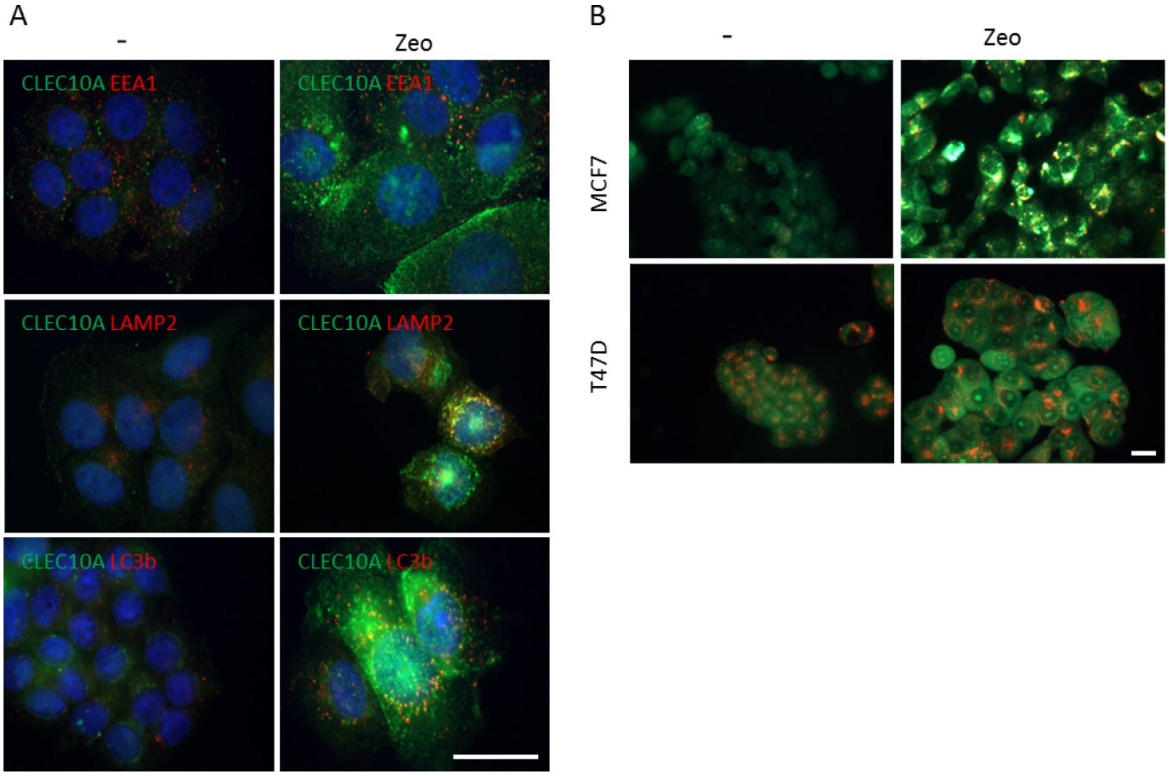

Suppl. Figure 3: Zeocin leads to accumulation of CLEC10A ligands in the endosomal/lysosomal pathway and in autophagosomes. (A) Immunofluorescence staining of CLEC10A ligands (Alexa 488, green), endosomes (early endosomal antigen, EEA1; Alexa 555, red), lysosomes (LAMP2; Alexa 555, red) and autophagosomes (LC3b; Alexa 555, red) in MCF7 cells after 48 h Zeocin treatment. Untreated cells(-) served as control (B) Acridine orange staining of MCF7 and T47D cells treated with Zeocin for 48h showed enlargement of intracellular vesicles. Alkalization of acidic organelles was observed predominantly in MCF7 cells. Sale bar: 20 µm

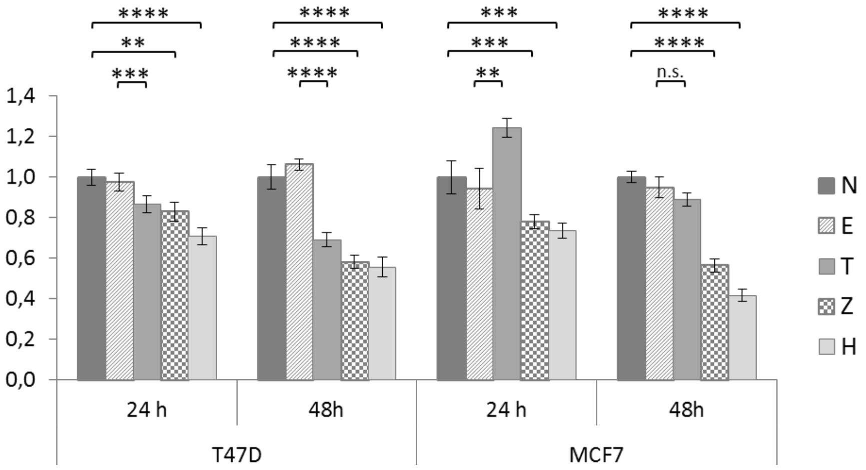

Suppl. Figure 4: Effects of tamoxifen, zeocin and hydrogen peroxide on cell viability. Cells were seeded in sextuplicates and treated with 4 µM tamoxifen (T), 250 µg/ml zeocin (Z) or 30 µM hydrogen peroxide (H) for indicated time periods. Non-treated cells (N) and ethanol treated cells (E) served as controls. Columns show the averages of the replicates normalized to non-treated cells and error bars depict the standard deviation. **:*P* <0.01, *** *P* <0.001, **** *P* <0.0001

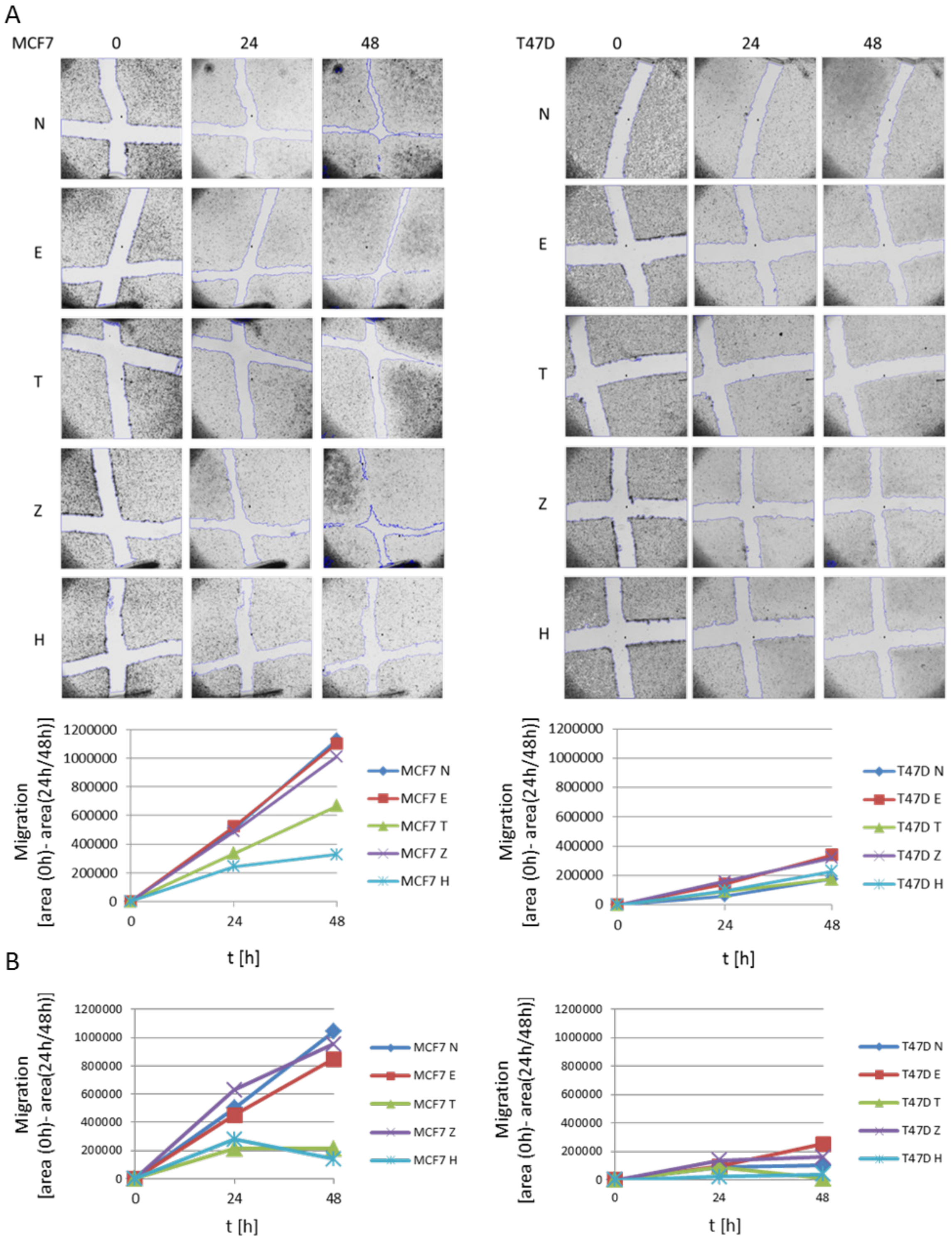

Suppl. Figure 5: Scratch assay: (A) Cells were incubated for 24 h and scratches were created *in vitro* by scraping the cell monolayer with a sterile pipette. Cells were treated with 4 µM tamoxifen (T), 250 µg/ml zeocin (Z) or 30 µM hydrogen peroxide (H) and photographs were taken at the time points indicated. Non-treated cells (N) and ethanol treated cells (E) served as controls. Diagrams show the differences of the scratch areas after 24 h and 48h, respectively. (B) Diagrams show the results of an independent experiment.

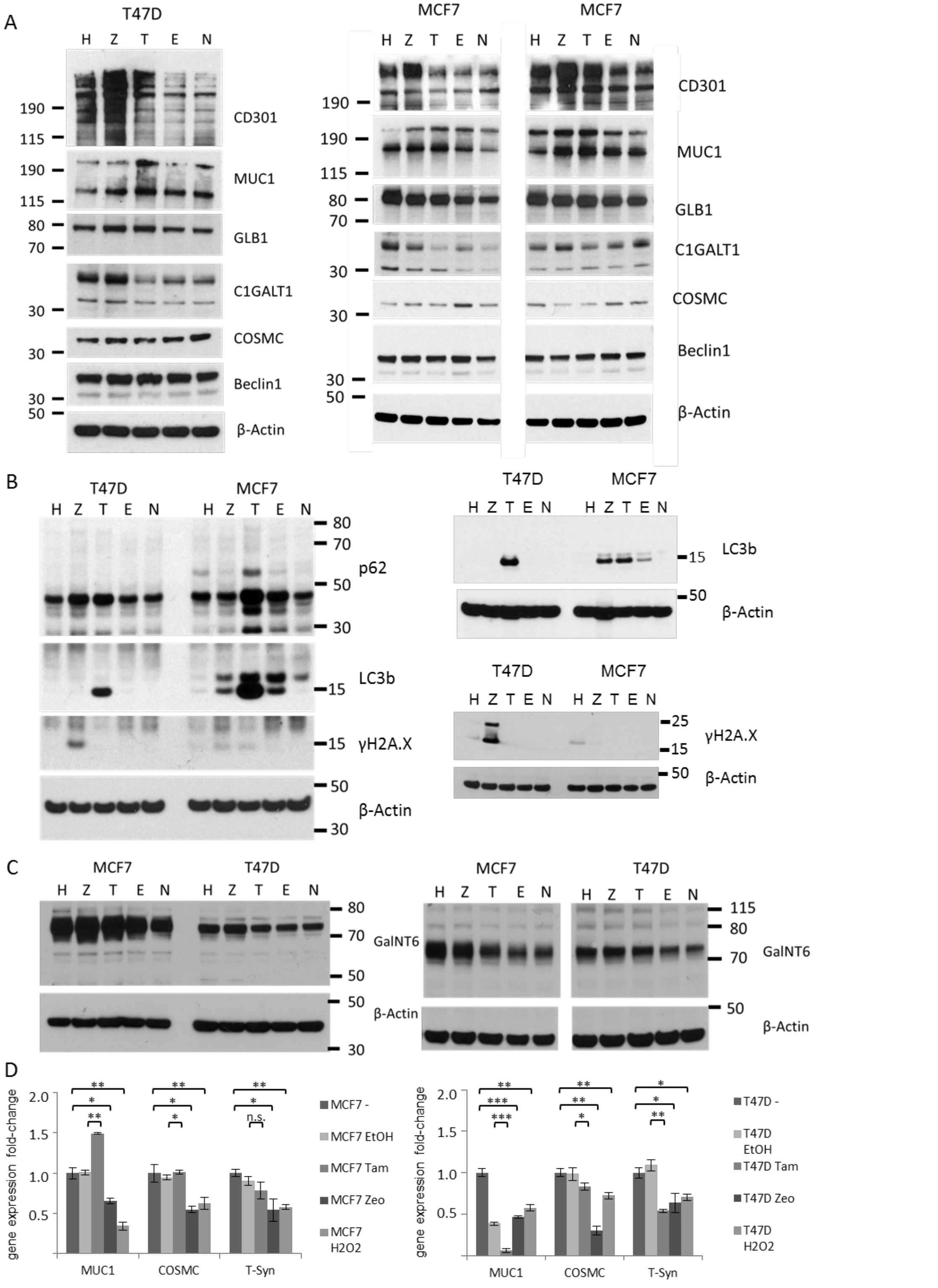

Suppl. Figure 6: (A-C) Independent reproduction of western blots shown in Figure 5 and 6.(D) mRNA expression analysis of mucin 1 (MUC1), COSMC and T-synthase (T-Syn) in MCF7 and T47D cells**.** RT-PCR was performed with cDNA generated from mRNA of untreated (-) cells and cells treated by ethanol (EtOH), Tam, Zeocin (Zeo) or hydrogen peroxide under conditions described above. Mean values were calculated by the $2^{-\Delta\Delta CT}$ method and f*old changes* in gene expression *relative* to the *untreated* control are given. Error bars depict the standard deviations of three technical replicates.

A

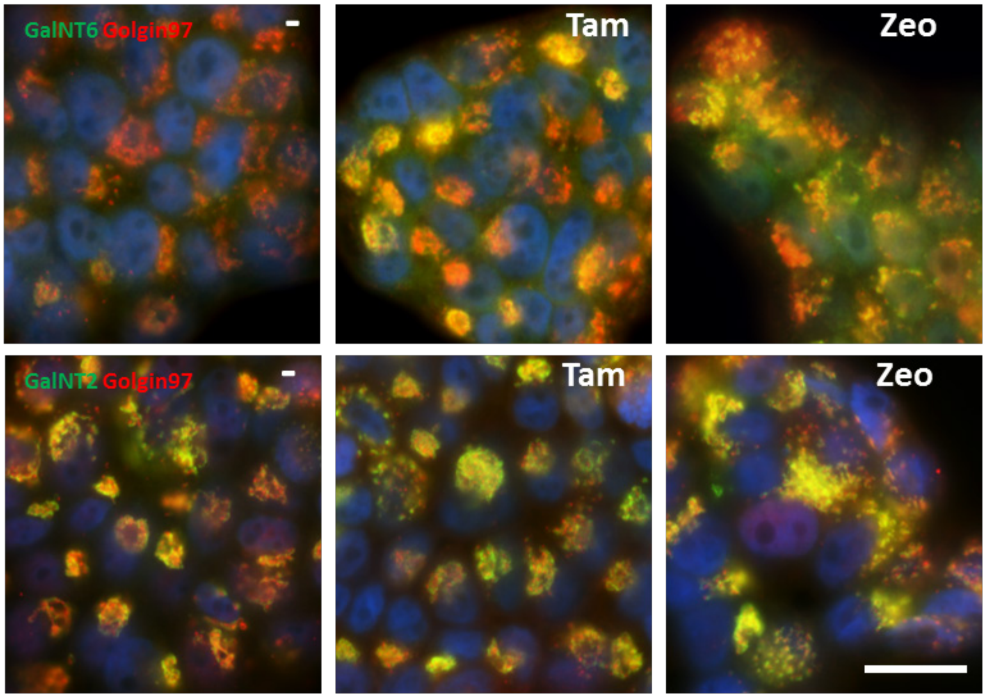

B

GalNTs-Golgin97	M1	M2
T47D - GalNT6	0.36 ± 0.09	0.36 ± 0.09
T47D T GalNT6	0.73 ± 0.09	0.73 ± 0.08
T47D Z GalNT6	0.70 ± 0.05	0.71 ± 0.06
T47D - GalNT2	0.81 ± 0.07	0.81 ± 0.06
T47D T GalNT2	0.80 ± 0.05	0.81 ± 0.06
T47D Z GalNT2	0.77 ± 0.07	0.77 ± 0.06

(Brackets beside table: T47D - GalNT6 vs T47D T GalNT6: ****; T47D - GalNT6 vs T47D Z GalNT6: ****; T47D - GalNT2 vs T47D T GalNT2: n.s.; T47D - GalNT2 vs T47D Z GalNT2: n.s.)

Suppl. Figure 7: Immunofluorescence of GalNT2 and GalNT6 in T47D. (A) Co-localization of GalNT2 and GalNT6 (Alexa 488, green) in the trans-Golgi (Golgin97; Alexa 555, red) in T47D cells after treatment by Tam and Zeocin for 48h, respectively. Predominantly, GalNT6 showed increased expression and shifted towards the trans-Golgi compartment after treatment. Scale bar: 20 µm. (B) Determination of Mander's coefficient for quantification of co-localization from ten images applying JACOP. Averages and standard deviations are given. P-values were calculated with Student's t-test. **** *P*<0.0001.

Material and methods

Suppl. Table 1. Clinico-pathological characteristics of the 146 invasive ductal breast carcinomas. Age is given in years, survival time in month. (ER = estrogen receptor status, PR = progesterone receptor status, neg = negative, pos = positive).

	**median**	**range**
age	60	30-88
survival	65.5	1.6-102
**stage**	**n**	**%**
IA	39	26.7
IIA	52	35.6
IIB	46	31.5
IIIA-C	9	6.2
**grade**	**n**	**%**
1	17	11.6
2	69	47.3
3	60	41.1

**ER**	**n**	**%**
neg	26	17.8
pos	120	82.2
**PR**	**n**	**%**
neg	38	26.0
pos	108	74.0
**Her2/neu**	**n**	**%**
neg	121	82.9
pos	25	17.1

Suppl. Table 2 Antibodies and the corresponding dilutions used for western blot (WB) and immunofluorescence (IF). Methanol [M] or formaldehyde [FA] were used for fixation of cells for immunofluorescence.

Antibody	Source	Dilution WB/ FACS	Dilution IF [Fixation]
Beclin 1	Cell Signaling	1:1000	
COSMC (C1GALT1C1) antibody [N3C3],	Biozol	1:500/-	-
c-Myc Antibody [9E10]	Santa Cruz	1:1000/-	1:100 [FA/M]
c-Myc Antibody [9E10] biotin conjugated	Santa Cruz	1:500/-	1:100[FA/M]
E-Cadherin (24E10)	Cell Signaling	1:1000/-	-
EEA1 [C45B10]	New England BioLabs	-/-	1:200 [FA]
GALNT2 Antibody	Novus Biotech.	1:1000/-	1:250 [FA]
GALNT6 Polyclonal Antibody	Thermo Fisher	1:1000/-	1:250 [FA]
Golgin-97 Monoclonal Antibody [CDF4]	Thermo Fisher Scientific	-	1:200 [FA]
γH2A.X [Phospho-Histone H2A.X (Ser139)] (D7T2V)	Cell Signaling	1:1000	
Her2/ErbB2 [29D8]	Cell Signaling	1:1000/-	-
LAMP2 [2D5]	provided by Prof. Thomas Braulke (University of Hamburg, Germany)	1:1000/-	1:250 [FA]
LC3B [D11] XP®	Cell Signaling	-/-	1:200 [M]
T-Synthase (C1GALT1) antibody	abcam	1:500/-	-
Mucin 1 Antikörper [VU4H5]	Santa Cruz Biotechnologie	1:1000/-	-
APC anti-human CD301 (CLEC10A)	Biolegend	-/ 5µl per 10^6 cells	-
mouse IgG2a, κ APC isotype control	Biolegend	according to manufacturer	
PerCP anti-human CD16 (3G8)	Biolegend	-/ 5µl per 10^6 cells	
PerCP Mouse IgG1, κ isotype control	Biolegend	according to manufacturer	
Goat anti mouse-HRP	Dako	1:25.000/-	-
Goat anti rabbit-HRP	Dako	1:50.000/-	-
Anti-rabbit IgG (H+L), F(ab')2 Fragment (Alexa Fluor® 488 Conjugate)	Cell Signaling	-/-	1:1000
Anti-mouse IgG (H+L), F(ab')2 Fragment (Alexa Fluor® 555 Conjugate)	Cell Signaling	-/-	1:1000

Quantitative real time PCR

Total RNA was extracted from cells of untreated (-) cells and cells treated by ethanol (EtOH), Tam, Zeocin (Zeo) or hydrogen peroxide under conditions described above using phenol/chlorform followed by purification with RNeasy Mini Kit (Quiagen). 1 µg of RNA was reverse transcribed to cDNA using iScript™ Advanced cDNA Synthesis Kit (Biorad). qPCR was performed using LightCycler® 480 Probes Master (Roche) on a Light Cycler 480 II Real-Time PCR System (Roche) under standard conditions. The fold change of mean values was calculated by the $2^{-\Delta\Delta CT}$ method relative to the untreated samples. Target gene levels of three technical replicates were normalized to the housekeeping gene β2-microglobulin (B2M). The following intron-spanning Universal Probe assay primers (P) and probes were used: MUC1 (P1 5'-tcgtagcccctatgagaagg-3'; P2 5'-ccactgctgggtttgtgtaa-3'; Probe 66), T-Synthase (C1GALT1) (P1 5'-ccgcagctgatgtcagaa-3'; P2 5'-gaacataaaagaaatcctattgctgat-3'; Probe 6), COSMC (C1GALT1C) (P1 5'-ttagcgagaccaacgagagaa-3'; P2 5'-accactccgttacgctcct-3'; Probe 65), B2M (P1 5′-ttctggcctggaggctatc-3′; P2 5′-tcaggaaatttgactttccattc-3′; Probe 42)

MTT assay

Viability was determined by a cell titer 96 non-radioactive cell proliferation assay kit (MTT assays) according to the manufacturer's instructions (Promega). Cells (0.5×10^5) were plated in sextuplicate in 96-well plates for 24 h and afterwards incubated with media containing ethanol (E), Tamoxifen (T), zeocin (Z) or hydrogen peroxide (H), respectively. After 24 h or 48h, dye solution was added for 4 h, followed by incubation with lysis buffer at 37 °C overnight. Absorbance was recorded at 570 nm using a Tecan 96-well plate reader with a reference wavelength of 650 nm. Average values and standard deviations were calculated and levels of significance were determined by Student's t-test.

Scratch assay

The migration capabilities of MCF7 and T47D cells were assessed using a scratch wound assay. Cells were seeded into 6-well tissue culture dishes. Scratches were generated in the cell monolayer with a sterile 100 µl plastic pipette tip and cellular debris was removed by washing with phosphate buffer saline (PBS). Cells were treated with 4 µM tamoxifen (T), 250 µg/ml zeocin (Z) or 30 µM hydrogen peroxide (H), respectively. Non-treated (N) and ethanol (E) treated cells served as controls. During treatment DMEM

containing 1% FCS was used to inhibit cell proliferation. Photographs were taken at 0 h, 24 h and 48 h. The migration was calculated from the difference of the areas between 0 h and 24 h or 48 h, respectively.

nanoHPLC-ESI-MS/MS

Protein identification was performed by Proteome Factory (Proteome Factory AG, Berlin, Germany). The LCMS system consisted of an Agilent 1100 nanoHPLC system (Agilent, Waldbronn, Germany), PicoTip electrospray emitter (New Objective, Woburn, MA) and an Orbitrap XL or LTQ-FT Ultra mass spectrometer (ThermoFisher Scientific, Bremen, Germany). Protein spots were in-gel digested by trypsin (Promega, Mannheim, Germany) and analysed by nanoHPLC-ESI-MS/MS. Peptides were first trapped and desalted on the enrichment column (Zorbax 300SB-C18, 0.3 x 5 mm, Agilent) for five minutes (solvent: 2.5% acetonitrile/0.5% formic acid), then separated on a Zorbax 300SB-C18, 75 µm x 150 mm column (Agilent) using a linear gradient from 10% to 32% B (solvent A: 5% acetonitrile in water, solvent B: acetonitrile, both with 0.1% formic acid). Ions of interest were data-dependently subjected to MS/MS according to the expected charge state distribution of peptide ions. Proteins were identified by database search against the NCBInr protein database (National Center for Biotechnology Information,

Sample T12 blue box

Database : HumanUniprot uniprot140506 (136615 sequences; 43285968 residues)
Enzyme : Trypsin
Fixed modifications : Carbamidomethyl (C)
Variable modifications : Deamidated (NQ),Oxidation (M)
Mass values : Monoisotopic
Protein Mass : Unrestricted
Peptide Mass Tolerance : ± 3 ppm
Fragment Mass Tolerance: ± 0.6 Da
Max Missed Cleavages : 1
Instrument type : ESI-TRAP
Number of queries : 1792
Protein hits : **H6VRF8** Keratin 1 OS=Homo sapiens GN=KRT1 PE=3 SV=1
P13645 Keratin, type I cytoskeletal 10 OS=Homo sapiens GN=KRT10 PE=1 SV=6
P35527 Keratin, type I cytoskeletal 9 OS=Homo sapiens GN=KRT9 PE=1 SV=3
P35908 Keratin, type II cytoskeletal 2 epidermal OS=Homo sapiens GN=KRT2 PE=1 SV=2
B1AVQ5 Mucin-1 subunit alpha OS=Homo sapiens GN=MUC1 PE=2 SV=1
Q8WXI7 Mucin-16 OS=Homo sapiens GN=MUC16 PE=1 SV=2
A7Y9J9 Mucin 5AC, oligomeric mucus/gel-forming OS=Homo sapiens GN=MUC5AC PE=4 SV=1
B4E3J7 cDNA FLJ51203, highly similar to Thrombospondin-1 OS=Homo sapiens PE=2 SV=1
Q6MZM7 Putative uncharacterized protein DKFZp686O12165 (Fragment) OS=Homo sapiens GN=DKFZp686O12165 PE=1 SV=1
H7C4T5 Muscleblind-like protein 1 (Fragment) OS=Homo sapiens GN=MBNL1 PE=4 SV=1
A0N4V7 HCG2039797 (Fragment) OS=Homo sapiens GN=Tcr-alpha PE=4 SV=1
L0R5A1 Alternative protein CSF2RB OS=Homo sapiens GN=CSF2RB PE=4 SV=1
I3L4V6 Nucleoredoxin (Fragment) OS=Homo sapiens GN=NXN PE=4 SV=1
Q8WWR7 Desmoglein 2 (Fragment) OS=Homo sapiens GN=DSG2 PE=4 SV=1
Q1RMC9 ERBB2IP protein OS=Homo sapiens GN=ERBB2IP PE=2 SV=1

Select Summary Report
Significance threshold p< Max. number of hits
Standard scoring MudPIT scoring Ions score or expect cut-off Show sub-sets

Show pop-ups Suppress pop-ups Sort unassigned Decreasing Score Require bold red
1. H6VRF8 **Mass:** 66184 **Score:** 676 **Queries matched:** 24 **emPAI:** 0.82
Keratin 1 OS=Homo sapiens GN=KRT1 PE=3 SV=1
Query Observed Mr(expt) Mr(calc) ppm Miss Score Expect Rank Peptide
1007 **437.7527 873.4908 873.4920 -1.29 0 46 0.0011 1 R.SLVNLGGSK.S** 1008
1084 **487.2694 972.5242 972.5240 0.23 0 55 0.00016 1 K.IEISELNR.V** 1083
1118 **517.2611 1032.5077 1032.5087 -1.01 0 51 0.0002 1 R.TLLEGEESR.M**

1203 **590.3042 1178.5939 1178.5931 0.67 0 81 1.8e-007 1 K.YEELQITAGR.H** 1202
1234 **633.3213 1264.6281 1264.6299 -1.47 0 75 6.4e-007 1 R.TNAENEFVTIK.K** 1235
1247 **651.8619 1301.7092 1301.7078 1.03 0 113 8e-011 1 R.SLDLDSIIAEVK.A** 1246 1248
1300 **738.3966 1474.7787 1474.7780 0.48 0 67 3.3e-006 1 R.FLEQQNQVLQTK.W** 1301
1340 **546.9579 1637.8518 1637.8525 -0.45 1 44 0.00053 1 K.SLNNQFASFIDKVR.F** 1339 1341
1447 **538.2747 2149.0698 2149.0705 -0.29 1 43 0.0011 1 R.THNLEPYFESFINNLRR.R** 1448 1449
1489 **762.7154 2285.1243 2285.1175 2.97 1 31 0.013 1 K.AEAESLYQSKYEELQITAGR.H**
1520 **855.7268 2564.1587 2564.1595 -0.33 0 33 0.0062 1 R.MSGECAPNVSVSVSTSHTTISGGGSR.G**
1611 **978.1772 2931.5096 2931.5090 0.20 1 39 0.0019 1 R.FLEQQNQVLQTKWELLQQVDTSTR.T** 1612
Proteins matching the same set of peptides:
H6VRG0 **Mass:** 66101 **Score:** 676 **Queries matched:** 24
Keratin 1 OS=Homo sapiens GN=KRT1 PE=3 SV=1
H6VRG1 **Mass:** 66257 **Score:** 676 **Queries matched:** 24
Keratin 1 OS=Homo sapiens GN=KRT1 PE=2 SV=1
H6VRG3 **Mass:** 66242 **Score:** 676 **Queries matched:** 24
Keratin 1 OS=Homo sapiens GN=KRT1 PE=3 SV=1
P04264 **Mass:** 66170 **Score:** 676 **Queries matched:** 24
Keratin, type II cytoskeletal 1 OS=Homo sapiens GN=KRT1 PE=1 SV=6
2. P13645 **Mass:** 59020 **Score:** 565 **Queries matched:** 17 **emPAI:** 0.59
Keratin, type I cytoskeletal 10 OS=Homo sapiens GN=KRT10 PE=1 SV=6
Query Observed Mr(expt) Mr(calc) ppm Miss Score Expect Rank Peptide
880 **404.2030 806.3915 806.3923 -0.90 0 40 0.0014 1 R.LAADDFR.L** 881
1112 **516.3021 1030.5897 1030.5910 -1.30 0 77 1.5e-007 1 R.VLDELTLTK.A** 1113 1114
1160 **555.2488 1108.4830 1108.4825 0.41 0 63 4.4e-006 1 K.DAEAWFNEK.S**
1213 **601.3120 1200.6094 1200.6098 -0.36 0 60 2.4e-005 1 R.QSVEADINGLR.R** 1212
1233 **631.8030 1261.5915 1261.5899 1.30 0 81 8.6e-008 1 R.SLLEGEGSSGGGGR.G** 1231 1232
1266 **453.5723 1357.6952 1357.6950 0.14 1 51 0.00022 1 R.QSVEADINGLRR.V**
1269 **691.3274 1380.6403 1380.6408 -0.42 0 73 7.7e-007 1 R.ALEESNYELEGK.I**
1286 **478.9281 1433.7625 1433.7626 -0.11 1 35 0.0062 1 K.IRLENEIQTYR.S** 1287
1352 **854.3883 1706.7621 1706.7649 -1.66 0 84 5.1e-008 1 K.GSLGGGFSSGGFSGGSFSR.G** 1353
3. P35527 **Mass:** 62255 **Score:** 484 **Queries matched:** 14 **emPAI:** 0.48
Keratin, type I cytoskeletal 9 OS=Homo sapiens GN=KRT9 PE=1 SV=3
Query Observed Mr(expt) Mr(calc) ppm Miss Score Expect Rank Peptide
1134 **530.7852 1059.5559 1059.5560 -0.16 0 59 4.4e-005 1 K.TLLDIDNTR.M** 1133
1221 **616.8019 1231.5893 1231.5906 -1.03 0 94 6.1e-009 1 R.SGGGGGGGLGSGGSIR.S**
1251 **436.5642 1306.6706 1306.6703 0.24 1 50 0.0002 1 R.IKFEMEQNLR.Q** 1250
1383 **613.3274 1836.9605 1836.9581 1.30 0 59 1.6e-005 1 R.HGVQELEIELQSQLSK.K** 1382
1394 **617.9805 1850.9197 1850.9196 0.03 1 41 0.0013 1 K.TLNDMRQEYEQLIAK.N** 1396
1417 **656.0260 1965.0563 1965.0531 1.65 1 64 2.5e-006 1 R.HGVQELEIELQSQLSKK.A** 1416
1511 **837.3823 2509.1251 2509.1245 0.23 0 73 4.8e-007 1 K.EIETYHNLLEGGQEDFESSGAGK.I** 1510 1629
1088.8422 3263.5047 3263.5066 -0.58 0 46 0.00023 1 K.DIENQYETQITQIEHEVSSSGQEVQSSAK.E
4. P35908 **Mass:** 65678 **Score:** 348 **Queries matched:** 8 **emPAI:** 0.32
Keratin, type II cytoskeletal 2 epidermal OS=Homo sapiens GN=KRT2 PE=1 SV=2
Query Observed Mr(expt) Mr(calc) ppm Miss Score Expect Rank Peptide
1084 487.2694 972.5242 972.5240 0.23 0 55 0.00016 1 K.IEISELNR.V 1083
1121 **519.2669 1036.5193 1036.5189 0.38 0 62 1.4e-005 1 R.YLDGLTAER.T**
1148 **361.5382 1081.5926 1081.5920 0.55 1 22 0.074 1 K.FASFIDKVR.F**
1159 **554.2756 1106.5367 1106.5356 1.04 0 56 6.4e-005 1 K.AQYEEIAQR.S**
1228 **627.8077 1253.6008 1253.6001 0.58 0 87 4.1e-008 1 R.GFSSGSAVVSGGSR.R**
1300 738.3966 1474.7787 1474.7780 0.48 0 67 3.3e-006 1 R.FLEQQNQVLQTK.W 1301
5. B1AVQ5 **Mass:** 49365 **Score:** 142 **Queries matched:** 5 **emPAI:** 0.13
Mucin-1 subunit alpha OS=Homo sapiens GN=MUC1 PE=2 SV=1
Query Observed Mr(expt) Mr(calc) ppm Miss Score Expect Rank Peptide
1181 **567.3202 1132.6258 1132.6241 1.54 0 65 6e-006 1 K.QGGFLGLSNIK.F** 1179 1180
1271 **693.8520 1385.6894 1385.6901 -0.47 0 77 3.8e-007 1 R.DISEMFLQIYK.Q** 1272
Proteins matching the same set of peptides:
B4DWK6 **Mass:** 57362 **Score:** 142 **Queries matched:** 5
cDNA FLJ60927, highly similar to Mucin-1 OS=Homo sapiens PE=2 SV=1
A5YRU5 **Mass:** 30583 **Score:** 142 **Queries matched:** 5
MUC1 isoform Z-LSP OS=Homo sapiens GN=MUC1 PE=2 SV=1
A5YRU7 **Mass:** 23793 **Score:** 142 **Queries matched:** 5
MUC1 isoform T7 OS=Homo sapiens GN=MUC1 PE=2 SV=1
A5YRU8 **Mass:** 26639 **Score:** 142 **Queries matched:** 5
MUC1 isoform T8 OS=Homo sapiens GN=MUC1 PE=2 SV=1

A5YRU9 **Mass:** 12249 **Score:** 142 **Queries matched:** 5
MUC1 isoform T9 OS=Homo sapiens GN=MUC1 PE=4 SV=1
A6ZID5 **Mass:** 29402 **Score:** 142 **Queries matched:** 5
MUC1 isoform M2 OS=Homo sapiens GN=MUC1 PE=2 SV=1
A6ZIE1 **Mass:** 11261 **Score:** 142 **Queries matched:** 5
MUC1 isoform SV4-LSP OS=Homo sapiens GN=MUC1 PE=4 SV=1
B6EC96 **Mass:** 11260 **Score:** 142 **Queries matched:** 5
MUC1 isoform J8 OS=Homo sapiens GN=MUC1 PE=4 SV=1
B6ECA3 **Mass:** 29700 **Score:** 142 **Queries matched:** 5
MUC1 isoform J14 OS=Homo sapiens GN=MUC1 PE=2 SV=1
B6ECB0 **Mass:** 12447 **Score:** 142 **Queries matched:** 5
MUC1 isoform J21 OS=Homo sapiens GN=MUC1 PE=4 SV=1
B6ECB1 **Mass:** 11249 **Score:** 142 **Queries matched:** 5
MUC1 isoform J22 OS=Homo sapiens GN=MUC1 PE=4 SV=1
B6ECB2 **Mass:** 19749 **Score:** 142 **Queries matched:** 5
MUC1 isoform J23 OS=Homo sapiens GN=MUC1 PE=2 SV=1
Q7Z536 **Mass:** 16424 **Score:** 142 **Queries matched:**5
Mucin short variant SV10 OS=Homo sapiens GN=MUC1 PE=2 SV=1
P15941 **Mass:** 122200 **Score:** 142 **Queries matched:** 5
Mucin-1 OS=Homo sapiens GN=MUC1 PE=1 SV=3
6. Q8WXI7 **Mass:** 2359682 **Score:** 140 **Queries matched:** 5 **emPAI:** 0.01
Mucin-16 OS=Homo sapiens GN=MUC16 PE=1 SV=2
Query Observed Mr(expt) Mr(calc) ppm Miss Score Expect Rank Peptide
871 **399.7638 797.5130 797.5123 0.89 0 30 0.0057 1 R.VLQGLLR.P** 870
1136 **532.2740 1062.5335 1062.5346 -0.99 0 24 0.11 1 K.ELGPYTLDR.N**
1252 **654.8198 1307.6250 1307.6258 -0.61 0 54 0.00011 1 R.NSLYVNGFTHR.S**
1267 **679.8878 1357.7611 1357.7605 0.45 0 55 2.9e-005 1 R.GIIELGPYLLDR.G**
7. A7Y9J9 **Mass:** 666520 **Score:** 126 **Queries matched:** 4 **emPAI:** 0.01
Mucin 5AC, oligomeric mucus/gel-forming OS=Homo sapiens GN=MUC5AC PE=4 SV=1
Query Observed Mr(expt) Mr(calc) ppm Miss Score Expect Rank Peptide
1349 **844.3985 1686.7824 1686.7818 0.38 0 78 2.3e-007 1 R.AAGGAVCEQPLGLECR.A** 1350 1351
1619 **1000.1801 2997.5184 2997.5165 0.63 1 56 2.6e-005 1 R.AQAQPGVPLRELGQVVECSLDFGLVCR.N**
Proteins matching the same set of peptides:
E9PBJ0 **Mass:** 611987 **Score:** 126 **Queries matched:** 4
Mucin-5B OS=Homo sapiens GN=MUC5B PE=2 SV=1
Q9HC84 **Mass:** 611584 **Score:** 126 **Queries matched:** 4
Mucin-5B OS=Homo sapiens GN=MUC5B PE=1 SV=3
8. B4E3J7 **Mass:** 124032 **Score:** 113 **Queries matched:** 3 **emPAI:** 0.05
cDNA FLJ51203, highly similar to Thrombospondin-1 OS=Homo sapiens PE=2 SV=1
Query Observed Mr(expt) Mr(calc) ppm Miss Score Expect Rank Peptide
1223 **623.8554 1245.6962 1245.6929 2.67 0 64 4.5e-006 1 R.TIVTTLQDSIR.K** 1222
1319 **517.6005 1549.7796 1549.7750 2.98 0 49 0.00016 1 R.NALWHTGNTPGQVR.T**
Proteins matching the same set of peptides:
Q59E99 **Mass:** 138754 **Score:** 113 **Queries matched:** 3
Thrombospondin 1 variant (Fragment) OS=Homo sapiens PE=2 SV=1
P07996 **Mass:** 133291 **Score:** 113 **Queries matched:** 3
Thrombospondin-1 OS=Homo sapiens GN=THBS1 PE=1 SV=2
9. Q6MZM7 **Mass:** 242944 **Score:** 47 **Queries matched:** 1 **emPAI:** 0.01
Putative uncharacterized protein DKFZp686O12165 (Fragment) OS=Homo sapiens GN=DKFZp686O12165 PE=1 SV=1
Query Observed Mr(expt) Mr(calc) ppm Miss Score Expect Rank Peptide
1356 **866.9787 1731.9428 1731.9407 1.22 0 47 0.00021 1 R.NLQPASEYTVSLVAIK.G**
Proteins matching the same set of peptides:
B7ZLE5 **Mass:**250136 **Score:**47 **Queries matched:**1 FN1 protein
OS=Homo sapiens GN=FN1 PE=2 SV=1
F8W7G7 **Mass:** 246447 **Score:** 47 **Queries matched:** 1
Ugl-Y3 OS=Homo sapiens GN=FN1 PE=2 SV=1
P02751 **Mass:** 266052 **Score:** 47 **Queries matched:** 1
Fibronectin OS=Homo sapiens GN=FN1 PE=1 SV=4
10. H7C4T5 **Mass:** 36596 **Score:** 47 **Queries matched:** 2 **emPAI:** 0.18
Muscleblind-like protein 1 (Fragment) OS=Homo sapiens GN=MBNL1 PE=4 SV=1
Query Observed Mr(expt) Mr(calc) ppm Miss Score Expect Rank Peptide
991 **434.7661 867.5176 867.5178 -0.29 0 25 0.021 1 -.PVSVTPIR.D**
994 **435.7734 869.5323 869.5335 -1.38 0 22 0.055 1 -.VVSVTPIR.D**
11. A0N4V7 **Mass:** 2269 **Score:** 44 **Queries matched:** 14 **emPAI:** 1.75
HCG2039797 (Fragment) OS=Homo sapiens GN=Tcr-alpha PE=4 SV=1
Query Observed Mr(expt) Mr(calc) ppm Miss Score Expect Rank Peptide
939 **421.7581 841.5016 841.5022 -0.64 1 44 0.0008 1 K.GITLSVRP.-** 938 940 941 942 944 945 946 947 948 949 950 951 952
12. L0R5A1 **Mass:** 11752 **Score:** 31 **Queries matched:** 1 **emPAI:** 0.28
Alternative protein CSF2RB OS=Homo sapiens GN=CSF2RB PE=4 SV=1
Query Observed Mr(expt) Mr(calc) ppm Miss Score Expect Rank Peptide
978 **428.7653 855.5160 855.5178 -2.07 0 31 0.013 1 R.LASIVTPR.N**

13. I3L4V6 **Mass:** 26188 **Score:** 26 **Queries matched:** 2 **emPAI:** 0.12
Nucleoredoxin (Fragment) OS=Homo sapiens GN=NXN PE=4 SV=1
Query Observed Mr(expt) Mr(calc) ppm Miss Score Expect Rank Peptide
1376 **913.8992 1825.7838 1825.7854 -0.86 0 26 0.015 1 R.LYGIQDSEDDGESEAAK.Q** 1375
Proteins matching the same set of peptides:
B4DNN6 **Mass:** 14224 **Score:** 26 **Queries matched:** 2
Nucleoredoxin OS=Homo sapiens GN=NXN PE=2 SV=1
14. Q8WWR7 **Score:** 24 **Queries matched:** 1
Desmoglein 2 (Fragment) OS=Homo sapiens GN=DSG2 PE=4 SV=1
Query Observed Mr(expt) Mr(calc) ppm Miss Score Expect Rank Peptide
500 **584.4383 583.4310 583.4309 0.19 0 24 0.0044 1 R.LLLLL.-**
15. Q1RMC9 **Mass:** 154105 **Score:** 23 **Queries matched:** 2
ERBB2IP protein OS=Homo sapiens GN=ERBB2IP PE=2 SV=1
Query Observed Mr(expt) Mr(calc) ppm Miss Score Expect Rank Peptide
1138 **533.2573 1064.5000 1064.5026 -2.42 0 23 0.077 1 K.YPNLESVNK.V** 1139

Proteins matching the same set of peptides:
Q96RT1 **Mass:** 158941 **Score:** 23 **Queries matched:** 2
Protein LAP2 OS=Homo sapiens GN=ERBB2IP PE=1 SV=2

Sample: T12 red box

Database : HumanUniprot uniprot140506 (136615 sequences; 43285968 residues)
Enzyme : Trypsin
Fixed modifications : Carbamidomethyl (C)
Variable modifications : Deamidated (NQ),Oxidation (M)
Mass values : Monoisotopic
Protein Mass : Unrestricted
Peptide Mass Tolerance : ± 3 ppm
Fragment Mass Tolerance: ± 0.6 Da
Max Missed Cleavages : 1
Instrument type : ESI-TRAP
Number of queries : 2296
Protein hits : **H6VRF8** Keratin 1 OS=Homo sapiens GN=KRT1 PE=3 SV=1
H6VRG2 Keratin 1 OS=Homo sapiens GN=KRT1 PE=3 SV=1
P13645 Keratin, type I cytoskeletal 10 OS=Homo sapiens GN=KRT10 PE=1 SV=6
P35527 Keratin, type I cytoskeletal 9 OS=Homo sapiens GN=KRT9 PE=1 SV=3 **P35908** Keratin, type II cytoskeletal 2 epidermal OS=Homo sapiens GN=KRT2 PE=1 SV=2
B4DVE1 cDNA FLJ53478, highly similar to Galectin-3-binding protein OS=Homo sapiens PE=2 SV=1
P08779 Keratin, type I cytoskeletal 16 OS=Homo sapiens GN=KRT16 PE=1 SV=4
P02533 Keratin, type I cytoskeletal 14 OS=Homo sapiens GN=KRT14 PE=1 SV=4
P04259 Keratin, type II cytoskeletal 6B OS=Homo sapiens GN=KRT6B PE=1 SV=5
A8K477 Sulfhydryl oxidase OS=Homo sapiens PE=2 SV=1
P13647 Keratin, type II cytoskeletal 5 OS=Homo sapiens GN=KRT5 PE=1 SV=3 **F5H1F3** Fibulin-2 OS=Homo sapiens GN=FBLN2 PE=2 SV=1
P23142 Fibulin-1 OS=Homo sapiens GN=FBLN1 PE=1 SV=4
G3XAP6 Cartilage oligomeric matrix protein OS=Homo sapiens GN=COMP PE=4 SV=1
B1AVQ5 Mucin-1 subunit alpha OS=Homo sapiens GN=MUC1 PE=2 SV=1
H7C4T5 Muscleblind-like protein 1 (Fragment) OS=Homo sapiens GN=MBNL1 PE=4 SV=1
A0N4V7 HCG2039797 (Fragment) OS=Homo sapiens GN=Tcr-alpha PE=4 SV=1 **Q2VPJ6** HSP90AA1 protein (Fragment) OS=Homo sapiens GN=HSP90AA1 PE=1 SV=1 **L0R5A1** Alternative protein CSF2RB OS=Homo sapiens GN=CSF2RB PE=4 SV=1 **Q8WWR7** Desmoglein 2 (Fragment) OS=Homo sapiens GN=DSG2 PE=4 SV=1
Q86TT1 Full-length cDNA clone CS0DD006YL02 of Neuroblastoma of Homo sapiens (human) OS=Homo sapiens PE=2 SV=1
B7Z945 Protein LOC100132891 OS=Homo sapiens GN=LOC100132891 PE=2 SV=1

Select Summary Report

Significance threshold p< Max. number of hits
Standard scoring MudPIT scoring Ions score or expect cut-off Show sub-sets
Show pop-ups Suppress pop-ups Sort unassigned Decreasing Score Require bold red
1. H6VRF8 **Mass:** 66184 **Score:** 1598 **Queries matched:** 66 **emPAI:** 2.65
Keratin 1 OS=Homo sapiens GN=KRT1 PE=3 SV=1
Query Observed Mr(expt) Mr(calc) ppm Miss Score Expect Rank Peptide
999 **437.7531 873.4917 873.4920 -0.30 0 49 0.00057 1 R.SLVNLGGSK.S** 1000 1001
1117 **487.2697 972.5248 972.5240 0.86 0 62 2.7e-005 1 K.IEISELNR.V** 1116 1118
1149 **500.2269 998.4392 998.4379 1.33 0 58 2.2e-005 1 K.DVDGAYMTK.V** 1148 1150
1190 **517.2629 1032.5113 1032.5087 2.51 0 57 8.3e-005 1 R.TLLEGEESR.M** 1191
1227 **533.2651 1064.5157 1064.5138 1.74 0 47 0.00047 1 K.AQYEDIAQK.S** 1225 1226
1283 **563.2755 1124.5364 1124.5349 1.28 0 80 1.7e-007 1 K.AEAESLYQSK.Y** 1284

1308 **571.2647 1140.5149 1140.5121 2.47 0 54 5.7e-005 1 R.DYQELMNTK.L** 1309 1310
1353 **590.3044 1178.5942 1178.5931 0.91 0 82 1.7e-007 1 K.YEELQITAGR.H** 1351 1352
1409 **633.3224 1264.6303 1264.6299 0.30 0 69 2.9e-006 1 R.TNAENEFVTIK.K** 1408 1410
1419 **639.3590 1276.7035 1276.7027 0.62 0 79 1.6e-007 1 K.LALDLEIATYR.T** 1418 1420
1430 **651.8619 1301.7092 1301.7078 1.03 0 106 4.3e-010 1 R.SLDLDSIIAEVK.A** 1431
1463 **670.8391 1339.6637 1339.6619 1.33 1 81 1.8e-007 1 K.SKAEAESLYQSK.Y** 1459 1460 1461 1462
1501 **692.3487 1382.6829 1382.6830 -0.08 0 86 5.2e-008 1 K.SLNNQFASFIDK.V** 1500 1502
1507 **697.3693 1392.7241 1392.7249 -0.53 1 67 2.7e-006 1 R.TNAENEFVTIKK.D** 1508 1509 1510 1511
1554 **738.3784 1474.7423 1474.7416 0.49 0 75 5.7e-007 1 K.WELLQQVDTSTR.T** 1555 1556
1557 **738.3983 1474.7820 1474.7780 2.71 0 93 9e-009 1 R.FLEQQNQVLQTK.W** 1558
1608 **508.6007 1522.7804 1522.7813 -0.63 1 41 0.001 1 R.LLRDYQELMNTK.L** 1609
1668 **546.9582 1637.8528 1637.8525 0.16 1 49 0.00015 1 K.SLNNQFASFIDKVR.F** 1667 1669
1673 **829.4014 1656.7883 1656.7856 1.61 0 91 1.8e-008 1 R.SGGGFSSGSAGIINYQR.R**
1799 **648.0022 1940.9846 1940.9803 2.24 1 51 0.00011 1 K.LNDLEDALQQAKEDLAR.L** 1798
1821 **662.6357 1984.8852 1984.8870 -0.93 1 45 0.00047 1 R.LDSELKNMQDMVEDYR.N**
1858 **538.2763 2149.0760 2149.0705 2.59 1 40 0.0023 1 R.THNLEPYFESFINNLRR.R** 1854 1857
1930 **777.0620 2328.1642 2328.1669 -1.17 1 40 0.0019 1 K.QISNLQQSISDAEQRGENALK.D**
1978 **855.7286 2564.1641 2564.1595 1.78 0 41 0.00085 1 R.MSGECAPNVSVSVSTSHTTISGGGSR.G** 1977
2072 **978.1786 2931.5138 2931.5090 1.64 1 54 6.2e-005 1 R.FLEQQNQVLQTKWELLQQVDTSTR.T** 2070 2071
Proteins matching the same set of peptides:
H6VRG0 **Mass:** 66101 **Score:** 1598 **Queries matched:** 66
Keratin 1 OS=Homo sapiens GN=KRT1 PE=3 SV=1
H6VRG1 **Mass:** 66257 **Score:** 1598 **Queries matched:** 66
Keratin 1 OS=Homo sapiens GN=KRT1 PE=2 SV=1
H6VRG3 **Mass:** 66242 **Score:** 1598 **Queries matched:** 66
Keratin 1 OS=Homo sapiens GN=KRT1 PE=3 SV=1
P04264 **Mass:** 66170 **Score:** 1598 **Queries matched:** 66
Keratin, type II cytoskeletal 1 OS=Homo sapiens GN=KRT1 PE=1 SV=6
2. H6VRG2 **Mass:** 66197 **Score:** 1574 **Queries matched:** 66 **emPAI:** 2.65
Keratin 1 OS=Homo sapiens GN=KRT1 PE=3 SV=1
Query Observed Mr(expt) Mr(calc) ppm Miss Score Expect Rank Peptide
999 437.7531 873.4917 873.4920 -0.30 0 49 0.00057 1 R.SLVNLGGSK.S 1000 1001
1117 487.2697 972.5248 972.5240 0.86 0 62 2.7e-005 1 K.IEISELNR.V 1116 1118
1149 500.2269 998.4392 998.4379 1.33 0 58 2.2e-005 1 K.DVDGAYMTK.V 1148 1150
1190 517.2629 1032.5113 1032.5087 2.51 0 57 8.3e-005 1 R.TLLEGEESR.M 1191
1227 533.2651 1064.5157 1064.5138 1.74 0 47 0.00047 1 K.AQYEDIAQK.S 1225 1226
1283 563.2755 1124.5364 1124.5349 1.28 0 80 1.7e-007 1 K.AEAESLYQSK.Y 1284
1308 571.2647 1140.5149 1140.5121 2.47 0 54 5.7e-005 1 R.DYQELMNTK.L 1309 1310
1353 590.3044 1178.5942 1178.5931 0.91 0 82 1.7e-007 1 K.YEELQITAGR.H 1351 1352
1409 633.3224 1264.6303 1264.6299 0.30 0 69 2.9e-006 1 R.TNAENEFVTIK.K 1408 1410
1419 639.3590 1276.7035 1276.7027 0.62 0 79 1.6e-007 1 K.LALDLEIATYR.T 1418 1420
1453 **665.3673 1328.7201 1328.7187 1.06 0 83 9.6e-008 1 R.NLDLDSIIAEVK.A** 1454
1463 670.8391 1339.6637 1339.6619 1.33 1 81 1.8e-007 1 K.SKAEAESLYQSK.Y 1459 1460 1461 1462
1501 692.3487 1382.6829 1382.6830 -0.08 0 86 5.2e-008 1 K.SLNNQFASFIDK.V 1500 1502
1507 697.3693 1392.7241 1392.7249 -0.53 1 67 2.7e-006 1 R.TNAENEFVTIKK.D 1508 1509 1510 1511
1554 738.3784 1474.7423 1474.7416 0.49 0 75 5.7e-007 1 K.WELLQQVDTSTR.T 1555 1556
1557 738.3983 1474.7820 1474.7780 2.71 0 93 9e-009 1 R.FLEQQNQVLQTK.W 1558
1608 508.6007 1522.7804 1522.7813 -0.63 1 41 0.001 1 R.LLRDYQELMNTK.L 1609
1668 546.9582 1637.8528 1637.8525 0.16 1 49 0.00015 1 K.SLNNQFASFIDKVR.F 1667 1669
1673 829.4014 1656.7883 1656.7856 1.61 0 91 1.8e-008 1 R.SGGGFSSGSAGIINYQR.R
1799 648.0022 1940.9846 1940.9803 2.24 1 51 0.00011 1 K.LNDLEDALQQAKEDLAR.L 1798
1821 662.6357 1984.8852 1984.8870 -0.93 1 45 0.00047 1 R.LDSELKNMQDMVEDYR.N
1858 538.2763 2149.0760 2149.0705 2.59 1 40 0.0023 1 R.THNLEPYFESFINNLRR.R 1854 1857
1930 777.0620 2328.1642 2328.1669 -1.17 1 40 0.0019 1 K.QISNLQQSISDAEQRGENALK.D
1978 855.7286 2564.1641 2564.1595 1.78 0 41 0.00085 1 R.MSGECAPNVSVSVSTSHTTISGGGSR.G 1977
2072 978.1786 2931.5138 2931.5090 1.64 1 54 6.2e-005 1 R.FLEQQNQVLQTKWELLQQVDTSTR.T 2070 2071
3. P13645 **Mass:** 59020 **Score:** 1214 **Queries matched:** 50 **emPAI:** 1.96
Keratin, type I cytoskeletal 10 OS=Homo sapiens GN=KRT10 PE=1 SV=6
Query Observed Mr(expt) Mr(calc) ppm Miss Score Expect Rank Peptide
841 **404.2035 806.3924 806.3923 0.12 0 58 2.5e-005 1 R.LAADDFR.L** 842 843
852 **405.2239 808.4332 808.4330 0.17 0 51 0.00013 1 R.LASYLDK.V** 853
1135 **497.2542 992.4939 992.4927 1.28 0 57 5.3e-005 1 K.YENEVALR.Q** 1133 1134
1139 **332.5112 994.5118 994.5123 -0.51 1 32 0.0034 1 K.IKEWYEK.H**
1157 **502.2809 1002.5472 1002.5458 1.45 1 24 0.098 1 K.SEITELRR.N** 1155 1156
1186 **516.3024 1030.5902 1030.5910 -0.77 0 66 2e-006 1 R.VLDELTLTK.A** 1187 1188
1221 **532.8085 1063.6024 1063.6026 -0.17 1 63 5.5e-006 1 R.LASYLDKVR.A** 1220 1222 1224
1250 **545.7702 1089.5259 1089.5237 2.07 0 74 7.9e-007 1 K.VTMQNLNDR.L** 1249 1251
1265 **555.2493 1108.4840 1108.4825 1.37 0 64 4e-006 1 K.DAEAWFNEK.S** 1263 1264
1341 **583.2965 1164.5784 1164.5775 0.84 0 56 7.6e-005 1 R.LENEIQTYR.S** 1340
1375 **601.3118 1200.6091 1200.6098 -0.59 0 55 7.9e-005 1 R.QSVEADINGLR.R**
1396 **617.8427 1233.6708 1233.6717 -0.75 1 64 5.2e-006 1 R.LKYENEVALR.Q**
1406 **631.8035 1261.5924 1261.5899 2.04 0 97 2.3e-009 1 R.SLLEGEGSSGGGGR.G** 1405 1407
1478 **453.2443 1356.7110 1356.7110 0.05 1 (38) 0.0034 1 R.QSVEADINGLRR.V** 1479

1482 **453.5725 1357.6958 1357.6950 0.61 1 66 5.3e-006 1 R.QSVEADINGLRR.V** 1483
1487 **683.3245 1364.6345 1364.6320 1.81 0 69 1.8e-006 1 R.SQYEQLAEQNR.K**
1498 **691.3282 1380.6419 1380.6408 0.79 0 80 1.4e-007 1 R.ALEESNYELEGK.I** 1496 1497
1530 **478.9291 1433.7654 1433.7626 1.96 1 35 0.0052 1 K.IRLENEIQTYR.S** 1528 1529
1593 **498.5828 1492.7267 1492.7270 -0.20 1 40 0.0019 1 R.SQYEQLAEQNRK.D** 1594
1694 **854.3899 1706.7653 1706.7649 0.21 0 121 1e-011 1 K.GSLGGGFSSGGFSGGSFSR.G** 1695
1892 **738.0382 2211.0926 2211.0906 0.92 1 43 0.00059 1 K.SKELTTEIDNNIEQISSYK.S** 1891 1893
4. P35527 **Mass:** 62255 **Score:** 892 **Queries matched:** 47 **emPAI:** 1.42
Keratin, type I cytoskeletal 9 OS=Homo sapiens GN=KRT9 PE=1 SV=3
Query Observed Mr(expt) Mr(calc) ppm Miss Score Expect Rank Peptide
852 405.2239 808.4332 808.4330 0.17 0 51 0.00013 1 R.LASYLDK.V 853
1033 **449.2099 896.4052 896.4062 -1.14 0 44 0.0006 1 R.MTLDDFR.I** 1032
1218 **530.7855 1059.5564 1059.5560 0.39 0 71 2.3e-006 1 K.TLLDIDNTR.M** 1216 1217
1331 **579.2995 1156.5845 1156.5836 0.72 0 (76) 4.3e-007 1** 1332
1334 **579.7911 1157.5677 1157.5677 0.05 0 97 2.6e-009 1 R.QGVDADINGLR.Q** 1333 1335
1364 **595.8081 1189.6016 1189.6013 0.27 0 54 7.8e-005 1 R.QVLDNLTMEK.S** 1363 1365
1392 **616.8011 1231.5877 1231.5906 -2.33 0 124 4.8e-012 1 R.SGGGGGGGLGSGGSIR.S** 1393 1394
1436 **654.3436 1306.6727 1306.6703 1.85 1 (40) 0.001 1 R.IKFEMEQNLR.Q** 1433 1434 1435 1437
1444 **441.8963 1322.6671 1322.6652 1.40 1 50 0.00013 1 R.IKFEMEQNLR.Q** 1443 1445
1746 **919.4888 1836.9631 1836.9581 2.70 0 123 6.3e-012 1 R.HGVQELEIELQSQLSK.K** 1742 1743 1744 1745
1758 **617.9810 1850.9213 1850.9196 0.92 1 50 0.00019 1 K.TLNDMRQEYEQLIAK.N** 1759 1760 1761 1762
1809 **656.0253 1965.0541 1965.0531 0.52 1 73 2.8e-007 1 R.HGVQELEIELQSQLSKK.A** 1808 1810
1921 **767.0453 2298.1141 2298.1123 0.81 1 27 0.035 1 K.SDLEMQYETLQEELMALKK.N**
1959 **837.3819 2509.1239 2509.1245 -0.23 0 61 7.3e-006 1 K.EIETYHNLLEGGQEDFESSGAGK.I** 1958 1960
2109 **1088.8426 3263.5060 3263.5066 -0.18 0 67 1.8e-006 1 K.DIENQYETQITQIEHEVSSSGQEVQSSAK.E** 2108 2110 2111
5. P35908 **Mass:** 65678 **Score:** 713 **Queries matched:** 27 **emPAI:** 0.75
Keratin, type II cytoskeletal 2 epidermal OS=Homo sapiens GN=KRT2 PE=1 SV=2
Query Observed Mr(expt) Mr(calc) ppm Miss Score Expect Rank Peptide
909 **416.2511 830.4877 830.4862 1.83 0 50 0.00029 1 R.SLVGLGGTK.S**
1117 487.2697 972.5248 972.5240 0.86 0 62 2.7e-005 1 K.IEISELNR.V 1116 1118
1193 **519.2667 1036.5188 1036.5189 -0.14 0 62 1.5e-005 1 R.YLDGLTAER.T** 1192 1194
1241 **541.8034 1081.5923 1081.5920 0.29 1 65 3.5e-006 1 K.FASFIDKVR.F** 1239 1240 1242 1243 1244
1261 **554.2759 1106.5372 1106.5356 1.46 0 61 2.3e-005 1 K.AQYEEIAQR.S** 1260 1262
1401 **627.8083 1253.6021 1253.6001 1.65 0 110 1.7e-010 1 R.GFSSGSAVVSGGSR.R** 1400
1441 **440.8661 1319.5765 1319.5756 0.66 0 60 7.7e-006 1 R.HGGGGGGFGGGGFGSR.S** 1440
1453 665.3673 1328.7201 1328.7187 1.06 0 83 9.6e-008 1 R.NLDLDSIIAEVK.A 1454
1542 **730.9045 1459.7945 1459.7922 1.58 0 68 1.1e-006 1 K.VDLLNQEIEFLK.V** 1543 1544
1557 738.3983 1474.7820 1474.7780 2.71 0 93 9e-009 1 R.FLEQQNQVLQTK.W 1558
6. B4DVE1 **Mass:** 64911 **Score:** 526 **Queries matched:** 23 **emPAI:** 0.68
cDNA FLJ53478, highly similar to Galectin-3-binding protein OS=Homo sapiens PE=2 SV=1
Query Observed Mr(expt) Mr(calc) ppm Miss Score Expect Rank Peptide
705 **368.1773 734.3401 734.3388 1.79 0 31 0.017 1 R.YFYSR.R** 703 704
896 **413.7272 825.4398 825.4385 1.61 0 30 0.01 1 R.VEIFYR.G** 894 895
1178 **343.8490 1028.5251 1028.5251 0.07 0 43 0.00077 1 R.STHTLDLSR.E** 1179
1302 **566.3411 1130.6677 1130.6659 1.58 1 49 5.2e-005 1 R.RIDITLSSVK.C** 1301
1379 **603.7814 1205.5483 1205.5465 1.50 0 51 9.5e-005 1 K.AVDTWSWGER.A**
1451 **663.8321 1325.6496 1325.6463 2.47 0 53 9.8e-005 1 R.ASHEEVEGLVEK.I** 1447 1448 1449 1450
1468 **678.3938 1354.7730 1354.7708 1.65 0 71 4.9e-007 1 R.SDLAVPSELALLK.A** 1467
1645 **796.8997 1591.7849 1591.7842 0.45 0 67 4.1e-006 1 R.ELSEALGQIFDSQR.G**
1817 **662.0402 1983.0988 1983.0942 2.31 0 69 5.9e-007 1 K.TLQALEFHTVPFQLLAR.Y** 1815 1816
1861 **1081.5122 2161.0099 2161.0116 -0.82 0 62 1e-005 1 R.IYTSPTWSAFVTDSSWSAR.K**
Proteins matching the same set of peptides:
Q08380 **Mass:** 66202 **Score:** 526 **Queries matched:** 23
Galectin-3-binding protein OS=Homo sapiens GN=LGALS3BP PE=1 SV=1
7. P08779 **Mass:** 51578 **Score:** 443 **Queries matched:** 17 **emPAI:** 0.60
Keratin, type I cytoskeletal 16 OS=Homo sapiens GN=KRT16 PE=1 SV=4
Query Observed Mr(expt) Mr(calc) ppm Miss Score Expect Rank Peptide
841 404.2035 806.3924 806.3923 0.12 0 58 2.5e-005 1 R.LAADDFR.T 842 843
852 405.2239 808.4332 808.4330 0.17 0 51 0.00013 1 R.LASYLDK.V 853
1182 **515.3016 1028.5886 1028.5866 1.91 0 62 1.8e-005 1 R.VLDELTLAR.T** 1181
1221 532.8085 1063.6024 1063.6026 -0.17 1 63 5.5e-006 1 R.LASYLDKVR.A 1220 1222 1224
1250 545.7702 1089.5259 1089.5237 2.07 0 74 7.9e-007 1 K.VTMQNLNDR.L 1249 1251
1427 **651.3332 1300.6519 1300.6510 0.69 0 64 1.2e-005 1 R.ALEEANADLEVK.I** 1428
1457 **669.8362 1337.6579 1337.6575 0.30 0 70 1.2e-006 1 R.APSTYGGGLSVSSR.F**
8. P02533 **Mass:** 51872 **Score:** 433 **Queries matched:** 18 **emPAI:** 0.60
Keratin, type I cytoskeletal 14 OS=Homo sapiens GN=KRT14 PE=1 SV=4
Query Observed Mr(expt) Mr(calc) ppm Miss Score Expect Rank Peptide
841 404.2035 806.3924 806.3923 0.12 0 58 2.5e-005 1 R.LAADDFR.T 842 843
852 405.2239 808.4332 808.4330 0.17 0 51 0.00013 1 R.LASYLDK.V 853
1182 515.3016 1028.5886 1028.5866 1.91 0 62 1.8e-005 1 R.VLDELTLAR.A 1181
1221 532.8085 1063.6024 1063.6026 -0.17 1 63 5.5e-006 1 R.LASYLDKVR.A 1220 1222 1224
1250 545.7702 1089.5259 1089.5237 2.07 0 74 7.9e-007 1 K.VTMQNLNDR.L 1249 1251

1427 651.3332 1300.6519 1300.6510 0.69 0 64 1.2e-005 1 R.ALEEANADLEVK.I 1428
1524 **713.3537 1424.6928 1424.6896 2.24 0 61 2e-005 1 R.APSTYGGGLSVSSSR.F** 1523
9. P04259 **Mass:** 60315 **Score:** 390 **Queries matched:** 17 **emPAI:** 0.43
Keratin, type II cytoskeletal 6B OS=Homo sapiens GN=KRT6B PE=1 SV=5
Query Observed Mr(expt) Mr(calc) ppm Miss Score Expect Rank Peptide
1084 **473.2590 944.5034 944.5039 -0.52 1 36 0.015 1 R.GRLDSELR.N**
1241 541.8034 1081.5923 1081.5920 0.29 1 65 3.5e-006 1 K.FASFIDKVR.F 1239 1240 1242 1243 1244
1261 554.2759 1106.5372 1106.5356 1.46 0 61 2.3e-005 1 K.AQYEEIAQR.S 1260 1262
1353 590.3044 1178.5942 1178.5931 0.91 0 82 1.7e-007 1 K.YEELQITAGR.H 1351 1352
1377 **602.3231 1202.6317 1202.6295 1.77 0 64 8e-006 1 K.WTLLQEQGTK.T** 1376
1453 665.3673 1328.7201 1328.7187 1.06 0 83 9.6e-008 1 R.NLDLDSIIAEVK.A 1454
10. A8K477 **Mass:** 67567 **Score:** 389 **Queries matched:** 14 **emPAI:** 0.31
Sulfhydryl oxidase OS=Homo sapiens PE=2 SV=1
Query Observed Mr(expt) Mr(calc) ppm Miss Score Expect Rank Peptide
1073 **465.7577 929.5009 929.5004 0.47 0 46 0.00013 1 R.VPVLMESR.S** 1072 1074
1273 **557.8134 1113.6123 1113.6142 -1.69 0 68 3.2e-006 1 R.VLNTEANVVR.K** 1274 1275
1469 **678.8437 1355.6729 1355.6721 0.61 0 77 3.1e-007 1 R.LAGAPSEDPQFPK.V** 1470 1471
1564 **741.8782 1481.7418 1481.7402 1.11 0 63 9.1e-006 1 R.NNEEYLALIFEK.G** 1563
1780 **631.9962 1892.9668 1892.9646 1.16 0 25 0.049 1 R.VGSPNAAVLWLWSSHNR.V**
1836 **1024.0394 2046.0642 2046.0633 0.41 0 109 1.4e-010 1 R.SALYSPSDPLTLLQADTVR.G** 1835
Proteins matching the same set of peptides:
A8K4C2 **Mass:** 67521 **Score:** 389 **Queries matched:** 14
Sulfhydryl oxidase OS=Homo sapiens PE=2 SV=1
O00391 **Mass:** 83324 **Score:** 389 **Queries matched:** 14
Sulfhydryl oxidase 1 OS=Homo sapiens GN=QSOX1 PE=1 SV=3
11. P13647 **Mass:** 62568 **Score:** 386 **Queries matched:** 16 **emPAI:** 0.41
Keratin, type II cytoskeletal 5 OS=Homo sapiens GN=KRT5 PE=1 SV=3
Query Observed Mr(expt) Mr(calc) ppm Miss Score Expect Rank Peptide
1084 473.2590 944.5034 944.5039 -0.52 1 36 0.015 1 R.GRLDSELR.N
1241 541.8034 1081.5923 1081.5920 0.29 1 65 3.5e-006 1 K.FASFIDKVR.F 1239 1240 1242 1243 1244
1255 **547.2669 1092.5192 1092.5199 -0.69 0 61 1.2e-005 1 K.AQYEEIANR.S** 1256
1269 **556.2909 1110.5672 1110.5669 0.27 0 77 2.9e-007 1 R.ISISTSGGSFR.N** 1268 1270
1377 602.3231 1202.6317 1202.6295 1.77 0 64 8e-006 1 K.WTLLQEQGTK.T 1376
1453 665.3673 1328.7201 1328.7187 1.06 0 83 9.6e-008 1 R.NLDLDSIIAEVK.A 1454
12. F5H1F3 **Mass:** 135130 **Score:** 329 **Queries matched:** 11 **emPAI:** 0.17
Fibulin-2 OS=Homo sapiens GN=FBLN2 PE=2 SV=1
Query Observed Mr(expt) Mr(calc) ppm Miss Score Expect Rank Peptide
945 **422.2289 842.4433 842.4432 0.06 0 29 0.0054 1 R.NPLICAR.G** 946
1210 **526.2931 1050.5717 1050.5710 0.66 0 48 0.00018 1 R.QGSVTTFLAK.M** 1211
1290 **565.2484 1128.4822 1128.4836 -1.23 0 58 8.5e-006 1 K.GNEEGYFGTR.R**
1388 **614.3169 1226.6193 1226.6158 2.80 0 26 0.036 1 K.MHIFFTTFAL.-**
1513 **698.8848 1395.7551 1395.7510 2.89 0 80 1e-007 1 R.LNAYTGVVYLQR.A**
1550 **734.3514 1466.6883 1466.6864 1.30 0 25 0.05 1 R.FECPPNYVQVSK.T**
1726 **906.5167 1811.0189 1811.0193 -0.20 0 64 1.2e-006 1 R.IGPAPAFTGDTIALNIIK.G** 1727 1728
Proteins matching the same set of peptides:
B7Z6T9 **Mass:** 60778 **Score:** 329 **Queries matched:** 11
cDNA FLJ50237, highly similar to Homo sapiens fibulin 2 (FBLN2), transcript variant 1, mRNA OS=Homo sapiens PE=2 SV=1
B7Z9B8 **Mass:** 135058 **Score:** 329 **Queries matched:** 11
cDNA FLJ56912, highly similar to Fibulin-2 OS=Homo sapiens PE=2 SV=1
Q9Y3V7 **Mass:** 66767 **Score:** 329 **Queries matched:** 11
Putative uncharacterized protein DKFZp586A1519 (Fragment) OS=Homo sapiens GN=DKFZp586A1519 PE=2 SV=1
P98095 **Mass:** 132248 **Score:** 329 **Queries matched:** 11
Fibulin-2 OS=Homo sapiens GN=FBLN2 PE=1 SV=2
13. P23142 **Mass:** 81268 **Score:** 249 **Queries matched:** 10 **emPAI:** 0.35
Fibulin-1 OS=Homo sapiens GN=FBLN1 PE=1 SV=4
Query Observed Mr(expt) Mr(calc) ppm Miss Score Expect Rank Peptide
1137 **497.2719 992.5292 992.5291 0.14 1 33 0.012 1 R.DSFDIIKR.Y**
1387 **614.2972 1226.5798 1226.5788 0.82 0 78 2.5e-007 1 R.YMDGMTVGVVR.Q** 1385 1386
1397 **622.2946 1242.5747 1242.5737 0.80 0 (58) 2.1e-005 1 R.YMDGMTVGVVR.Q**
1416 **635.7854 1269.5562 1269.5547 1.21 0 33 0.0054 1 K.DCSLPYATESK.E**
1620 **775.4236 1548.8326 1548.8300 1.66 1 25 0.029 1 R.EFTRPEEIIFLR.A** 1619
1627 **520.6062 1558.7969 1558.7926 2.77 0 33 0.01 1 K.LEMNYVVGGVVSHR.N**
1951 **825.7732 2474.2977 2474.2917 2.41 0 47 0.00016 1 R.AITPPHPASQANIIFDITEGNLR.D**
14. G3XAP6 **Mass:** 82212 **Score:** 171 **Queries matched:** 8 **emPAI:** 0.12
Cartilage oligomeric matrix protein OS=Homo sapiens GN=COMP PE=4 SV=1
Query Observed Mr(expt) Mr(calc) ppm Miss Score Expect Rank Peptide
1174 **513.3044 1024.5943 1024.5917 2.55 0 50 9.2e-005 1 R.AVAEPGIQLK.A** 1172 1173
1575 **743.8788 1485.7430 1485.7423 0.47 0 82 1.4e-007 1 R.ELQETNAALQDVR.E** 1576 1577
1654 **538.5930 1612.7571 1612.7594 -1.39 0 40 0.0011 1 R.NALWHTGDTESQVR.L** 1655
Proteins matching the same set of peptides:
B4DKJ3 **Mass:** 79446 **Score:** 171 **Queries matched:** 8

Cartilage oligomeric matrix protein OS=Homo sapiens GN=COMP PE=2 SV=1
B4DN90 **Mass:** 83983 **Score:** 171 **Queries matched:** 8
cDNA FLJ53494, highly similar to Cartilage oligomeric matrix protein OS=Homo sapiens PE=2 SV=1
A8K3I0 **Mass:** 85457 **Score:** 171 **Queries matched:** 8
cDNA FLJ78437, highly similar to Homo sapiens cartilage oligomeric matrix protein (COMP), mRNA OS=Homo sapiens PE=2 SV=1
Q53FR6 **Mass:** 85345 **Score:** 171 **Queries matched:** 8
Cartilage oligomeric matrix protein variant (Fragment) OS=Homo sapiens PE=2 SV=1
P49747 **Mass:** 85431 **Score:** 171 **Queries matched:** 8
Cartilage oligomeric matrix protein OS=Homo sapiens GN=COMP PE=1 SV=2
15. B1AVQ5 **Mass:** 49365 **Score:** 73 **Queries matched:** 2 **emPAI:** 0.06
Mucin-1 subunit alpha OS=Homo sapiens GN=MUC1 PE=2 SV=1
Query Observed Mr(expt) Mr(calc) ppm Miss Score Expect Rank Peptide
1504 **693.8528 1385.6910 1385.6901 0.65 0 73 9.8e-007 1 R.DISEMFLQIYK.Q** 1503
Proteins matching the same set of peptides:
B4DWK6 **Mass:** 57362 **Score:** 73 **Queries matched:** 2
cDNA FLJ60927, highly similar to Mucin-1 OS=Homo sapiens PE=2 SV=1
A5YRU5 **Mass:** 30583 **Score:** 73 **Queries matched:** 2
MUC1 isoform Z-LSP OS=Homo sapiens GN=MUC1 PE=2 SV=1
A5YRU7 **Mass:** 23793 **Score:** 73 **Queries matched:** 2
MUC1 isoform T7 OS=Homo sapiens GN=MUC1 PE=2 SV=1
A5YRU8 **Mass:** 26639 **Score:** 73 **Queries matched:** 2
MUC1 isoform T8 OS=Homo sapiens GN=MUC1 PE=2 SV=1
A5YRU9 **Mass:** 12249 **Score:** 73 **Queries matched:** 2
MUC1 isoform T9 OS=Homo sapiens GN=MUC1 PE=4 SV=1
A6ZID5 **Mass:** 29402 **Score:** 73 **Queries matched:** 2
MUC1 isoform M2 OS=Homo sapiens GN=MUC1 PE=2 SV=1
A6ZIE1 **Mass:** 11261 **Score:** 73 **Queries matched:** 2
MUC1 isoform SV4-LSP OS=Homo sapiens GN=MUC1 PE=4 SV=1
B6EC96 **Mass:** 11260 **Score:** 73 **Queries matched:** 2
MUC1 isoform J8 OS=Homo sapiens GN=MUC1 PE=4 SV=1
B6ECA3 **Mass:** 29700 **Score:** 73 **Queries matched:** 2
MUC1 isoform J14 OS=Homo sapiens GN=MUC1 PE=2 SV=1
B6ECA9 **Mass:** 13226 **Score:** 73 **Queries matched:** 2
MUC1 isoform J20 OS=Homo sapiens GN=MUC1 PE=2 SV=1
B6ECB0 **Mass:** 12447 **Score:** 73 **Queries matched:** 2
MUC1 isoform J21 OS=Homo sapiens GN=MUC1 PE=4 SV=1
B6ECB1 **Mass:** 11249 **Score:** 73 **Queries matched:** 2
MUC1 isoform J22 OS=Homo sapiens GN=MUC1 PE=4 SV=1
B6ECB2 **Mass:** 19749 **Score:** 73 **Queries matched:** 2
MUC1 isoform J23 OS=Homo sapiens GN=MUC1 PE=2 SV=1
Q7Z536 **Mass:** 16424 **Score:** 73 **Queries matched:** 2
Mucin short variant SV10 OS=Homo sapiens GN=MUC1 PE=2 SV=1
P15941 **Mass:** 122200 **Score:** 73 **Queries matched:** 2
Mucin-1 OS=Homo sapiens GN=MUC1 PE=1 SV=3
16. H7C4T5 **Mass:** 36596 **Score:** 53 **Queries matched:** 3 **emPAI:** 0.09
Muscleblind-like protein 1 (Fragment) OS=Homo sapiens GN=MBNL1 PE=4 SV=1
Query Observed Mr(expt) Mr(calc) ppm Miss Score Expect Rank Peptide
987 **435.7737 869.5329 869.5335 -0.70 0 29 0.012 1 -.VVSVTPIR.D** 993
1038 **450.2695 898.5244 898.5236 0.84 0 24 0.082 1 -.QVSVTPIR.D**
17. A0N4V7 **Mass:** 2269 **Score:** 42 **Queries matched:** 17 **emPAI:** 6.57
HCG2039797 (Fragment) OS=Homo sapiens GN=Tcr-alpha PE=4 SV=1
Query Observed Mr(expt) Mr(calc) ppm Miss Score Expect Rank Peptide
941 **421.7592 841.5039 841.5022 2.08 1 42 0.0014 1 K.GITLSVRP.-** 926 927 928 929 930 932 933 934 935 936 937 938 939 940 942 943
18. Q2VPJ6 **Mass:** 68614 **Score:** 38 **Queries matched:** 1 **emPAI:** 0.05
HSP90AA1 protein (Fragment) OS=Homo sapiens GN=HSP90AA1 PE=1 SV=1
Query Observed Mr(expt) Mr(calc) ppm Miss Score Expect Rank Peptide
1319 **576.2839 1150.5532 1150.5506 2.31 0 38 0.0038 1 K.YIDQEELNK.T**
Proteins matching the same set of peptides:
B4DGL0 **Mass:** 82518 **Score:** 38 **Queries matched:** 1
cDNA FLJ53619, highly similar to Heat shock protein HSP 90-beta OS=Homo sapiens PE=2 SV=1
B4DMA2 **Mass:** 79487 **Score:** 38 **Queries matched:** 1
cDNA FLJ54023, highly similar to Heat shock protein HSP 90-beta OS=Homo sapiens PE=2 SV=1
Q6PK50 **Mass:** 40270 **Score:** 38 **Queries matched:** 1
HSP90AB1 protein (Fragment) OS=Homo sapiens GN=HSP90AB1 PE=2 SV=1
Q8TBA7 **Mass:** 74179 **Score:** 38 **Queries matched:** 1
HSP90AA1 protein (Fragment) OS=Homo sapiens GN=HSP90AA1 PE=2 SV=2
K9JA46 **Mass:** 85006 **Score:** 38 **Queries matched:** 1
Epididymis luminal secretory protein 52 OS=Homo sapiens GN=EL52 PE=2 SV=1
Q58FF8 **Mass:** 44492 **Score:** 38 **Queries matched:** 1
Putative heat shock protein HSP 90-beta 2 OS=Homo sapiens GN=HSP90AB2P PE=1 SV=2
Q86U12 **Mass:** 49669 **Score:** 38 **Queries matched:** 1

Full-length cDNA clone CS0CAP007YF18 of Thymus of Homo sapiens (human) OS=Homo sapiens GN=HSP90AA1 PE=2 SV=1
P07900 **Mass:** 85006 **Score:** 38 **Queries matched:** 1
Heat shock protein HSP 90-alpha OS=Homo sapiens GN=HSP90AA1 PE=1 SV=5
P08238 **Mass:** 83554 **Score:** 38 **Queries matched:** 1
Heat shock protein HSP 90-beta OS=Homo sapiens GN=HSP90AB1 PE=1 SV=4
Q14568 **Mass:** 39454 **Score:** 38 **Queries matched:** 1
Putative heat shock protein HSP 90-alpha A2 OS=Homo sapiens GN=HSP90AA2 PE=1 SV=2
O75322 **Mass:** 63839 **Score:** 38 **Queries matched:** 1
Hsp89-alpha-delta-N OS=Homo sapiens PE=2 SV=1
19. L0R5A1 **Mass:** 11752 **Score:** 34 **Queries matched:** 6 **emPAI:** 0.63
Alternative protein CSF2RB OS=Homo sapiens GN=CSF2RB PE=4 SV=1
Query Observed Mr(expt) Mr(calc) ppm Miss Score Expect Rank Peptide
965 **428.7660 855.5174 855.5178 -0.40 0 34 0.0055 1 R.LASIVTPR.N** 962 963 967 968 969
20. Q8WWR7 **Score:** 26 **Queries matched:** 2
Desmoglein 2 (Fragment) OS=Homo sapiens GN=DSG2 PE=4 SV=1
Query Observed Mr(expt) Mr(calc) ppm Miss Score Expect Rank Peptide
428 **584.4394 583.4321 583.4309 2.09 0 26 0.0023 1 R.LLLLL.-** 427
21. Q86TT1 **Mass:** 41817 **Score:** 23 **Queries matched:** 1 **emPAI:** 0.08
Full-length cDNA clone CS0DD006YL02 of Neuroblastoma of Homo sapiens (human) OS=Homo sapiens PE=2 SV=1
Query Observed Mr(expt) Mr(calc) ppm Miss Score Expect Rank Peptide
1665 **819.4687 1636.9228 1636.9229 -0.05 0 23 0.016 1 R.VFAIPPSFASIFLTK.S**
Proteins matching the same set of peptides:
P01871 **Mass:** 49960 **Score:** 23 **Queries matched:** 1
Ig mu chain C region OS=Homo sapiens GN=IGHM PE=1 SV=3
P04220 **Mass:** 43543 **Score:** 23 **Queries matched:** 1
Ig mu heavy chain disease protein OS=Homo sapiens PE=1 SV=1
22. B7Z945 **Mass:** 20378 **Score:** 23 **Queries matched:** 3 **emPAI:** 0.16
Protein LOC100132891 OS=Homo sapiens GN=LOC100132891 PE=2 SV=1
Query Observed Mr(expt) Mr(calc) ppm Miss Score Expect Rank Peptide
1425 **648.3393 1294.6641 1294.6629 0.88 1 23 0.057 1 R.VGAQVQNPRAPR.K** 1423 1424

Sample T12 green box

Database : HumanUniprot uniprot140506 (136615 sequences; 43285968 residues)
Enzyme : Trypsin
Fixed modifications : Carbamidomethyl (C)
Variable modifications : Deamidated (NQ),Oxidation (M)
Mass values : Monoisotopic
Protein Mass : Unrestricted
Peptide Mass Tolerance : ± 3 ppm
Fragment Mass Tolerance: ± 0.6 Da
Max Missed Cleavages : 1
Instrument type : ESI-TRAP
Number of queries : 2179
Protein hits : **H6VRF8** Keratin 1 OS=Homo sapiens GN=KRT1 PE=3 SV=1
Q1KLZ0 HCG15971, isoform CRA_a OS=Homo sapiens GN=PS1TP5BP1 PE=2 SV=1 **P13645** Keratin, type I cytoskeletal 10 OS=Homo sapiens GN=KRT10 PE=1 SV=6
P35527 Keratin, type I cytoskeletal 9 OS=Homo sapiens GN=KRT9 PE=1 SV=3 **D2JYH4** Actin, alpha 2, smooth muscle, aorta OS=Homo sapiens GN=ACTA2 PE=2 SV=1
Q2TSD0 Glyceraldehyde-3-phosphate dehydrogenase OS=Homo sapiens PE=2 SV=1
J3KR22 C-type lectin domain family 10 member A OS=Homo sapiens GN=CLEC10A PE=2 SV=1
P35908 Keratin, type II cytoskeletal 2 epidermal OS=Homo sapiens GN=KRT2 PE=1 SV=2
P04259 Keratin, type II cytoskeletal 6B OS=Homo sapiens GN=KRT6B PE=1 SV=5
B4DE59 cDNA FLJ60424, highly similar to Junction plakoglobin OS=Homo sapiens PE=2 SV=1
P02533 Keratin, type I cytoskeletal 14 OS=Homo sapiens GN=KRT14 PE=1 SV=4
P08779 Keratin, type I cytoskeletal 16 OS=Homo sapiens GN=KRT16 PE=1 SV=4
Q1KSF8 XTP3TPA-transactivated protein 1 OS=Homo sapiens GN=XTP3TPATP1 PE=2 SV=1
B4DNE0 cDNA FLJ52573, highly similar to Elongation factor 1-alpha 1 OS=Homo sapiens PE=2 SV=1
Q7L4M3 KRT8 protein OS=Homo sapiens PE=2 SV=1
H0YAS8 Clusterin beta chain (Fragment) OS=Homo sapiens GN=CLU PE=4 SV=1 **H0YKS4** Annexin (Fragment) OS=Homo sapiens GN=ANXA2 PE=3 SV=1
A6NE09 40S ribosomal protein SA OS=Homo sapiens GN=RPSAP58 PE=3 SV=1 **F8VV57** Keratin, type II cytoskeletal 5 (Fragment) OS=Homo sapiens GN=KRT5 PE=2 SV=1
Q0Z944 Beta globin (Fragment) OS=Homo sapiens GN=HBB PE=2 SV=1
H7C4T5 Muscleblind-like protein 1 (Fragment) OS=Homo sapiens GN=MBNL1 PE=4 SV=1
A0N4V7 HCG2039797 (Fragment) OS=Homo sapiens GN=Tcr-alpha PE=4 SV=1 **Q9NSB2** Keratin, type II cuticular Hb4 OS=Homo sapiens GN=KRT84 PE=2 SV=2
Q1ZYQ1 Tubulin, alpha 2 OS=Homo sapiens GN=TUBA2 PE=2 SV=1
L0R5A1 Alternative protein CSF2RB OS=Homo sapiens GN=CSF2RB PE=4 SV=1 **B7Z945** Protein LOC100132891 OS=Homo sapiens GN=LOC100132891 PE=2 SV=1 **Q59E90** Mannosidase, alpha, class 2B, member 1 variant (Fragment) OS=Homo sapiens PE=2 SV=1

Select Summary Report
Significance threshold p< Max. number of hits
Standard scoring MudPIT scoring Ions score or expect cut-off Show sub-sets
Show pop-ups Suppress pop-ups Sort unassigned Decreasing Score Require bold red

1. H6VRF8 **Mass:** 66184 **Score:** 932 **Queries matched:** 34 **emPAI:** 1.19
Keratin 1 OS=Homo sapiens GN=KRT1 PE=3 SV=1
Query Observed Mr(expt) Mr(calc) ppm Miss Score Expect Rank Peptide
754 **416.7487 831.4829 831.4814 1.80 0 41 0.0015 1 K.SISISVAR.G**
960 **487.2694 972.5242 972.5240 0.28 0 59 5.3e-005 1 K.IEISELNR.V** 959
992 **500.2267 998.4388 998.4379 0.90 0 50 0.00015 1 K.DVDGAYMTK.V** 991 993
1015 **517.2624 1032.5102 1032.5087 1.38 0 60 3.6e-005 1 R.TLLEGEESR.M** 1017
1144 **590.3038 1178.5931 1178.5931 -0.02 0 84 8.4e-008 1 K.YEELQITAGR.H** 1145 1146
1208 **633.3213 1264.6281 1264.6299 -1.43 0 69 2.3e-006 1 R.TNAENEFVTIK.K** 1209 1210
1215 **639.3587 1276.7027 1276.7027 0.06 0 74 5e-007 1 K.LALDLEIATYR.T**
1229 **651.8602 1301.7059 1301.7078 -1.45 0 108 2.4e-010 1 R.SLDLDSIIAEVK.A** 1228 1230
1267 **692.3483 1382.6821 1382.6830 -0.68 0 80 1.6e-007 1 K.SLNNQFASFIDK.V** 1266 1268
1273 **465.2482 1392.7228 1392.7249 -1.46 1 45 0.00045 1 R.TNAENEFVTIKK.D**
1308 **738.3981 1474.7816 1474.7780 2.48 0 71 1.2e-006 1 R.FLEQQNQVLQTK.W** 1306 1307
1407 **546.9581 1637.8525 1637.8525 -0.01 1 43 0.00057 1 K.SLNNQFASFIDKVR.F** 1408
1666 **538.2752 2149.0717 2149.0705 0.56 1 32 0.013 1 R.THNLEPYFESFINNLRR.R** 1667 1668
1736 **762.7132 2285.1178 2285.1175 0.13 1 59 1.7e-005 1 K.AEAESLYQSKYEELQITAGR.H** 1735
1811 **855.7277 2564.1612 2564.1595 0.64 0 23 0.06 1 R.MSGECAPNVSVSVSTSHTTISGGGSR.G**
1916 **978.1779 2931.5119 2931.5090 0.99 1 32 0.0089 1 R.FLEQQNQVLQTKWELLQQVDTSTR.T**
Proteins matching the same set of peptides:
H6VRF9 **Mass:** 66200 **Score:** 932 **Queries matched:** 34
Keratin 1 OS=Homo sapiens GN=KRT1 PE=3 SV=1
H6VRG0 **Mass:** 66101 **Score:** 932 **Queries matched:** 34
Keratin 1 OS=Homo sapiens GN=KRT1 PE=3 SV=1
H6VRG1 **Mass:** 66257 **Score:** 932 **Queries matched:** 34
Keratin 1 OS=Homo sapiens GN=KRT1 PE=2 SV=1
H6VRG3 **Mass:** 66242 **Score:** 932 **Queries matched:** 34
Keratin 1 OS=Homo sapiens GN=KRT1 PE=3 SV=1
P04264 **Mass:** 66170 **Score:** 932 **Queries matched:** 34
Keratin, type II cytoskeletal 1 OS=Homo sapiens GN=KRT1 PE=1 SV=6
2. Q1KLZ0 **Mass:** 42052 **Score:** 861 **Queries matched:** 47 **emPAI:** 4.65
HCG15971, isoform CRA_a OS=Homo sapiens GN=PS1TP5BP1 PE=2 SV=1
Query Observed Mr(expt) Mr(calc) ppm Miss Score Expect Rank Peptide
434 **322.6899 643.3653 643.3653 0.03 0 39 0.012 1 R.LDLAGR.D**
438 **322.7208 643.4270 643.4268 0.29 0 27 0.017 1 R.GILTLK.Y** 437 439 440
695 **398.2404 794.4663 794.4650 1.57 0 27 0.017 1 K.IIAPPER.K** 693
696 **400.7714 799.5282 799.5280 0.32 1 24 0.031 1 K.RGILTLK.Y** 697
964 **488.7276 975.4406 975.4410 -0.41 0 64 4.6e-006 1 K.AGFAGDDAPR.A**
988 **499.7467 997.4788 997.4790 -0.26 0 28 0.021 1 R.DLTDYLMK.I** 987
1102 **566.7671 1131.5196 1131.5197 -0.03 0 74 4.6e-007 1 R.GYSFTTTAER.E** 1101 1103
1122 **581.3137 1160.6128 1160.6111 1.48 0 61 1.3e-005 1 K.EITALAPSTMK.I** 1121
1133 **586.2881 1170.5617 1170.5638 -1.83 0 60 1.4e-005 1 R.HQGVMVGMGQK.D** 1135
1141 **589.3107 1176.6069 1176.6060 0.80 0 (34) 0.0057 1 K.EITALAPSTMK.I** 1142 1143
1161 **599.8567 1197.6989 1197.6982 0.54 1 41 0.00071 1 R.AVFPSIVGRPR.H**
1397 **541.9517 1622.8332 1622.8338 -0.35 1 45 0.00055 1 R.LDLAGRDLTDYLMK.I** 1395 1396 1398 1399
1402 **543.9470 1628.8193 1628.8158 2.14 1 25 1.4 1 R.GYSFTTTAEREIVR.D** 1401
1411 **547.2833 1638.8279 1638.8287 -0.46 1 (36) 0.0038 1 R.LDLAGRDLTDYLMK.I**
1469 **895.9509 1789.8873 1789.8846 1.49 0 74 7.9e-007 1 K.SYELPDGQVITIGNER.F** 1468
1576 **977.5376 1953.0607 1953.0571 1.84 0 64 2.5e-006 2 R.VAPEEHPVLLTEAPLNPK.A** 1574 1575 1577 1578
1579
1705 **1108.0398 2214.0651 2214.0627 1.09 0 68 2.6e-006 1 K.DLYANTVLSGGTTMYPGIADR.M** 1704 1706
1802 **850.7299 2549.1678 2549.1665 0.51 0 99 2.1e-009 1 K.LCYVALDFEQEMATAASSSSLEK.S**
1959 **1061.8781 3182.6125 3182.6071 1.71 0 41 0.00091 1 R.TTGIVMDSGDGVTHTVPIYEGYALPHAILR.L** 1957
1960 1961
Proteins matching the same set of peptides:
Q8WVW5 **Mass:** 40819 **Score:** 861 **Queries matched:** 47
Putative uncharacterized protein (Fragment) OS=Homo sapiens PE=2 SV=1
Q53G76 **Mass:** 42036 **Score:** 861 **Queries matched:** 47
Beta actin variant (Fragment) OS=Homo sapiens PE=2 SV=1
Q53G99 **Mass:** 42080 **Score:** 861 **Queries matched:** 47
Beta actin variant (Fragment) OS=Homo sapiens PE=2 SV=1
Q53GK6 **Mass:** 42038 **Score:** 861 **Queries matched:** 47
Beta actin variant (Fragment) OS=Homo sapiens PE=2 SV=1
P60709 **Mass:** 42052 **Score:** 861 **Queries matched:** 47
Actin, cytoplasmic 1 OS=Homo sapiens GN=ACTB PE=1 SV=1
P63261 **Mass:** 42108 **Score:** 861 **Queries matched:** 47
Actin, cytoplasmic 2 OS=Homo sapiens GN=ACTG1 PE=1 SV=1
3. P13645 **Mass:** 59020 **Score:** 559 **Queries matched:** 22 **emPAI:** 0.59
Keratin, type I cytoskeletal 10 OS=Homo sapiens GN=KRT10 PE=1 SV=6
Query Observed Mr(expt) Mr(calc) ppm Miss Score Expect Rank Peptide
705 **404.2036 806.3926 806.3923 0.38 0 45 0.00048 1 R.LAADDFR.L** 704 706
979 **497.2539 992.4933 992.4927 0.65 0 37 0.0051 1 K.YENEVALR.Q** 977 978
1011 **516.3031 1030.5917 1030.5910 0.62 0 63 2.8e-006 1 R.VLDELTLTK.A** 1012

1206 **631.8033 1261.5921 1261.5899 1.74 0 88 1.9e-008 1 R.SLLEGEGSSGGGGR.G** 1207
1253 **453.2445 1356.7116 1356.7110 0.44 1 (35) 0.0047 1 R.QSVEADINGLRR.V** 1252
1255 **453.5723 1357.6952 1357.6950 0.17 1 40 0.0026 1 R.QSVEADINGLRR.V** 1256
1265 **691.3281 1380.6416 1380.6408 0.55 0 87 3.8e-008 1 R.ALEESNYELEGK.I** 1263 1264
1288 **478.9292 1433.7658 1433.7626 2.21 1 23 0.092 1 K.IRLENEIQTYR.S**
1434 **854.3906 1706.7667 1706.7649 1.07 0 134 5e-013 1 K.GSLGGGFSSGGFSGGSFSR.G** 1432 1433
1703 **738.0382 2211.0928 2211.0906 0.99 1 43 0.00099 1 K.SKELTTEIDNNIEQISSYK.S**
4. P35527 **Mass:** 62255 **Score:** 525 **Queries matched:** 29 **emPAI:** 0.89
Keratin, type I cytoskeletal 9 OS=Homo sapiens GN=KRT9 PE=1 SV=3
Query Observed Mr(expt) Mr(calc) ppm Miss Score Expect Rank Peptide
871 **449.2103 896.4060 896.4062 -0.22 0 38 0.002 1 R.MTLDDFR.I** 872
1038 **530.7850 1059.5555 1059.5560 -0.49 0 52 0.00018 1 K.TLLDIDNTR.M** 1039 1040
1118 **579.2991 1156.5836 1156.5836 -0.06 0 77 1.8e-007 1 R.QGVDADINGLR.Q** 1117
1119 **579.7917 1157.5689 1157.5677 1.08 0 (22) 0.089 1 R.QGVDADINGLR.Q**
1233 **436.5640 1306.6701 1306.6703 -0.17 1 39 0.002 1 R.IKFEMEQNLR.Q** 1231 1232 1234 1235
1503 **613.3266 1836.9581 1836.9581 -0.02 0 62 6.8e-006 1 R.HGVQELEIELQSQLSK.K** 1501 1502 1504
1521 **617.9814 1850.9224 1850.9196 1.53 1 36 0.0045 1 K.TLNDMRQEYEQLIAK.N** 1519 1520
1529 **623.3133 1866.9182 1866.9145 1.97 1 (29) 0.022 1 K.TLNDMRQEYEQLIAK.N** 1528
1585 **656.0253 1965.0541 1965.0531 0.52 1 74 2e-007 1 R.HGVQELEIELQSQLSKK.A** 1584
1793 **837.3826 2509.1261 2509.1245 0.63 0 65 2.8e-006 1 K.EIETYHNLLEGGQEDFESSGAGK.I** 1792
1970 **1075.0994 3222.2764 3222.2744 0.63 0 29 0.0012 1**
R.GGSGGSHGGGSGFGGESGGSYGGGEEASGSGGGYGGGSGK.S
1979 **1088.8423 3263.5050 3263.5066 -0.46 0 52 5.8e-005 1 K.DIENQYETQITQIEHEVSSSGQEVQSSAK.E** 1978
5. D2JYH4 **Mass:** 42381 **Score:** 511 **Queries matched:** 29 **emPAI:** 2.15
Actin, alpha 2, smooth muscle, aorta OS=Homo sapiens GN=ACTA2 PE=2 SV=1
Query Observed Mr(expt) Mr(calc) ppm Miss Score Expect Rank Peptide
434 322.6899 643.3653 643.3653 0.03 0 39 0.012 1 R.LDLAGR.D
438 322.7208 643.4270 643.4268 0.29 0 27 0.017 1 R.GILTLK.Y 437 439 440
695 398.2404 794.4663 794.4650 1.57 0 27 0.017 1 K.IIAPPER.K 693
696 400.7714 799.5282 799.5280 0.32 1 24 0.031 1 K.RGILTLK.Y 697
964 488.7276 975.4406 975.4410 -0.41 0 64 4.6e-006 1 K.AGFAGDDAPR.A
988 499.7467 997.4788 997.4790 -0.26 0 28 0.021 1 R.DLTDYLMK.I 987
1122 581.3137 1160.6128 1160.6111 1.48 0 61 1.3e-005 1 K.EITALAPSTMK.I 1121
1133 586.2881 1170.5617 1170.5638 -1.83 0 60 1.4e-005 1 R.HQGVMVGMGQK.D 1135
1141 589.3107 1176.6069 1176.6060 0.80 0 (34) 0.0057 1 K.EITALAPSTMK.I 1142 1143
1161 599.8567 1197.6989 1197.6982 0.54 1 41 0.00071 1 R.AVFPSIVGRPR.H
1397 541.9517 1622.8332 1622.8338 -0.35 1 45 0.00055 1 R.LDLAGRDLTDYLMK.I 1395 1396 1398 1399
1411 547.2833 1638.8279 1638.8287 -0.46 1 (36) 0.0038 1 R.LDLAGRDLTDYLMK.I
1469 895.9509 1789.8873 1789.8846 1.49 0 74 7.9e-007 1 K.SYELPDGQVITIGNER.F 1468
1582 **652.6871 1955.0396 1955.0364 1.65 0 20 0.093 1 R.VAPEEHPTLLTEAPLNPK.A**
Proteins matching the same set of peptides:
A8K3K1 **Mass:** 42362 **Score:** 511 **Queries matched:** 29
cDNA FLJ78096, highly similar to Homo sapiens actin, alpha, cardiac muscle (ACTC), mRNA OS=Homo sapiens PE=2 SV=1
P62736 **Mass:** 42381 **Score:** 511 **Queries matched:** 29
Actin, aortic smooth muscle OS=Homo sapiens GN=ACTA2 PE=1 SV=1
P63267 **Mass:** 42249 **Score:** 511 **Queries matched:** 29
Actin, gamma-enteric smooth muscle OS=Homo sapiens GN=ACTG2 PE=1 SV=1
P68032 **Mass:** 42334 **Score:** 511 **Queries matched:** 29
Actin, alpha cardiac muscle 1 OS=Homo sapiens GN=ACTC1 PE=1 SV=1
P68133 **Mass:** 42366 **Score:** 511 **Queries matched:** 29
Actin, alpha skeletal muscle OS=Homo sapiens GN=ACTA1 PE=1 SV=1
6. Q2TSD0 **Mass:** 36197 **Score:** 408 **Queries matched:** 11 **emPAI:** 0.80
Glyceraldehyde-3-phosphate dehydrogenase OS=Homo sapiens PE=2 SV=1
Query Observed Mr(expt) Mr(calc) ppm Miss Score Expect Rank Peptide
692 **398.2132 794.4119 794.4109 1.25 0 31 0.007 1 K.LTGMAFR.V**
703 **403.7110 805.4074 805.4083 -1.06 0 34 0.0036 1 K.VGVNGFGR.I**
1241 **444.2216 1329.6430 1329.6421 0.69 1 23 0.29 1 R.VVDLMAHMASKE.-**
1277 **706.3995 1410.7845 1410.7831 1.05 0 66 1.9e-006 1 R.GALQNIIPASTGAAK.A** 1278
1340 **765.9007 1529.7868 1529.7872 -0.23 0 89 3.9e-008 1 R.VPTANVSVVDLTCR.L**
1458 **882.4067 1762.7989 1762.7951 2.16 0 85 4.3e-008 1 K.LISWYDNEFGYSNR.V** 1457
1500 **917.4656 1832.9166 1832.9124 2.29 0 42 0.0011 1 K.IISNASCTTNCLAPLAK.V**
1815 **649.5958 2594.3540 2594.3527 0.51 0 37 0.0014 1 K.VIHDNFGIVEGLMTTVHAITATQK.T** 1816
Proteins matching the same set of peptides:
V9HVZ4 **Mass:** 36201 **Score:** 408 **Queries matched:** 11
Epididymis secretory sperm binding protein Li 162eP OS=Homo sapiens GN=HEL-S-162eP PE=2 SV=1
P04406 **Mass:** 36201 **Score:** 408 **Queries matched:** 11
Glyceraldehyde-3-phosphate dehydrogenase OS=Homo sapiens GN=GAPDH PE=1 SV=3
7. J3KR22 **Mass:** 33175 **Score:** 406 **Queries matched:** 30 **emPAI:** 1.98
C-type lectin domain family 10 member A OS=Homo sapiens GN=CLEC10A PE=2 SV=1
Query Observed Mr(expt) Mr(calc) ppm Miss Score Expect Rank Peptide
1005 **511.2508 1020.4871 1020.4876 -0.49 0 30 0.017 1 R.EEQNFVQK.Y**

1045 **535.8146 1069.6147 1069.6132 1.41 0 70 1.9e-006 1 R.VQQLVQDLK.K** 1046
1082 **561.8242 1121.6338 1121.6305 2.94 0 50 0.0001 1 K.NAHLVVINSR.E** 1078 1079 1080 1081
1152 **592.3168 1182.6190 1182.6179 0.89 0 51 5.1e-005 1 R.QAVHSEMLLR.V** 1148 1149 1150 1151
1167 **599.8619 1197.7092 1197.7081 0.89 1 71 6.6e-007 1 R.VQQLVQDLKK.L** 1165 1166 1168 1169 1170
1172 **400.5456 1198.6151 1198.6128 1.86 0 (29) 0.013 1 R.QAVHSEMLLR.V** 1171
1649 **709.0426 2124.1060 2124.1076 -0.74 1 37 0.0024 1 K.NAHLVVINSRЕEQNFVQK.Y** 1650 1652 1653 1654
1661 **1066.4959 2130.9772 2130.9721 2.40 0 97 3.2e-009 1 K.YLGSAYTWMGLSDPEGAWK.W** 1658 1659 1660
8. P35908 **Mass:** 65678 **Score:** 351 **Queries matched:** 10 **emPAI:** 0.26
Keratin, type II cytoskeletal 2 epidermal OS=Homo sapiens GN=KRT2 PE=1 SV=2
Query Observed Mr(expt) Mr(calc) ppm Miss Score Expect Rank Peptide
751 **416.2512 830.4879 830.4862 2.12 0 20 0.25 1 R.SLVGLGGTK.S**
960 487.2694 972.5242 972.5240 0.28 0 59 5.3e-005 1 K.IEISELNR.V 959
1020 **519.2677 1036.5209 1036.5189 1.90 0 65 9.2e-006 1 R.YLDGLTAER.T**
1066 **554.2765 1106.5384 1106.5356 2.57 0 46 0.00067 1 K.AQYEEIAQR.S**
1197 **627.8084 1253.6023 1253.6001 1.79 0 90 1.8e-008 1 R.GFSSGSAVVSGGSR.R** 1196
1308 738.3981 1474.7816 1474.7780 2.48 0 71 1.2e-006 1 R.FLEQQNQVLQTK.W 1306 1307
9. P04259 **Mass:** 60315 **Score:** 259 **Queries matched:** 6 **emPAI:** 0.22
Keratin, type II cytoskeletal 6B OS=Homo sapiens GN=KRT6B PE=1 SV=5
Query Observed Mr(expt) Mr(calc) ppm Miss Score Expect Rank Peptide
1066 554.2765 1106.5384 1106.5356 2.57 0 46 0.00067 1 K.AQYEEIAQR.S
1144 590.3038 1178.5931 1178.5931 -0.02 0 84 8.4e-008 1 K.YEELQITAGR.H 1145 1146
1376 **799.8839 1597.7533 1597.7519 0.90 0 83 5.7e-008 1 R.ISIGGGSCAISGGYGSR.A**
1420 **556.5945 1666.7616 1666.7594 1.30 1 45 0.0016 1 R.SRGSGGLGGACGGAGFGSR.S**
10. B4DE59 **Mass:** 62862 **Score:** 239 **Queries matched:** 8 **emPAI:** 0.21
cDNA FLJ60424, highly similar to Junction plakoglobin OS=Homo sapiens PE=2 SV=1
Query Observed Mr(expt) Mr(calc) ppm Miss Score Expect Rank Peptide
705 404.2036 806.3926 806.3923 0.38 0 45 0.00048 1 R.LAADDFR.T 704 706
1010 **515.3009 1028.5873 1028.5866 0.67 0 49 0.00034 1 R.VLDELTLAR.T**
1026 **521.3071 1040.5996 1040.5978 1.68 0 63 5.7e-006 1 R.IVLQIDNAR.L** 1024 1025
1272 **695.3479 1388.6812 1388.6783 2.06 0 82 9.3e-008 1 K.AALEDTLAETEAR.F**
Proteins matching the same set of peptides:
P08727 **Mass:** 44079 **Score:** 239 **Queries matched:** 8
Keratin, type I cytoskeletal 19 OS=Homo sapiens GN=KRT19 PE=1 SV=4
11. P02533 **Mass:** 51872 **Score:** 223 **Queries matched:** 8 **emPAI:** 0.27
Keratin, type I cytoskeletal 14 OS=Homo sapiens GN=KRT14 PE=1 SV=4
Query Observed Mr(expt) Mr(calc) ppm Miss Score Expect Rank Peptide
705 404.2036 806.3926 806.3923 0.38 0 45 0.00048 1 R.LAADDFR.T 704 706
1010 515.3009 1028.5873 1028.5866 0.67 0 49 0.00034 1 R.VLDELTLAR.A
1064 **553.7847 1105.5549 1105.5550 -0.04 0 62 1.1e-005 1 R.ISSVLAGGSCR.A** 1065
1280 **713.3518 1424.6890 1424.6896 -0.41 0 68 2.6e-006 1 R.APSTYGGGLSVSSSR.F** 1281
12. P08779 **Mass:** 51578 **Score:** 214 **Queries matched:** 7 **emPAI:** 0.27
Keratin, type I cytoskeletal 16 OS=Homo sapiens GN=KRT16 PE=1 SV=4
Query Observed Mr(expt) Mr(calc) ppm Miss Score Expect Rank Peptide
705 404.2036 806.3926 806.3923 0.38 0 45 0.00048 1 R.LAADDFR.T 704 706
1010 515.3009 1028.5873 1028.5866 0.67 0 49 0.00034 1 R.VLDELTLAR.T
1064 553.7847 1105.5549 1105.5550 -0.04 0 62 1.1e-005 1 R.ISSVLAGGSCR.A 1065
1243 **669.8368 1337.6590 1337.6575 1.10 0 58 2.1e-005 1 R.APSTYGGGLSVSSR.F**
13. Q1KSF8 **Mass:** 23896 **Score:** 151 **Queries matched:** 4 **emPAI:** 0.46
XTP3TPA-transactivated protein 1 OS=Homo sapiens GN=XTP3TPATP1 PE=2 SV=1
Query Observed Mr(expt) Mr(calc) ppm Miss Score Expect Rank Peptide
1109 **572.3206 1142.6266 1142.6270 -0.41 0 53 0.00016 1 K.LAVNMVPFPR.L** 1110
1238 **660.3553 1318.6960 1318.6955 0.37 0 43 0.00065 1 R.IMNTFSVVPSPK.V**
1388 **540.9493 1619.8261 1619.8283 -1.31 0 55 6.4e-005 1 R.LHFFMPGFAPLTSR.G**
Proteins matching the same set of peptides:
B3KML9 **Mass:** 44916 **Score:** 151 **Queries matched:** 4
cDNA FLJ11352 fis, clone HEMBA1000020, highly similar to Tubulin beta-2C chain OS=Homo sapiens PE=2 SV=1
B4DE77 **Mass:** 46211 **Score:** 151 **Queries matched:** 4
cDNA FLJ55189, highly similar to Tubulin beta-4 chain OS=Homo sapiens PE=2 SV=1
B4DQN9 **Mass:** 42096 **Score:** 151 **Queries matched:** 4
cDNA FLJ50617, highly similar to Tubulin beta-7 chain OS=Homo sapiens PE=2 SV=1
B4DY90 **Mass:** 52414 **Score:** 151 **Queries matched:** 4
cDNA FLJ56903, highly similar to Tubulin beta-7 chain OS=Homo sapiens PE=2 SV=1
B4E052 **Mass:** 40000 **Score:** 151 **Queries matched:** 4
cDNA FLJ52378, highly similar to Tubulin beta-7 chain OS=Homo sapiens PE=2 SV=1
Q5JP53 **Mass:** 48135 **Score:** 151 **Queries matched:** 4
Tubulin beta chain OS=Homo sapiens GN=TUBB PE=4 SV=1
Q5ST81 **Mass:** 42114 **Score:** 151 **Queries matched:** 4
Tubulin beta chain OS=Homo sapiens GN=TUBB PE=3 SV=1
Q5SU16 **Mass:** 50095 **Score:** 151 **Queries matched:** 4
Beta 5-tubulin OS=Homo sapiens GN=TUBB PE=2 SV=1
Q6LC01 **Mass:** 49362 **Score:** 151 **Queries matched:** 4
MRNA encoding beta-tubulin. (from clone D-beta-1) (Fragment) OS=Homo sapiens PE=2 SV=1

B7ZAF0 **Mass:** 46936 **Score:** 151 **Queries matched:** 4
cDNA, FLJ79164, highly similar to Tubulin beta-7 chain OS=Homo sapiens PE=2 SV=1
Q8IWP6 **Mass:** 50177 **Score:** 151 **Queries matched:** 4
Class IVb beta tubulin OS=Homo sapiens PE=2 SV=1
Q8IZ29 **Mass:** 50264 **Score:** 151 **Queries matched:** 4
Tubulin, beta 2C OS=Homo sapiens GN=TUBB2C PE=2 SV=1
Q8N6N5 **Mass:** 50232 **Score:** 151 **Queries matched:** 4
Tubulin, beta 2C OS=Homo sapiens GN=TUBB2C PE=2 SV=1
Q9BUU9 **Mass:** 38690 **Score:** 151 **Queries matched:** 4
TUBB protein (Fragment) OS=Homo sapiens GN=TUBB PE=2 SV=1
P04350 **Mass:** 50010 **Score:** 151 **Queries matched:** 4
Tubulin beta-4A chain OS=Homo sapiens GN=TUBB4A PE=1 SV=2
P07437 **Mass:** 50095 **Score:** 151 **Queries matched:** 4
Tubulin beta chain OS=Homo sapiens GN=TUBB PE=1 SV=2
P68371 **Mass:** 50255 **Score:** 151 **Queries matched:** 4
Tubulin beta-4B chain OS=Homo sapiens GN=TUBB4B PE=1 SV=1
14. B4DNE0 **Mass:** 42911 **Score:** 128 **Queries matched:** 2 **emPAI:** 0.15
cDNA FLJ52573, highly similar to Elongation factor 1-alpha 1 OS=Homo sapiens PE=2 SV=1
Query Observed Mr(expt) Mr(calc) ppm Miss Score Expect Rank Peptide
1009 **513.3086 1024.6026 1024.6030 -0.37 0 98 1e-009 1 K.IGGIGTVPVGR.V**
1369 **530.2985 1587.8737 1587.8733 0.25 0 30 0.0068 1 K.THINIVVIGHVDSGK.S**
Proteins matching the same set of peptides:
Q5VTE0 **Mass:** 50495 **Score:** 128 **Queries matched:** 2
Putative elongation factor 1-alpha-like 3 OS=Homo sapiens GN=EEF1A1P5 PE=5 SV=1
Q6IPN6 **Mass:** 50433 **Score:** 128 **Queries matched:** 2
Elongation factor 1-alpha OS=Homo sapiens GN=EEF1A1 PE=2 SV=1
Q6IPS9 **Mass:** 50451 **Score:** 128 **Queries matched:** 2
Elongation factor 1-alpha OS=Homo sapiens GN=EEF1A1 PE=2 SV=1
Q6IPT9 **Mass:** 50438 **Score:** 128 **Queries matched:** 2
Elongation factor 1-alpha OS=Homo sapiens GN=EEF1A1 PE=2 SV=1
Q6IQ15 **Mass:** 48181 **Score:** 128 **Queries matched:** 2
EEF1A1 protein OS=Homo sapiens GN=EEF1A1 PE=2 SV=1
A8K9C4 **Mass:** 50511 **Score:** 128 **Queries matched:** 2
Elongation factor 1-alpha OS=Homo sapiens PE=2 SV=1
Q53G85 **Mass:** 50423 **Score:** 128 **Queries matched:** 2
Elongation factor 1-alpha (Fragment) OS=Homo sapiens PE=2 SV=1
Q53GA1 **Mass:** 50421 **Score:** 128 **Queries matched:** 2
Elongation factor 1-alpha (Fragment) OS=Homo sapiens PE=2 SV=1
Q53GE9 **Mass:** 50421 **Score:** 128 **Queries matched:** 2
Elongation factor 1-alpha (Fragment) OS=Homo sapiens PE=2 SV=1
Q53HM9 **Mass:** 50452 **Score:** 128 **Queries matched:** 2
Elongation factor 1-alpha (Fragment) OS=Homo sapiens PE=2 SV=1
Q53HQ7 **Mass:** 50509 **Score:** 128 **Queries matched:** 2
Elongation factor 1-alpha (Fragment) OS=Homo sapiens PE=2 SV=1
Q53HR5 **Mass:** 50435 **Score:** 128 **Queries matched:** 2
Elongation factor 1-alpha (Fragment) OS=Homo sapiens PE=2 SV=1
Q05639 **Mass:** 50780 **Score:** 128 **Queries matched:** 2
Elongation factor 1-alpha 2 OS=Homo sapiens GN=EEF1A2 PE=1 SV=1
P68104 **Mass:** 50451 **Score:** 128 **Queries matched:** 2
Elongation factor 1-alpha 1 OS=Homo sapiens GN=EEF1A1 PE=1 SV=1
15. Q7L4M3 **Mass:** 30802 **Score:** 98 **Queries matched:** 2 **emPAI:** 0.22
KRT8 protein OS=Homo sapiens PE=2 SV=1
Query Observed Mr(expt) Mr(calc) ppm Miss Score Expect Rank Peptide
1215 639.3587 1276.7027 1276.7027 0.06 0 74 5e-007 1 K.LALDIEIATYR.K
1245 **447.9210 1340.7410 1340.7412 -0.12 1 24 0.12 1 R.LQAEIEGLKGQR.A**
Proteins matching the same set of peptides:
Q969I0 **Mass:** 41083 **Score:** 98 **Queries matched:** 2
KRT8 protein (Fragment) OS=Homo sapiens GN=KRT8 PE=2 SV=2
P05787 **Mass:** 53671 **Score:** 98 **Queries matched:** 2
Keratin, type II cytoskeletal 8 OS=Homo sapiens GN=KRT8 PE=1 SV=7
16. H0YAS8 **Mass:** 16224 **Score:** 92 **Queries matched:** 3 **emPAI:** 0.44
Clusterin beta chain (Fragment) OS=Homo sapiens GN=CLU PE=4 SV=1
Query Observed Mr(expt) Mr(calc) ppm Miss Score Expect Rank Peptide
1221 **644.8236 1287.6327 1287.6306 1.64 0 59 2.5e-005 1 R.ELDESLQVAER.L** 1220
1747 **772.0651 2313.1736 2313.1700 1.52 0 33 0.028 1 R.VTTVASHTSDSDVPSGVTEVVVK.L**
Proteins matching the same set of peptides:
H0YC35 **Mass:** 33794 **Score:** 92 **Queries matched:** 3
Clusterin (Fragment) OS=Homo sapiens GN=CLU PE=3 SV=1
B4DW11 **Mass:** 25952 **Score:** 92 **Queries matched:** 3
cDNA FLJ57622, highly similar to Clusterin OS=Homo sapiens PE=2 SV=1
P10909 **Mass:** 53031 **Score:** 92 **Queries matched:** 3
Clusterin OS=Homo sapiens GN=CLU PE=1 SV=1
17. H0YKS4 **Mass:** 19632 **Score:** 67 **Queries matched:** 2 **emPAI:** 0.35

Annexin (Fragment) OS=Homo sapiens GN=ANXA2 PE=3 SV=1
Query Observed Mr(expt) Mr(calc) ppm Miss Score Expect Rank Peptide
1179 **611.8025 1221.5904 1221.5877 2.17 0 30 0.48 1 K.TPAQYDASELK.A**
1195 **622.8156 1243.6167 1243.6156 0.85 0 37 0.0035 1 R.TNQELQEINR.V**
Proteins matching the same set of peptides:
H0YM50 **Mass:** 28582 **Score:** 67 **Queries matched:** 2
Annexin (Fragment) OS=Homo sapiens GN=ANXA2 PE=3 SV=1
H0YMD0 **Mass:** 25440 **Score:** 67 **Queries matched:** 2
Annexin (Fragment) OS=Homo sapiens GN=ANXA2 PE=2 SV=1
H0YMM1 **Mass:** 16563 **Score:** 67 **Queries matched:** 2
Annexin (Fragment) OS=Homo sapiens GN=ANXA2 PE=2 SV=1
H0YMU9 **Mass:** 25667 **Score:** 67 **Queries matched:** 2
Annexin OS=Homo sapiens GN=ANXA2 PE=2 SV=1
H0YN42 **Mass:** 28921 **Score:** 67 **Queries matched:** 2
Annexin (Fragment) OS=Homo sapiens GN=ANXA2 PE=2 SV=1
H0YNP5 **Mass:** 19829 **Score:** 67 **Queries matched:** 2
Annexin (Fragment) OS=Homo sapiens GN=ANXA2 PE=2 SV=1
B4DNH8 **Mass:** 21827 **Score:** 67 **Queries matched:** 2
Annexin OS=Homo sapiens PE=2 SV=1
A6NMY6 **Mass:** 38806 **Score:** 67 **Queries matched:** 2
Putative annexin A2-like protein OS=Homo sapiens GN=ANXA2P2 PE=5 SV=2
V9HW65 **Mass:** 38780 **Score:** 67 **Queries matched:** 2
Annexin OS=Homo sapiens GN=HEL-S-270 PE=2 SV=1
P07355 **Mass:** 38808 **Score:** 67 **Queries matched:** 2

Annexin A2 OS=Homo sapiens GN=ANXA2 PE=1 SV=2
18. A6NE09 **Mass:** 33002 **Score:** 65 **Queries matched:** 1 **emPAI:** 0.10
40S ribosomal protein SA OS=Homo sapiens GN=RPSAP58 PE=3 SV=1
Query Observed Mr(expt) Mr(calc) ppm Miss Score Expect Rank Peptide
1174 **602.3288 1202.6430 1202.6408 1.86 0 65 7.8e-006 1 K.FAAATGATPIAGR.F**
Proteins matching the same set of peptides:
C9J9K3 **Mass:** 29601 **Score:** 65 **Queries matched:** 1
40S ribosomal protein SA (Fragment) OS=Homo sapiens GN=RPSA PE=3 SV=1
Q96RS2 **Mass:** 33089 **Score:** 65 **Queries matched:** 1
40S ribosomal protein SA OS=Homo sapiens GN=LAMR1P15 PE=2 SV=1
P08865 **Mass:** 32947 **Score:** 65 **Queries matched:** 1
40S ribosomal protein SA OS=Homo sapiens GN=RPSA PE=1 SV=4
19. F8VV57 **Mass:** 12207 **Score:** 58 **Queries matched:** 2 **emPAI:** 0.27
Keratin, type II cytoskeletal 5 (Fragment) OS=Homo sapiens GN=KRT5 PE=2 SV=1
Query Observed Mr(expt) Mr(calc) ppm Miss Score Expect Rank Peptide
1068 **556.2908 1110.5670 1110.5669 0.01 0 58 2.8e-005 1 R.ISISTSGGSFR.N** 1069
Proteins matching the same set of peptides:
P13647 **Mass:** 62568 **Score:** 58 **Queries matched:** 2
Keratin, type II cytoskeletal 5 OS=Homo sapiens GN=KRT5 PE=1 SV=3
20. Q0Z944 **Mass:** 11554 **Score:** 55 **Queries matched:** 1 **emPAI:** 0.28
Beta globin (Fragment) OS=Homo sapiens GN=HBB PE=2 SV=1
Query Observed Mr(expt) Mr(calc) ppm Miss Score Expect Rank Peptide
1237 **657.8374 1313.6603 1313.6575 2.11 0 55 5.4e-005 1 K.VNVDEVGGEALGR.L**
Proteins matching the same set of peptides:
B3VL05 **Mass:** 11536 **Score:** 55 **Queries matched:** 1
Beta globin (Fragment) OS=Homo sapiens PE=3 SV=1
B3VL12 **Mass:** 4263 **Score:** 55 **Queries matched:** 1
Truncated beta globin OS=Homo sapiens PE=2 SV=1
B3VL17 **Mass:** 11534 **Score:** 55 **Queries matched:** 1
Beta globin (Fragment) OS=Homo sapiens PE=3 SV=1
B3VL31 **Mass:** 11550 **Score:** 55 **Queries matched:** 1
Beta globin (Fragment) OS=Homo sapiens PE=3 SV=1
B3VL86 **Mass:** 15175 **Score:** 55 **Queries matched:** 1
Mutant beta-globin OS=Homo sapiens GN=HBB PE=3 SV=1
Q3LR79 **Mass:** 11532 **Score:** 55 **Queries matched:** 1
Hemoglobin beta (Fragment) OS=Homo sapiens GN=HBB PE=3 SV=1
Q3Y9I8 **Mass:** 11013 **Score:** 55 **Queries matched:** 1
Hemoglobin beta (Fragment) OS=Homo sapiens GN=HBB PE=3 SV=1
Q4TWB7 **Mass:** 11537 **Score:** 55 **Queries matched:** 1
Beta globin (Fragment) OS=Homo sapiens GN=HBB PE=3 SV=1
Q4TZM4 **Mass:** 11073 **Score:** 55 **Queries matched:** 1
Hemoglobin beta chain (Fragment) OS=Homo sapiens GN=HBB PE=3 SV=1
B5ANL9 **Mass:** 9464 **Score:** 55 **Queries matched:** 1
Beta globin chain (Fragment) OS=Homo sapiens GN=HBB PE=2 SV=1
D5FZW3 **Mass:** 4547 **Score:** 55 **Queries matched:** 1
Beta globin (Fragment) OS=Homo sapiens PE=2 SV=1
Q6V0K9 **Mass:** 11524 **Score:** 55 **Queries matched:** 1
Mutant hemoglobin beta chain (Fragment) OS=Homo sapiens GN=HBB PE=3 SV=1

J7LKS8 **Mass:** 3518 **Score:** 55 **Queries matched:** 1
Hemoglobin beta (Fragment) OS=Homo sapiens GN=HBB PE=2 SV=1
C8C504 **Mass:** 16101 **Score:** 55 **Queries matched:** 1
Beta-globin OS=Homo sapiens GN=HBB PE=3 SV=1
F8W6P5 **Mass:** 9664 **Score:** 55 **Queries matched:** 1
LVV-hemorphin-7 (Fragment) OS=Homo sapiens GN=HBB PE=2 SV=1
Q8IUL9 **Mass:** 11598 **Score:** 55 **Queries matched:** 1
Hemoglobin beta chain variant Hb.Sinai-Bel Air (Fragment) OS=Homo sapiens GN=HBB PE=3 SV=1
Q8IZI0 **Mass:** 11555 **Score:** 55 **Queries matched:** 1
Hemoglobin beta chain variant Hb-I_Toulouse (Fragment) OS=Homo sapiens GN=HBB PE=3 SV=1
A9YUX2 **Mass:** 7051 **Score:** 55 **Queries matched:** 1
Truncated beta-globin OS=Homo sapiens GN=HBB PE=3 SV=1
D9YZU5 **Mass:** 16102 **Score:** 55 **Queries matched:** 1
Hemoglobin, beta OS=Homo sapiens GN=HBB PE=2 SV=1
E9M263 **Mass:** 6248 **Score:** 55 **Queries matched:** 1
Hemoglobin beta chain (Fragment) OS=Homo sapiens GN=HBB PE=2 SV=1
E9NGZ5 **Mass:** 11544 **Score:** 55 **Queries matched:** 1
Hemoglobin beta globin chain (Fragment) OS=Homo sapiens GN=HBB PE=3 SV=1
Q9BWU5 **Mass:** 11551 **Score:** 55 **Queries matched:** 1
Mutant hemoglobin beta chain (Fragment) OS=Homo sapiens GN=HBB PE=3 SV=1
Q9BWV6 **Mass:** 12283 **Score:** 55 **Queries matched:** 1
Mutant beta globin OS=Homo sapiens GN=HBB PE=3 SV=1
Q9BXA2 **Mass:** 6459 **Score:** 55 **Queries matched:** 1
Beta-globin (Fragment) OS=Homo sapiens PE=2 SV=1
Q9GZL9 **Mass:** 12635 **Score:** 55 **Queries matched:** 1
Beta-globin (Fragment) OS=Homo sapiens GN=HBB PE=2 SV=1
Q9H1I6 **Mass:** 3292 **Score:** 55 **Queries matched:** 1
Mutant beta-globin (Fragment) OS=Homo sapiens GN=HBB PE=2 SV=1
Q9UBV6 **Mass:** 6656 **Score:** 55 **Queries matched:** 1
Beta-globin (Fragment) OS=Homo sapiens GN=HBB PE=3 SV=1
Q9UP81 **Mass:** 9740 **Score:** 55 **Queries matched:** 1
Mutant beta-globin OS=Homo sapiens GN=HBB PE=3 SV=1
Q52MT0 **Mass:** 11526 **Score:** 55 **Queries matched:** 1
Beta globin (Fragment) OS=Homo sapiens GN=HBB PE=3 SV=1
Q14473 **Mass:** 19204 **Score:** 55 **Queries matched:** 1
Beta-globin gene from a thalassemia patient, OS=Homo sapiens PE=3 SV=1
Q14484 **Mass:** 6686 **Score:** 55 **Queries matched:** 1
Beta-globin (Fragment) OS=Homo sapiens GN=HBB PE=2 SV=1
P68871 **Mass:** 16102 **Score:** 55 **Queries matched:** 1
Hemoglobin subunit beta OS=Homo sapiens GN=HBB PE=1 SV=2
O95408 **Mass:** 6262 **Score:** 55 **Queries matched:** 1
Beta globin (Fragment) OS=Homo sapiens GN=HBB PE=3 SV=1
21. H7C4T5 **Mass:** 36596 **Score:** 48 **Queries matched:** 3 **emPAI:** 0.09
Muscleblind-like protein 1 (Fragment) OS=Homo sapiens GN=MBNL1 PE=4 SV=1
Query Observed Mr(expt) Mr(calc) ppm Miss Score Expect Rank Peptide
828 **434.7670 867.5195 867.5178 1.97 0 21 0.088 1 -.PVSVTPIR.D**
832 **435.7737 869.5328 869.5335 -0.74 0 27 0.019 1 -.VVSVTPIR.D** 841
22. A0N4V7 **Mass:** 2269 **Score:** 44 **Queries matched:** 17 **emPAI:** 6.57
HCG2039797 (Fragment) OS=Homo sapiens GN=Tcr-alpha PE=4 SV=1
Query Observed Mr(expt) Mr(calc) ppm Miss Score Expect Rank Peptide
774 **421.7588 841.5030 841.5022 1.01 1 44 0.00078 1 K.GITLSVRP.-** 766 767 768 769 770 771 775 776 777 779 780 781 782 783 784 785
23. Q9NSB2 **Mass:** 65942 **Score:** 41 **Queries matched:** 1 **emPAI:** 0.05
Keratin, type II cuticular Hb4 OS=Homo sapiens GN=KRT84 PE=2 SV=2
Query Observed Mr(expt) Mr(calc) ppm Miss Score Expect Rank Peptide
1257 **679.8672 1357.7199 1357.7202 -0.15 0 41 0.0013 1 R.VAPATGDLLSTGTR.S**
24. Q1ZYQ1 **Mass:** 50612 **Score:** 39 **Queries matched:** 2 **emPAI:** 0.06
Tubulin, alpha 2 OS=Homo sapiens GN=TUBA2 PE=2 SV=1
Query Observed Mr(expt) Mr(calc) ppm Miss Score Expect Rank Peptide
1775 **604.5575 2414.2011 2414.1978 1.34 1 39 0.0019 1 R.QLFHPEQLITGKEDAANNYAR.G** 1774
Proteins matching the same set of peptides:
B3KPS3 **Mass:** 46725 **Score:** 39 **Queries matched:** 2
cDNA FLJ32131 fis, clone PEBLM2000267, highly similar to Tubulin alpha-ubiquitous chain OS=Homo sapiens PE=2 SV=1
B3KT06 **Mass:** 46825 **Score:** 39 **Queries matched:** 2
cDNA FLJ37398 fis, clone BRAMY2027467, highly similar to Tubulin alpha-ubiquitous chain OS=Homo sapiens PE=2 SV=1
F5H5D3 **Mass:** 58606 **Score:** 39 **Queries matched:** 2
Tubulin alpha-1C chain OS=Homo sapiens GN=TUBA1C PE=2 SV=1
Q6QMJ5 **Mass:** 10870 **Score:** 39 **Queries matched:** 2
Tubulin alpha-1 (Fragment) OS=Homo sapiens GN=TUBA1 PE=2 SV=1
B7Z1K5 **Mass:** 58636 **Score:** 39 **Queries matched:** 2
cDNA FLJ55956, highly similar to Tubulin alpha-6 chain OS=Homo sapiens PE=2 SV=1

Q7Z3M3 **Mass:** 54619 **Score:** 39 **Queries matched:** 2
Putative uncharacterized protein DKFZp686L04275 (Fragment) OS=Homo sapiens GN=DKFZp686L04275 PE=2 SV=1
A8JZY9 **Mass:** 50788 **Score:** 39 **Queries matched:** 2
cDNA FLJ78587 OS=Homo sapiens PE=2 SV=1
A8MUB1 **Mass:** 48982 **Score:** 39 **Queries matched:** 2
Tubulin alpha-4A chain OS=Homo sapiens GN=TUBA4A PE=2 SV=1
F8VQQ4 **Mass:** 24377 **Score:** 39 **Queries matched:** 2
Tubulin alpha-1A chain (Fragment) OS=Homo sapiens GN=TUBA1A PE=2 SV=1
F8VRZ4 **Mass:** 12269 **Score:** 39 **Queries matched:** 2
Tubulin alpha-1A chain (Fragment) OS=Homo sapiens GN=TUBA1A PE=2 SV=1
F8VS66 **Mass:** 14563 **Score:** 39 **Queries matched:** 2
Tubulin alpha-1C chain OS=Homo sapiens GN=TUBA1C PE=2 SV=1
F8VVB9 **Mass:** 27877 **Score:** 39 **Queries matched:** 2
Tubulin alpha-1B chain (Fragment) OS=Homo sapiens GN=TUBA1B PE=2 SV=1
F8VWV9 **Mass:** 8938 **Score:** 39 **Queries matched:** 2
Tubulin alpha-1B chain (Fragment) OS=Homo sapiens GN=TUBA1B PE=2 SV=1
F8VX09 **Mass:** 8283 **Score:** 39 **Queries matched:** 2
Tubulin alpha-1B chain (Fragment) OS=Homo sapiens GN=TUBA1B PE=2 SV=1
Q8N532 **Mass:** 37309 **Score:** 39 **Queries matched:** 2
TUBA1C protein OS=Homo sapiens GN=TUBA1C PE=2 SV=1
Q8WU19 **Mass:** 37707 **Score:** 39 **Queries matched:** 2
TUBA1B protein OS=Homo sapiens GN=TUBA1B PE=2 SV=1
C9J2C0 **Mass:** 52750 **Score:** 39 **Queries matched:** 2
Tubulin alpha-8 chain (Fragment) OS=Homo sapiens GN=TUBA8 PE=3 SV=1
C9JDL2 **Mass:** 13455 **Score:** 39 **Queries matched:** 2
Tubulin alpha-4A chain (Fragment) OS=Homo sapiens GN=TUBA4A PE=2 SV=1
C9JEV8 **Mass:** 17093 **Score:** 39 **Queries matched:** 2
Tubulin alpha-4A chain (Fragment) OS=Homo sapiens GN=TUBA4A PE=2 SV=1
C9JJQ8 **Mass:** 21949 **Score:** 39 **Queries matched:** 2
Tubulin alpha-4A chain (Fragment) OS=Homo sapiens GN=TUBA4A PE=2 SV=1
C9JQ00 **Mass:** 20024 **Score:** 39 **Queries matched:** 2
Tubulin alpha-4A chain (Fragment) OS=Homo sapiens GN=TUBA4A PE=2 SV=1
C9K0S6 **Mass:** 5331 **Score:** 39 **Queries matched:** 2
Tubulin alpha-8 chain (Fragment) OS=Homo sapiens GN=TUBA8 PE=4 SV=1
Q9BQE3 **Mass:** 50548 **Score:** 39 **Queries matched:** 2
Tubulin alpha-1C chain OS=Homo sapiens GN=TUBA1C PE=1 SV=1
Q9NY65 **Mass:** 50746 **Score:** 39 **Queries matched:** 2
Tubulin alpha-8 chain OS=Homo sapiens GN=TUBA8 PE=1 SV=1
Q53GA7 **Mass:** 50476 **Score:** 39 **Queries matched:** 2
Tubulin alpha 6 variant (Fragment) OS=Homo sapiens PE=2 SV=1
Q71U36 **Mass:** 50788 **Score:** 39 **Queries matched:** 2
Tubulin alpha-1A chain OS=Homo sapiens GN=TUBA1A PE=1 SV=1
Q13748 **Mass:** 50612 **Score:** 39 **Queries matched:** 2
Tubulin alpha-3C/D chain OS=Homo sapiens GN=TUBA3C PE=1 SV=3
P68363 **Mass:** 50804 **Score:** 39 **Queries matched:** 2
Tubulin alpha-1B chain OS=Homo sapiens GN=TUBA1B PE=1 SV=1
P68366 **Mass:** 50634 **Score:** 39 **Queries matched:** 2
Tubulin alpha-4A chain OS=Homo sapiens GN=TUBA4A PE=1 SV=1
25. L0R5A1 **Mass:** 11752 **Score:** 39 **Queries matched:** 5 **emPAI:** 0.28
Alternative protein CSF2RB OS=Homo sapiens GN=CSF2RB PE=4 SV=1
Query Observed Mr(expt) Mr(calc) ppm Miss Score Expect Rank Peptide
810 **428.7663 855.5180 855.5178 0.27 0 39 0.0021 1 R.LASIVTPR.N** 805 806 808 809
26. B7Z945 **Mass:** 20378 **Score:** 33 **Queries matched:** 3 **emPAI:** 0.16
Protein LOC100132891 OS=Homo sapiens GN=LOC100132891 PE=2 SV=1
Query Observed Mr(expt) Mr(calc) ppm Miss Score Expect Rank Peptide
1224 **648.3391 1294.6637 1294.6629 0.56 1 33 0.0066 1 R.VGAQVQNPRAPR.K** 1223 1225
27. Q59E90 **Mass:** 113918 **Score:** 25 **Queries matched:** 2 **emPAI:** 0.03
Mannosidase, alpha, class 2B, member 1 variant (Fragment) OS=Homo sapiens PE=2 SV=1
Query Observed Mr(expt) Mr(calc) ppm Miss Score Expect Rank Peptide
1184 **613.8599 1225.7052 1225.7030 1.77 0 25 0.021 1 K.LIQLVNAQQAK.G** 1185

Sample T9 blue box

Database : HumanUniprot uniprot140506 (136615 sequences; 43285968 residues)
Enzyme : Trypsin
Fixed modifications : Carbamidomethyl (C)
Variable modifications : Deamidated (NQ),Oxidation (M)
Mass values : Monoisotopic
Protein Mass : Unrestricted
Peptide Mass Tolerance : ± 3 ppm
Fragment Mass Tolerance: ± 0.6 Da
Max Missed Cleavages : 1
Instrument type : ESI-TRAP
Number of queries : 1647
Protein hits : P49327 Fatty acid synthase OS=Homo sapiens GN=FASN PE=1 SV=3 **H6VRF8** Keratin 1 OS=Homo sapiens GN=KRT1 PE=3 SV=1 **A7Y9J9** Mucin 5AC, oligomeric mucus/gel-forming OS=Homo sapiens GN=MUC5AC PE=4 SV=1 **P13645** Keratin, type I cytoskeletal 10 OS=Homo sapiens GN=KRT10 PE=1 SV=6 **B1AVQ5** Mucin-1 subunit alpha OS=Homo sapiens GN=MUC1 PE=2 SV=1 **P35527** Keratin, type I cytoskeletal 9 OS=Homo sapiens GN=KRT9 PE=1 SV=3 **H0Y4K8** Ugl-Y3 (Fragment) OS=Homo sapiens GN=FN1 PE=2 SV=1 **A0N4V7** HCG2039797 (Fragment) OS=Homo sapiens GN=Tcr-alpha PE=4 SV=1 **L0R5A1** Alternative protein CSF2RB OS=Homo sapiens GN=CSF2RB PE=4 SV=1 **H7C4T5** Muscleblind-like protein 1 (Fragment) OS=Homo sapiens GN=MBNL1 PE=4 SV=1

Select Summary Report

Significance threshold p< Max. number of hits
Standard scoring MudPIT scoring Ions score or expect cut-off Show sub-sets
Show pop-ups Suppress pop-ups Sort unassigned Decreasing Score Require bold red

1. P49327 **Mass:** 275877 **Score:** 395 **Queries matched:** 13 **emPAI:** 0.11
Fatty acid synthase OS=Homo sapiens GN=FASN PE=1 SV=3

Query	Observed	Mr(expt)	Mr(calc)	ppm	Miss	Score	Expect	Rank	Peptide	
705	481.2706	960.5267	960.5240	2.84	0	44	0.0011	1	K.TGTVSLEVR.L	
951	704.8665	1407.7184	1407.7180	0.24	0	41	0.0013	1	R.LSIPTYGLQCTR.A	
953	713.8916	1425.7686	1425.7650	2.56	0	65	4.1e-006	1	R.SLLVNPEGPTLMR.L	952
1065	889.5010	1776.9874	1776.9873	0.07	0	82	2e-008	1	K.LPEDPLLSGLLDSPALK.A	
1111	630.0052	1886.9938	1886.9963	-1.32	0	50	0.00013	1	R.VTVAGGVHISGLHTESAPR.R	
1195	731.4058	2191.1954	2191.2001	-2.12	0	22	0.022	1	R.IPGLLSPHPLLQLSYTATDR.H	
1259	824.7715	2471.2926	2471.2961	-1.44	0	29	0.0089	1	R.LHLSGIDANPNALFPPVEFPAPR.G	
1274	848.0757	2541.2054	2541.2023	1.19	0	32	0.012	1	R.SLYQSAGVAPESFEYIEAHGTGTK.V	1275 1276
1301	667.5919	2666.3384	2666.3340	1.66	1	30	0.0092	1	R.HFLLEEDKPEEPTAHAFVSTLTR.G	1300

2. H6VRF8 **Mass:** 66184 **Score:** 376 **Queries matched:** 10 **emPAI:** 0.38
Keratin 1 OS=Homo sapiens GN=KRT1 PE=3 SV=1

Query	Observed	Mr(expt)	Mr(calc)	ppm	Miss	Score	Expect	Rank	Peptide	
637	437.7531	873.4917	873.4920	-0.36	0	39	0.0053	1	R.SLVNLGGSK.S	
774	517.2608	1032.5071	1032.5087	-1.55	0	27	0.051	1	R.TLLEGEESR.M	
799	533.2653	1064.5161	1064.5138	2.13	0	56	5.5e-005	1	K.AQYEDIAQK.S	798

880 **633.3235 1264.6324 1264.6299 1.93 0 57 4.4e-005 1 R.TNAENEFVTIK.K**
903 **651.8626 1301.7106 1301.7078 2.15 0 108 3e-010 1 R.SLDLDSIIAEVK.A** 901 902
974 **738.3951 1474.7757 1474.7780 -1.56 0 60 1.3e-005 1 R.FLEQQNQVLQTK.W**
1180 **717.3628 2149.0666 2149.0705 -1.80 1 28 0.035 1 R.THNLEPYFESFINNLRR.R**
Proteins matching the same set of peptides:
H6VRG0 **Mass:** 66101 **Score:** 376 **Queries matched:** 10
Keratin 1 OS=Homo sapiens GN=KRT1 PE=3 SV=1
H6VRG1 **Mass:** 66257 **Score:** 376 **Queries matched:** 10
Keratin 1 OS=Homo sapiens GN=KRT1 PE=2 SV=1
H6VRG3 **Mass:** 66242 **Score:** 376 **Queries matched:** 10
Keratin 1 OS=Homo sapiens GN=KRT1 PE=3 SV=1
P04264 **Mass:** 66170 **Score:** 376 **Queries matched:** 10
Keratin, type II cytoskeletal 1 OS=Homo sapiens GN=KRT1 PE=1 SV=6
3. A7Y9J9 **Mass:** 666520 **Score:** 215 **Queries matched:** 6 **emPAI:** 0.01
Mucin 5AC, oligomeric mucus/gel-forming OS=Homo sapiens GN=MUC5AC PE=4 SV=1
Query Observed Mr(expt) Mr(calc) ppm Miss Score Expect Rank Peptide
777 **518.7977 1035.5809 1035.5825 -1.53 0 37 0.0033 1 R.AQAQPGVPLR.E**
995 **754.8740 1507.7334 1507.7307 1.77 0 105 5.7e-010 1 R.SVVGDALEFGNSWK.L** 993 994
1040 **844.3973 1686.7800 1686.7818 -1.05 0 80 1.1e-007 1 R.AAGGAVCEQPLGLECR.A** 1041
Proteins matching the same set of peptides:
E9PBJ0 **Mass:** 611987 **Score:** 215 **Queries matched:** 6
Mucin-5B OS=Homo sapiens GN=MUC5B PE=2 SV=1
Q9HC84 **Mass:** 611584 **Score:** 215 **Queries matched:** 6
Mucin-5B OS=Homo sapiens GN=MUC5B PE=1 SV=3
4. P13645 **Mass:** 59020 **Score:** 172 **Queries matched:** 4 **emPAI:** 0.17
Keratin, type I cytoskeletal 10 OS=Homo sapiens GN=KRT10 PE=1 SV=6
Query Observed Mr(expt) Mr(calc) ppm Miss Score Expect Rank Peptide 771
516.3036 1030.5927 1030.5910 1.60 0 56 2.3e-005 1 R.VLDELTLTK.A 770
928 **453.2444 1356.7114 1356.7110 0.30 1 30 0.016 1 R.QSVEADINGLRR.V**
1045 **854.3906 1706.7667 1706.7649 1.05 0 86 2.9e-008 1 K.GSLGGGFSSGGFSGGSFSR.G**
5. B1AVQ5 **Mass:** 49365 **Score:** 125 **Queries matched:** 4 **emPAI:** 0.13
Mucin-1 subunit alpha OS=Homo sapiens GN=MUC1 PE=2 SV=1
Query Observed Mr(expt) Mr(calc) ppm Miss Score Expect Rank Peptide
837 **567.3196 1132.6246 1132.6241 0.52 0 48 0.00027 1 K.QGGFLGLSNIK.F** 838
941 **693.8518 1385.6890 1385.6901 -0.78 0 77 3.6e-007 1 R.DISEMFLQIYK.Q** 942
Proteins matching the same set of peptides:
B4DWK6 **Mass:** 57362 **Score:** 125 **Queries matched:** 4
cDNA FLJ60927, highly similar to Mucin-1 OS=Homo sapiens PE=2 SV=1
A5YRU5 **Mass:** 30583 **Score:** 125 **Queries matched:** 4
MUC1 isoform Z-LSP OS=Homo sapiens GN=MUC1 PE=2 SV=1
A5YRU7 **Mass:** 23793 **Score:** 125 **Queries matched:** 4
MUC1 isoform T7 OS=Homo sapiens GN=MUC1 PE=2 SV=1
A5YRU8 **Mass:** 26639 **Score:** 125 **Queries matched:** 4
MUC1 isoform T8 OS=Homo sapiens GN=MUC1 PE=2 SV=1
A5YRU9 **Mass:** 12249 **Score:** 125 **Queries matched:** 4
MUC1 isoform T9 OS=Homo sapiens GN=MUC1 PE=4 SV=1
A6ZID5 **Mass:** 29402 **Score:** 125 **Queries matched:** 4
MUC1 isoform M2 OS=Homo sapiens GN=MUC1 PE=2 SV=1
A6ZIE1 **Mass:** 11261 **Score:** 125 **Queries matched:** 4
MUC1 isoform SV4-LSP OS=Homo sapiens GN=MUC1 PE=4 SV=1
B6EC96 **Mass:** 11260 **Score:** 125 **Queries matched:** 4
MUC1 isoform J8 OS=Homo sapiens GN=MUC1 PE=4 SV=1
B6ECA3 **Mass:** 29700 **Score:** 125 **Queries matched:** 4
MUC1 isoform J14 OS=Homo sapiens GN=MUC1 PE=2 SV=1
B6ECB0 **Mass:** 12447 **Score:** 125 **Queries matched:** 4
MUC1 isoform J21 OS=Homo sapiens GN=MUC1 PE=4 SV=1
B6ECB1 **Mass:** 11249 **Score:** 125 **Queries matched:** 4
MUC1 isoform J22 OS=Homo sapiens GN=MUC1 PE=4 SV=1
B6ECB2 **Mass:** 19749 **Score:** 125 **Queries matched:** 4
MUC1 isoform J23 OS=Homo sapiens GN=MUC1 PE=2 SV=1
Q7Z536 **Mass:** 16424 **Score:** 125 **Queries matched:** 4
Mucin short variant SV10 OS=Homo sapiens GN=MUC1 PE=2 SV=1
P15941 **Mass:** 122200 **Score:** 125 **Queries matched:** 4
Mucin-1 OS=Homo sapiens GN=MUC1 PE=1 SV=3
6. P35527 **Mass:** 62255 **Score:** 90 **Queries matched:** 2 **emPAI:** 0.10

Keratin, type I cytoskeletal 9 OS=Homo sapiens GN=KRT9 PE=1 SV=3
Query Observed Mr(expt) Mr(calc) ppm Miss Score Expect Rank Peptide 792
530.7853 1059.5561 1059.5560 0.02 0 38 0.0053 1 K.TLLDIDNTR.M
1091 **613.3273 1836.9600 1836.9581 1.04 0 53 7.1e-005 1 R.HGVQELEIELQSQLSK.K**
7. H0Y4K8 **Mass:** 26596 **Score:** 45 **Queries matched:** 1 **emPAI:** 0.12
Ugl-Y3 (Fragment) OS=Homo sapiens GN=FN1 PE=2 SV=1
Query Observed Mr(expt) Mr(calc) ppm Miss Score Expect Rank Peptide
1120 **957.0023 1911.9900 1911.9902 -0.10 0 45 0.00046 1 R.SSPVVIDASTAIDAPSNLR.F**
Proteins matching the same set of peptides:
H0Y7Z1 **Mass:** 121669 **Score:** 45 **Queries matched:** 1
Ugl-Y3 (Fragment) OS=Homo sapiens GN=FN1 PE=2 SV=1
B4DTK1 **Mass:** 112204 **Score:** 45 **Queries matched:** 1
cDNA FLJ53292, highly similar to Homo sapiens fibronectin 1 (FN1), transcript variant 5, mRNA OS=Homo sapiens PE=2 SV=1
B4DU16 **Mass:** 99064 **Score:** 45 **Queries matched:** 1
cDNA FLJ54550, highly similar to Homo sapiens fibronectin 1 (FN1), transcript variant 6, mRNA OS=Homo sapiens PE=2 SV=1
Q5CZ99 **Mass:** 85628 **Score:** 45 **Queries matched:** 1
Putative uncharacterized protein DKFZp686I1370 (Fragment) OS=Homo sapiens GN=DKFZp686I1370 PE=2 SV=1
A6YID2 **Mass:** 66823 **Score:** 45 **Queries matched:** 1
Fibronectin splice variant A (Fragment) OS=Homo sapiens GN=FN1 PE=2 SV=1
A6YID3 **Mass:** 64111 **Score:** 45 **Queries matched:** 1
Fibronectin splice variant B (Fragment) OS=Homo sapiens GN=FN1 PE=2 SV=1
A6YID4 **Mass:** 57016 **Score:** 45 **Queries matched:** 1
Fibronectin splice variant C (Fragment) OS=Homo sapiens GN=FN1 PE=2 SV=1
A6YID5 **Mass:** 57053 **Score:** 45 **Queries matched:** 1
Fibronectin splice variant D (Fragment) OS=Homo sapiens GN=FN1 PE=2 SV=1
A6YID6 **Mass:** 70230 **Score:** 45 **Queries matched:** 1
Fibronectin splice variant E (Fragment) OS=Homo sapiens GN=FN1 PE=2 SV=1
Q6MZM7 **Mass:** 242944 **Score:** 45 **Queries matched:** 1
Putative uncharacterized protein DKFZp686O12165 (Fragment) OS=Homo sapiens GN=DKFZp686O12165 PE=1 SV=1
Q6N084 **Mass:** 114328 **Score:** 45 **Queries matched:** 1
Putative uncharacterized protein DKFZp686L11144 (Fragment) OS=Homo sapiens GN=DKFZp686L11144 PE=2 SV=1
B7ZLE5 **Mass:** 250136 **Score:** 45 **Queries matched:** 1
FN1 protein OS=Homo sapiens GN=FN1 PE=2 SV=1
F8W7G7 **Mass:** 246447 **Score:** 45 **Queries matched:** 1
Ugl-Y3 OS=Homo sapiens GN=FN1 PE=2 SV=1
Q9UQS6 **Mass:** 38632 **Score:** 45 **Queries matched:** 1
Fibronectin (Fragment) OS=Homo sapiens PE=2 SV=1
Q59G22 **Mass:** 111785 **Score:** 45 **Queries matched:** 1
Fibronectin 1 variant (Fragment) OS=Homo sapiens PE=2 SV=1
P02751 **Mass:** 266052 **Score:** 45 **Queries matched:** 1
Fibronectin OS=Homo sapiens GN=FN1 PE=1 SV=4
O95617 **Mass:** 41345 **Score:** 45 **Queries matched:** 1
Fibronectin (Fragment) OS=Homo sapiens PE=2 SV=1
Q68CX6 **Mass:** 237220 **Score:** 43 **Queries matched:** 1
Putative uncharacterized protein DKFZp686O13149 OS=Homo sapiens GN=DKFZp686O13149 PE=2 SV=1
8. A0N4V7 **Mass:** 2269 **Score:** 42 **Queries matched:** 9 **emPAI:** 1.75
HCG2039797 (Fragment) OS=Homo sapiens GN=Tcr-alpha PE=4 SV=1
Query Observed Mr(expt) Mr(calc) ppm Miss Score Expect Rank Peptide
576 **421.7577 841.5009 841.5022 -1.44 1 42 0.0013 1 K.GITLSVRP.-** 577 578 579 580 582 583 584 585
9. L0R5A1 **Mass:** 11752 **Score:** 38 **Queries matched:** 4 **emPAI:** 0.63
Alternative protein CSF2RB OS=Homo sapiens GN=CSF2RB PE=4 SV=1
Query Observed Mr(expt) Mr(calc) ppm Miss Score Expect Rank Peptide
608 **428.7661 855.5176 855.5178 -0.26 0 38 0.0025 1 R.LASIVTPR.N** 607 609 611
10. H7C4T5 **Mass:** 36596 **Score:** 26 **Queries matched:** 2 **emPAI:** 0.09
Muscleblind-like protein 1 (Fragment) OS=Homo sapiens GN=MBNL1 PE=4 SV=1
Query Observed Mr(expt) Mr(calc) ppm Miss Score Expect Rank Peptide
632 **435.7742 869.5338 869.5335 0.39 0 26 0.021 1 -.VVSVTPIR.D** 630

Sample T9 red box

Database : HumanUniprot uniprot140506 (136615 sequences; 43285968 residues)
Enzyme : Trypsin
Fixed modifications : Carbamidomethyl (C)
Variable modifications : Deamidated (NQ),Oxidation (M)
Mass values : Monoisotopic
Protein Mass : Unrestricted
Peptide Mass Tolerance : ± 3 ppm
Fragment Mass Tolerance: ± 0.6 Da
Max Missed Cleavages : 1
Instrument type : ESI-TRAP
Number of queries : 1748
Protein hits : **H6VRF8** Keratin 1 OS=Homo sapiens GN=KRT1 PE=3 SV=1
P13645 Keratin, type I cytoskeletal 10 OS=Homo sapiens GN=KRT10 PE=1 SV=6
H6VRG2 Keratin 1 OS=Homo sapiens GN=KRT1 PE=3 SV=1
P35908 Keratin, type II cytoskeletal 2 epidermal OS=Homo sapiens GN=KRT2 PE=1 SV=2
P35527 Keratin, type I cytoskeletal 9 OS=Homo sapiens GN=KRT9 PE=1 SV=3
A0N4V7 HCG2039797 (Fragment) OS=Homo sapiens GN=Tcr-alpha PE=4 SV=1 **L0R5A1** Alternative protein CSF2RB OS=Homo sapiens GN=CSF2RB PE=4 SV=1 **B7Z945** Protein LOC100132891 OS=Homo sapiens GN=LOC100132891 PE=2 SV=1 **Q8NBH6** cDNA PSEC0266 fis, clone NT2RP3003649, highly similar to Homo sapiens fibulin-1D mRNA OS=Homo sapiens PE=2 SV=1

Select Summary Report

Significance threshold p< Max. number of hits

Standard scoring MudPIT scoring Ions score or expect cut-off Show sub-sets

Show pop-ups Suppress pop-ups Sort unassigned Decreasing Score Require bold red

1. H6VRF8 **Mass:** 66184 **Score:** 1463 **Queries matched:** 53 **emPAI:** 2.48
Keratin 1 OS=Homo sapiens GN=KRT1 PE=3 SV=1

Query	Observed	Mr(expt)	Mr(calc)	ppm	Miss	Score	Expect	Rank	Peptide	
508	416.7476	831.4806	831.4814	-0.95	0	31	0.015	1	K.SISISVAR.G	509
588	437.7532	873.4919	873.4920	-0.05	0	37	0.0081	1	R.SLVNLGGSK.S	
698	487.2692	972.5239	972.5240	-0.12	0	62	2.6e-005	1	K.IEISELNR.V	699 700
722	500.2269	998.4392	998.4379	1.32	0	49	0.00016	1	K.DVDGAYMTK.V	721
750	517.2613	1032.5081	1032.5087	-0.62	0	64	1.1e-005	1	R.TLLEGEESR.M	749 751
776	533.2632	1064.5118	1064.5138	-1.85	0	60	1.8e-005	1	K.AQYEDIAQK.S	777
846	590.3044	1178.5942	1178.5931	0.89	0	85	8.4e-008	1	K.YEELQITAGR.H	845 847
894	633.3220	1264.6295	1264.6299	-0.31	0	75	7.2e-007	1	R.TNAENEFVTIK.K	893
901	639.3587	1276.7029	1276.7027	0.17	0	75	3.9e-007	1	K.LALDLEIATYR.T	900
909	650.7684	1299.5222	1299.5224	-0.09	0	68	5.9e-007	1	K.NMQDMVEDYR.N	
911	651.8613	1301.7081	1301.7078	0.18	0	121	1.1e-011	1	R.SLDLDSIIAEVK.A	910 912
934	670.8388	1339.6631	1339.6619	0.92	1	76	5e-007	1	K.SKAEAESLYQSK.Y	935
962	692.3487	1382.6829	1382.6830	-0.09	0	74	8e-007	1	K.SLNNQFASFIDK.V	963
971	465.2486	1392.7240	1392.7249	-0.61	1	49	0.00015	1	R.TNAENEFVTIKK.D	969 970
1008	738.3782	1474.7419	1474.7416	0.18	0	78	3.5e-007	1	K.WELLQQVDTSTR.T	1007 1009
1012	738.3970	1474.7795	1474.7780	1.01	0	84	5.9e-008	1	R.FLEQQNQVLQTK.W	1010 1011
1038	508.6014	1522.7823	1522.7813	0.65	0	31	0.012	1	R.LLRDYQELMNTK.L	
1067	546.9574	1637.8504	1637.8525	-1.31	1	33	0.0052	1	K.SLNNQFASFIDKVR.F	1068 1069
1090	858.9311	1715.8476	1715.8438	2.18	0	83	1.1e-007	1	K.QISNLQQSISDAEQR.G	

1100 **883.3710 1764.7275 1764.7275 0.01 0 40 0.00036 1 R.FSSCGGGGGSFGAGGGFGSR.S**
1183 **662.6355 1984.8848 1984.8870 -1.10 1 46 0.00032 1 R.LDSELKNMQDMVEDYR.N** 1184
1222 **538.2751 2149.0714 2149.0705 0.45 1 32 0.014 1 R.THNLEPYFESFINNLRR.R** 1221 1223
1280 **762.7125 2285.1156 2285.1175 -0.83 1 33 0.0078 1 K.AEAESLYQSKYEELQITAGR.H**
1354 **861.0599 2580.1580 2580.1545 1.36 0 32 0.0063 1 R.MSGECAPNVSVSVSTSHTTISGGGSR.G**
1447 **978.1792 2931.5159 2931.5090 2.35 1 43 0.00066 1 R.FLEQQNQVLQTKWELLQQVDTSTR.T** 1445 1446
Proteins matching the same set of peptides:
H6VRG0 **Mass:** 66101 **Score:** 1463 **Queries matched:** 53
Keratin 1 OS=Homo sapiens GN=KRT1 PE=3 SV=1
H6VRG1 **Mass:** 66257 **Score:** 1463 **Queries matched:** 53
Keratin 1 OS=Homo sapiens GN=KRT1 PE=2 SV=1
H6VRG3 **Mass:** 66242 **Score:** 1463 **Queries matched:** 53
Keratin 1 OS=Homo sapiens GN=KRT1 PE=3 SV=1
P04264 **Mass:** 66170 **Score:** 1463 **Queries matched:** 53
Keratin, type II cytoskeletal 1 OS=Homo sapiens GN=KRT1 PE=1 SV=6
2. P13645 **Mass:** 59020 **Score:** 1456 **Queries matched:** 64 **emPAI:** 2.64
Keratin, type I cytoskeletal 10 OS=Homo sapiens GN=KRT10 PE=1 SV=6
Query Observed Mr(expt) Mr(calc) ppm Miss Score Expect Rank Peptide
464 **404.2034 806.3922 806.3923 -0.04 0 47 0.00029 1 R.LAADDFR.L** 463
471 **405.2236 808.4327 808.4330 -0.47 0 45 0.00066 1 R.LASYLDK.V** 472 473
713 **497.2540 992.4935 992.4927 0.85 0 57 5.1e-005 1 K.YENEVALR.Q** 711 712
725 **502.2802 1002.5458 1002.5458 0.03 1 24 0.053 1 K.SEITELRR.N**
747 **516.3025 1030.5905 1030.5910 -0.54 0 66 1.9e-006 1 R.VLDELTLTK.A** 748
775 **532.8094 1063.6043 1063.6026 1.67 1 47 0.00024 1 R.LASYLDKVR.A** 772 773 774
791 **545.7700 1089.5254 1089.5237 1.55 0 66 4.1e-006 1 K.VTMQNLNDR.L** 790
805 **555.2489 1108.4832 1108.4825 0.64 0 64 4e-006 1 K.DAEAWFNEK.S** 804 806
841 **583.2966 1164.5786 1164.5775 0.98 0 59 3.5e-005 1 R.LENEIQTYR.S** 839 840
859 **601.3117 1200.6088 1200.6098 -0.83 0 71 2.1e-006 1 R.QSVEADINGLR.R**
872 **617.8442 1233.6738 1233.6717 1.69 1 49 0.00019 1 R.LKYENEVALR.Q** 871
889 **631.8022 1261.5899 1261.5899 0.00 0 89 1.1e-008 1 R.SLLEGEGSSGGGGR.G** 890 891
939 **453.2440 1356.7102 1356.7110 -0.53 1 (40) 0.0018 1 R.QSVEADINGLRR.V** 940
943 **453.5714 1357.6924 1357.6950 -1.92 1 43 0.001 1 R.QSVEADINGLRR.V**
946 **683.3250 1364.6354 1364.6320 2.45 0 77 3.6e-007 1 R.SQYEQLAEQNR.K** 945 947
959 **691.3279 1380.6413 1380.6408 0.36 0 87 2.9e-008 1 R.ALEESNYELEGK.I** 960 961
965 **695.8443 1389.6741 1389.6736 0.40 0 88 2.8e-008 1 K.QSLEASLAETEGR.Y**
987 **717.8894 1433.7643 1433.7626 1.17 1 44 0.00061 1 K.IRLENEIQTYR.S** 984 985 986 988
1030 **498.5833 1492.7282 1492.7270 0.80 1 45 0.00051 1 R.SQYEQLAEQNRK.D** 1028 1029
1086 **854.3900 1706.7655 1706.7649 0.37 0 139 1.3e-013 1 K.GSLGGGFSSGGFSGGSFSR.G** 1082 1083 1084 1085
1191 **998.9910 1995.9674 1995.9636 1.86 0 92 1.1e-008 1 K.ELTTEIDNNIEQISSYK.S** 1189 1190
1253 **738.0368 2211.0887 2211.0906 -0.87 1 75 5.6e-007 1 K.SKELTTEIDNNIEQISSYK.S** 1254 1255
1437 **968.8001 2903.3784 2903.3753 1.06 0 32 0.012 1 R.NVSTGDVNVEMNAAPGVDLTQLLNNMR.S** 1435 1436
1477 **1018.2140 3051.6201 3051.6200 0.03 1 49 8.3e-005 1 K.TIDDLKNQILNLTTDNANILLQIDNAR.L** 1478 1480
3. H6VRG2 **Mass:** 66197 **Score:** 1442 **Queries matched:** 52 **emPAI:** 2.48
Keratin 1 OS=Homo sapiens GN=KRT1 PE=3 SV=1
Query Observed Mr(expt) Mr(calc) ppm Miss Score Expect Rank Peptide
508 416.7476 831.4806 831.4814 -0.95 0 31 0.015 1 K.SISISVAR.G 509
588 437.7532 873.4919 873.4920 -0.05 0 37 0.0081 1 R.SLVNLGGSK.S
698 487.2692 972.5239 972.5240 -0.12 0 62 2.6e-005 1 K.IEISELNR.V 699 700
722 500.2269 998.4392 998.4379 1.32 0 49 0.00016 1 K.DVDGAYMTK.V 721
750 517.2613 1032.5081 1032.5087 -0.62 0 64 1.1e-005 1 R.TLLEGEESR.M 749 751
776 533.2632 1064.5118 1064.5138 -1.85 0 60 1.8e-005 1 K.AQYEDIAQK.S 777
846 590.3044 1178.5942 1178.5931 0.89 0 85 8.4e-008 1 K.YEELQITAGR.H 845 847
894 633.3220 1264.6295 1264.6299 -0.31 0 75 7.2e-007 1 R.TNAENEFVTIK.K 893
901 639.3587 1276.7029 1276.7027 0.17 0 75 3.9e-007 1 K.LALDLEIATYR.T 900
909 650.7684 1299.5222 1299.5224 -0.09 0 68 5.9e-007 1 K.NMQDMVEDYR.N
931 **665.3685 1328.7225 1328.7187 2.87 0 100 1.6e-009 1 R.NLDLDSIIAEVK.A** 930
934 670.8388 1339.6631 1339.6619 0.92 1 76 5e-007 1 K.SKAEAESLYQSK.Y 935
962 692.3487 1382.6829 1382.6830 -0.09 0 74 8e-007 1 K.SLNNQFASFIDK.V 963
971 465.2486 1392.7240 1392.7249 -0.61 1 49 0.00015 1 R.TNAENEFVTIKK.D 969 970
1008 738.3782 1474.7419 1474.7416 0.18 0 78 3.5e-007 1 K.WELLQQVDTSTR.T 1007 1009
1012 738.3970 1474.7795 1474.7780 1.01 0 84 5.9e-008 1 R.FLEQQNQVLQTK.W 1010 1011
1038 508.6014 1522.7823 1522.7813 0.65 1 31 0.012 1 R.LLRDYQELMNTK.L
1067 546.9574 1637.8504 1637.8525 -1.31 1 33 0.0052 1 K.SLNNQFASFIDKVR.F 1068 1069
1090 858.9311 1715.8476 1715.8438 2.18 0 83 1.1e-007 1 K.QISNLQQSISDAEQR.G
1100 883.3710 1764.7275 1764.7275 0.01 0 40 0.00036 1 R.FSSCGGGGGSFGAGGGFGSR.S
1183 662.6355 1984.8848 1984.8870 -1.10 1 46 0.00032 1 R.LDSELKNMQDMVEDYR.N 1184
1222 538.2751 2149.0714 2149.0705 0.45 1 32 0.014 1 R.THNLEPYFESFINNLRR.R 1221 1223
1280 762.7125 2285.1156 2285.1175 -0.83 1 33 0.0078 1 K.AEAESLYQSKYEELQITAGR.H
1354 861.0599 2580.1580 2580.1545 1.36 0 32 0.0063 1 R.MSGECAPNVSVSVSTSHTTISGGGSR.G
1447 978.1792 2931.5159 2931.5090 2.35 1 43 0.00066 1 R.FLEQQNQVLQTKWELLQQVDTSTR.T 1445 1446
4. P35908 **Mass:** 65678 **Score:** 981 **Queries matched:** 31 **emPAI:** 0.92
Keratin, type II cytoskeletal 2 epidermal OS=Homo sapiens GN=KRT2 PE=1 SV=2

Query Observed Mr(expt) Mr(calc) ppm Miss Score Expect Rank Peptide
698 487.2692 972.5239 972.5240 -0.12 0 62 2.6e-005 1 K.IEISELNR.V 699 700
755 **519.2667 1036.5189 1036.5189 -0.03 0 68 3.6e-006 1 R.YLDGLTAER.T** 754 756
802 **554.2760 1106.5375 1106.5356 1.69 0 61 2.3e-005 1 K.AQYEEIAQR.S** 801
857 **597.3123 1192.6101 1192.6088 1.08 0 81 2.2e-007 1 K.YEELQVTVGR.H** 856 858
862 **604.8112 1207.6078 1207.6085 -0.55 0 84 8.6e-008 1 R.TAAENDFVTLK.K** 863
880 **627.8076 1253.6006 1253.6001 0.42 0 110 1.9e-010 1 R.GFSSGSAVVSGGSR.R** 881 882
923 **440.8664 1319.5773 1319.5756 1.27 0 76 1.6e-007 1 R.HGGGGGGFGGGGFGSR.S** 921 922
929 **665.3236 1328.6327 1328.6320 0.52 0 93 6.2e-009 1 K.NVQDAIADAEQR.G** 927 928
931 665.3685 1328.7225 1328.7187 2.87 0 100 1.6e-009 1 R.NLDLDSIIAEVK.A 930
933 **446.2421 1335.7044 1335.7034 0.72 1 40 0.0018 1 R.TAAENDFVTLKK.D**
950 **686.3606 1370.7066 1370.7041 1.80 0 62 1.5e-005 1 K.LNDLEEALQQAK.E**
1012 738.3970 1474.7795 1474.7780 1.01 0 84 5.9e-008 1 R.FLEQQNQVLQTK.W 1010 1011
1271 **752.6859 2255.0359 2255.0376 -0.74 1 24 0.052 1 R.TSQNSELNNMQDLVEDYKK.K**
1482 **1022.1699 3063.4878 3063.4819 1.93 0 35 0.0029 1 K.VLYDAEISQIHQSVTDTNVILSMDNSR.N**
5. P35527 **Mass:** 62255 **Score:** 669 **Queries matched:** 37 **emPAI:** 1.19
Keratin, type I cytoskeletal 9 OS=Homo sapiens GN=KRT9 PE=1 SV=3
Query Observed Mr(expt) Mr(calc) ppm Miss Score Expect Rank Peptide
471 405.2236 808.4327 808.4330 -0.47 0 45 0.00066 1 R.LASYLDK.V 472 473
618 **449.2101 896.4056 896.4062 -0.62 0 34 0.0052 1 R.MTLDDFR.I**
771 **530.7853 1059.5560 1059.5560 -0.07 0 52 0.00018 1 K.TLLDIDNTR.M** 769 770
834 **579.2993 1156.5841 1156.5836 0.40 0 89 1e-008 1 R.QGVDADINGLR.Q** 832 833
835 **579.7915 1157.5684 1157.5677 0.68 0 (64) 6.8e-006 1 R.QGVDADINGLR.Q**
852 **595.8072 1189.5998 1189.6013 -1.26 0 56 5.2e-005 1 R.QVLDNLTMEK.S** 853 854
917 **436.5648 1306.6727 1306.6703 1.79 1 51 8.1e-005 1 R.IKFEMEQNLR.Q** 914 915 916 918
1135 **919.4882 1836.9617 1836.9581 1.98 0 77 2.1e-007 1 R.HGVQELEIELQSQLSK.K** 1133 1134
1145 **617.9805 1850.9198 1850.9196 0.10 1 46 0.00042 1 K.TLNDMRQEYEQLIAK.N** 1144 1146 1147
1154 **623.3138 1866.9195 1866.9145 2.69 1 (36) 0.005 1 K.TLNDMRQEYEQLIAK.N** 1152 1153
1180 **656.0262 1965.0569 1965.0531 1.95 1 49 6.4e-005 1 R.HGVQELEIELQSQLSKK.A**
1331 **837.3821 2509.1246 2509.1245 0.04 0 78 1.5e-007 1 K.EIETYHNLLEGGQEDFESSGAGK.I** 1330 1332
1387 **902.3914 2704.1525 2704.1539 -0.52 0 34 0.0019 1 R.GGGGSFGYSYGGGSGGGFSASSLGGGFGGGSR.G**
1503 **1088.8429 3263.5069 3263.5066 0.10 0 55 2.5e-005 1 K.DIENQYETQITQIEHEVSSSGQEVQSSAK.E** 1501 1502
6. A0N4V7 **Mass:** 2269 **Score:** 47 **Queries matched:** 13 **emPAI:** 6.57
HCG2039797 (Fragment) OS=Homo sapiens GN=Tcr-alpha PE=4 SV=1
Query Observed Mr(expt) Mr(calc) ppm Miss Score Expect Rank Peptide
539 **421.7589 841.5033 841.5022 1.32 1 47 0.00048 1 K.GITLSVRP.-** 527 528 529 530 531 532 533 534 535 536 537 538
7. L0R5A1 **Mass:** 11752 **Score:** 34 **Queries matched:** 3 **emPAI:** 0.28
Alternative protein CSF2RB OS=Homo sapiens GN=CSF2RB PE=4 SV=1
Query Observed Mr(expt) Mr(calc) ppm Miss Score Expect Rank Peptide
555 **428.7658 855.5170 855.5178 -0.87 0 34 0.0059 1 R.LASIVTPR.N** 556 557
8. B7Z945 **Mass:** 20378 **Score:** 32 **Queries matched:** 2 **emPAI:** 0.16
Protein LOC100132891 OS=Homo sapiens GN=LOC100132891 PE=2 SV=1
Query Observed Mr(expt) Mr(calc) ppm Miss Score Expect Rank Peptide
908 **648.3389 1294.6633 1294.6629 0.31 1 32 0.0073 1 R.VGAQVQNPRAPR.K** 907
9. Q8NBH6 **Mass:** 74066 **Score:** 27 **Queries matched:** 2 **emPAI:** 0.04
cDNA PSEC0266 fis, clone NT2RP3003649, highly similar to Homo sapiens fibulin-1D mRNA OS=Homo sapiens PE=2 SV=1
Query Observed Mr(expt) Mr(calc) ppm Miss Score Expect Rank Peptide
1050 **517.2836 1548.8290 1548.8300 -0.62 1 27 0.028 1 R.EFTRPEEIIFLR.A** 1051
Proteins matching the same set of peptides:
P23142 **Mass:** 81268 **Score:** 27 **Queries matched:** 2
Fibulin-1 OS=Homo sapiens GN=FBLN1 PE=1 SV=4

Sample T9 green box

Database : HumanUniprot uniprot140506 (136615 sequences; 43285968 residues)
Enzyme : Trypsin
Fixed modifications : Carbamidomethyl (C)
Variable modifications : Deamidated (NQ),Oxidation (M)
Mass values : Monoisotopic
Protein Mass : Unrestricted
Peptide Mass Tolerance : ± 3 ppm
Fragment Mass Tolerance: ± 0.6 Da
Max Missed Cleavages : 1
Instrument type : ESI-TRAP
Number of queries : 1700
Protein hits : **Q1KLZ0** HCG15971, isoform CRA_a OS=Homo sapiens GN=PS1TP5BP1 PE=2 SV=1
D2JYH4 Actin, alpha 2, smooth muscle, aorta OS=Homo sapiens GN=ACTA2 PE=2 SV=1
J3KR22 C-type lectin domain family 10 member A OS=Homo sapiens GN=CLEC10A PE=2 SV=1
B4DE59 cDNA FLJ60424, highly similar to Junction plakoglobin OS=Homo sapiens PE=2 SV=1
P13645 Keratin, type I cytoskeletal 10 OS=Homo sapiens GN=KRT10 PE=1 SV=6
Q562R1 Beta-actin-like protein 2 OS=Homo sapiens GN=ACTBL2 PE=1 SV=2
Q0QET7 Glyceraldehyde-3-phosphate dehydrogenase (Fragment) OS=Homo sapiens GN=GAPDH PE=2 SV=1
H6VRF8 Keratin 1 OS=Homo sapiens GN=KRT1 PE=3 SV=1
P35908 Keratin, type II cytoskeletal 2 epidermal OS=Homo sapiens GN=KRT2 PE=1 SV=2
B0YJC4 Vimentin OS=Homo sapiens GN=VIM PE=3 SV=1
E2DH89 MHC class I antigen (Fragment) OS=Homo sapiens GN=HLA-B PE=3 SV=1
I0CMK4 Beta-tubulin 4Q isoform 1 (Fragment) OS=Homo sapiens GN=TUBB4Q PE=3 SV=1
A0N4V7 HCG2039797 (Fragment) OS=Homo sapiens GN=Tcr-alpha PE=4 SV=1 **L0R5A1** Alternative protein CSF2RB OS=Homo sapiens GN=CSF2RB PE=4 SV=1
B7Z945 Protein LOC100132891 OS=Homo sapiens GN=LOC100132891 PE=2 SV=1
I3L4V6 Nucleoredoxin (Fragment) OS=Homo sapiens GN=NXN PE=4 SV=1 **Q59E90** Mannosidase, alpha, class 2B, member 1 variant (Fragment) OS=Homo sapiens PE=2 SV=1

Select Summary Report

Significance threshold p< Max. number of hits
Standard scoring MudPIT scoring Ions score or expect cut-off Show sub-sets
Show pop-ups Suppress pop-ups Sort unassigned Decreasing Score Require bold red

1. Q1KLZ0 **Mass:** 42052 **Score:** 646 **Queries matched:** 34 **emPAI:** 2.17
HCG15971, isoform CRA_a OS=Homo sapiens GN=PS1TP5BP1 PE=2 SV=1

Query Observed Mr(expt) Mr(calc) ppm Miss Score Expect Rank Peptide
658 **488.7273 975.4401 975.4410 -0.98 0 82 9.1e-008 1 K.AGFAGDDAPR.A** 659 660
801 **566.7668 1131.5191 1131.5197 -0.49 0 79 6.1e-008 1 R.GYSFTTTAER.E** 802
819 **581.3135 1160.6124 1160.6111 1.09 0 59 1.8e-005 1 K.EITALAPSTMK.I** 818 820
830 **391.1947 1170.5623 1170.5638 -1.29 0 36 0.0035 1 R.HQGVMVGMGQK.D**
835 **589.3102 1176.6059 1176.6060 -0.05 0 (53) 0.00011 1 K.EITALAPSTMK.I** 834 836
854 **599.8574 1197.7002 1197.6982 1.66 1 49 0.00012 1 R.AVFPSIVGRPR.H** 851
999 **541.9516 1622.8329 1622.8338 -0.53 1 42 0.00088 1 R.LDLAGRDLTDYLMK.I** 1000

1002 **543.9457 1628.8153 1628.8158 -0.29 1 36 0.12 1 R.GYSFTTTAEREIVR.D** 1003 1004
1013 **547.2833 1638.8281 1638.8287 -0.36 1 (29) 0.019 1 R.LDLAGRDLTDYLMK.I**
1065 **895.9501 1789.8857 1789.8846 0.60 0 62 1.2e-005 1 K.SYELPDGQVITIGNER.F** 1064
1127 **977.5377 1953.0609 1953.0571 1.93 0 51 5e-005 1 R.VAPEEHPVLLTEAPLNPK.A** 1125 1126 1128
1220 **1108.0414 2214.0683 2214.0627 2.53 0 99 2.1e-009 1 K.DLYANTVLSGGTTMYPGIADR.M** 1219 1221
1484 **1061.8782 3182.6128 3182.6071 1.81 0 50 0.0001 1 R.TTGIVMDSGDGVTHTVPIYEGYALPHAILR.L** 1482
1485 1486
1489 **800.6573 3198.6000 3198.6020 -0.61 0 (37) 0.0026 1 R.TTGIVMDSGDGVTHTVPIYEGYALPHAILR.L**
Proteins matching the same set of peptides:
Q8WVW5 **Mass:** 40819 **Score:** 646 **Queries matched:** 34
Putative uncharacterized protein (Fragment) OS=Homo sapiens PE=2 SV=1
Q53G76 **Mass:** 42036 **Score:** 646 **Queries matched:** 34
Beta actin variant (Fragment) OS=Homo sapiens PE=2 SV=1
Q53G99 **Mass:** 42080 **Score:** 646 **Queries matched:** 34
Beta actin variant (Fragment) OS=Homo sapiens PE=2 SV=1
Q53GK6 **Mass:** 42038 **Score:** 646 **Queries matched:** 34
Beta actin variant (Fragment) OS=Homo sapiens PE=2 SV=1
P60709 **Mass:** 42052 **Score:** 646 **Queries matched:** 34
Actin, cytoplasmic 1 OS=Homo sapiens GN=ACTB PE=1 SV=1
P63261 **Mass:** 42108 **Score:** 646 **Queries matched:** 34
Actin, cytoplasmic 2 OS=Homo sapiens GN=ACTG1 PE=1 SV=1
2. D2JYH4 **Mass:** 42381 **Score:** 436 **Queries matched:** 22 **emPAI:** 1.05
Actin, alpha 2, smooth muscle, aorta OS=Homo sapiens GN=ACTA2 PE=2 SV=1
Query Observed Mr(expt) Mr(calc) ppm Miss Score Expect Rank Peptide
658 488.7273 975.4401 975.4410 -0.98 0 82 9.1e-008 1 K.AGFAGDDAPR.A 659 660
796 **565.7784 1129.5422 1129.5404 1.64 0 64 6.1e-006 1 R.GYSFVTTAER.E** 795
819 581.3135 1160.6124 1160.6111 1.09 0 59 1.8e-005 1 K.EITALAPSTMK.I 818 820
830 391.1947 1170.5623 1170.5638 -1.29 0 36 0.0035 1 R.HQGVMVGMGQK.D
835 589.3102 1176.6059 1176.6060 -0.05 0 (53) 0.00011 1 K.EITALAPSTMK.I 834 836
854 599.8574 1197.7002 1197.6982 1.66 1 49 0.00012 1 R.AVFPSIVGRPR.H 851
999 541.9516 1622.8329 1622.8338 -0.53 1 42 0.00088 1 R.LDLAGRDLTDYLMK.I 1000
1013 547.2833 1638.8281 1638.8287 -0.36 1 (29) 0.019 1 R.LDLAGRDLTDYLMK.I
1065 895.9501 1789.8857 1789.8846 0.60 0 62 1.2e-005 1 K.SYELPDGQVITIGNER.F 1064
1130 **652.6877 1955.0413 1955.0364 2.50 0 42 0.00069 1 R.VAPEEHPTLLTEAPLNPK.A** 1129 1131
Proteins matching the same set of peptides:
A8K3K1 **Mass:** 42362 **Score:** 436 **Queries matched:** 22
cDNA FLJ78096, highly similar to Homo sapiens actin, alpha, cardiac muscle (ACTC), mRNA OS=Homo sapiens PE=2 SV=1
Q13707 **Mass:** 37125 **Score:** 436 **Queries matched:** 22
ACTA2 protein (Fragment) OS=Homo sapiens GN=ACTA2 PE=3 SV=1
P62736 **Mass:** 42381 **Score:** 436 **Queries matched:** 22
Actin, aortic smooth muscle OS=Homo sapiens GN=ACTA2 PE=1 SV=1
P63267 **Mass:** 42249 **Score:** 436 **Queries matched:** 22
Actin, gamma-enteric smooth muscle OS=Homo sapiens GN=ACTG2 PE=1 SV=1
P68032 **Mass:** 42334 **Score:** 436 **Queries matched:** 22
Actin, alpha cardiac muscle 1 OS=Homo sapiens GN=ACTC1 PE=1 SV=1
P68133 **Mass:** 42366 **Score:** 436 **Queries matched:** 22
Actin, alpha skeletal muscle OS=Homo sapiens GN=ACTA1 PE=1 SV=1
3. J3KR22 **Mass:** 33175 **Score:** 411 **Queries matched:** 32 **emPAI:** 2.58
C-type lectin domain family 10 member A OS=Homo sapiens GN=CLEC10A PE=2 SV=1
Query Observed Mr(expt) Mr(calc) ppm Miss Score Expect Rank Peptide
744 **535.8144 1069.6143 1069.6132 1.10 0 74 7.4e-007 1 R.VQQLVQDLK.K** 743 745
781 **561.8234 1121.6323 1121.6305 1.57 0 46 0.00031 1 K.NAHLVVINSR.E** 776 777 778 779 780
844 **592.3169 1182.6192 1182.6179 1.13 0 43 0.00037 1 R.QAVHSEMLLR.V** 838 839 840 841 842 843
849 **398.2029 1191.5870 1191.5884 -1.18 1 28 0.025 1 K.AEVEGFKQER.Q**
860 **599.8617 1197.7089 1197.7081 0.61 1 67 1.7e-006 1 R.VQQLVQDLKK.L** 855 856 857 858 859
1181 **532.0347 2124.1097 2124.1076 0.99 1 37 0.0024 1 K.NAHLVVINSREEQNFVQK.Y** 1177 1182
1184 **709.3699 2125.0879 2125.0916 -1.74 1 (21) 0.1 1 K.NAHLVVINSREEQNFVQK.Y**
1185 **709.3730 2125.0972 2125.0916 2.64 1 (27) 0.03 1 K.NAHLVVINSREEQNFVQK.Y**
1189 **1066.4928 2130.9711 2130.9721 -0.47 0 (79) 1.9e-007 1 K.YLGSAYTWMGLSDPEGAWK.W**
1191 **1074.4879 2146.9612 2146.9670 -2.69 0 82 8.6e-008 1 K.YLGSAYTWMGLSDPEGAWK.W** 1192
1466 **1053.7857 3158.3352 3158.3359 -0.22 1 35 0.00074 1 R.WNDDVCQRPYHWVCEAGLGQTSQESH.-**
4. B4DE59 **Mass:** 62862 **Score:** 256 **Queries matched:** 7 **emPAI:** 0.21
cDNA FLJ60424, highly similar to Junction plakoglobin OS=Homo sapiens PE=2 SV=1
Query Observed Mr(expt) Mr(calc) ppm Miss Score Expect Rank Peptide
448 **404.2034 806.3922 806.3923 -0.13 0 43 0.00068 1 R.LAADDFR.T**
721 **521.3067 1040.5989 1040.5978 1.00 0 48 0.0002 1 R.IVLQIDNAR.L** 719 720
914 **695.3457 1388.6769 1388.6783 -1.03 0 84 4.7e-008 1 K.AALEDTLAETEAR.F** 915
1154 **1007.5091 2013.0036 2013.0015 1.02 0 81 1.3e-007 1 R.GQVGGQVSVEVDSAPGTDLAK.I**
Proteins matching the same set of peptides:
P08727 **Mass:** 44079 **Score:** 256 **Queries matched:** 7
Keratin, type I cytoskeletal 19 OS=Homo sapiens GN=KRT19 PE=1 SV=4
5. P13645 **Mass:** 59020 **Score:** 207 **Queries matched:** 4 **emPAI:** 0.17

Keratin, type I cytoskeletal 10 OS=Homo sapiens GN=KRT10 PE=1 SV=6
Query Observed Mr(expt) Mr(calc) ppm Miss Score Expect Rank Peptide
448 404.2034 806.3922 806.3923 -0.13 0 43 0.00068 1 R.LAADDFR.L
909 **691.3278 1380.6411 1380.6408 0.16 0 65 4.3e-006 1 R.ALEESNYELEGK.I** 910
1042 **854.3896 1706.7647 1706.7649 -0.13 0 99 1.7e-009 1 K.GSLGGGFSSGGFSGGSFSR.G**
6. Q562R1 **Mass:** 42318 **Score:** 173 **Queries matched:** 9 **emPAI:** 0.43
Beta-actin-like protein 2 OS=Homo sapiens GN=ACTBL2 PE=1 SV=2
Query Observed Mr(expt) Mr(calc) ppm Miss Score Expect Rank Peptide
830 391.1947 1170.5623 1170.5638 -1.29 0 36 0.0035 1 R.HQGVMVGMGQK.D
999 541.9516 1622.8329 1622.8338 -0.53 1 42 0.00088 1 R.LDLAGRDLTDYLMK.I 1000
1013 547.2833 1638.8281 1638.8287 -0.36 1 (29) 0.019 1 R.LDLAGRDLTDYLMK.I
1065 895.9501 1789.8857 1789.8846 0.60 0 62 1.2e-005 1 R.SYELPDGQVITIGNER.F 1064
1126 652.0274 1953.0603 1953.0571 1.64 0 32 0.0039 3 R.VAPDEHPILLTEAPLNPK.I 1124 1125
7. Q0QET7 **Mass:** 24776 **Score:** 153 **Queries matched:** 4 **emPAI:** 0.44
Glyceraldehyde-3-phosphate dehydrogenase (Fragment) OS=Homo sapiens GN=GAPDH PE=2 SV=1
Query Observed Mr(expt) Mr(calc) ppm Miss Score Expect Rank Peptide
920 **706.3995 1410.7845 1410.7831 1.03 0 52 5.6e-005 1 R.GALQNIIPASTGAAK.A** 921
978 **765.9027 1529.7908 1529.7872 2.38 0 40 0.0034 1 R.VPTANVSVVDLTCR.L**
1088 **917.4621 1832.9097 1832.9124 -1.52 0 61 1.6e-005 1 K.IISNASCTTNCLAPLAK.V**
Proteins matching the same set of peptides:
Q2TSD0 **Mass:** 36197 **Score:** 153 **Queries matched:** 4
Glyceraldehyde-3-phosphate dehydrogenase OS=Homo sapiens PE=2 SV=1
A4UCT1 **Mass:** 17463 **Score:** 153 **Queries matched:** 4
Glyceraldehyde-3-phosphate dehydrogenase (Fragment) OS=Homo sapiens PE=2 SV=1
E7EUT5 **Mass:** 28024 **Score:** 153 **Queries matched:** 4
Glyceraldehyde-3-phosphate dehydrogenase OS=Homo sapiens GN=GAPDH PE=2 SV=1
V9HVZ4 **Mass:** 36201 **Score:** 153 **Queries matched:** 4
Epididymis secretory sperm binding protein Li 162eP OS=Homo sapiens GN=HEL-S-162eP PE=2 SV=1
P04406 **Mass:** 36201 **Score:** 153 **Queries matched:** 4
Glyceraldehyde-3-phosphate dehydrogenase OS=Homo sapiens GN=GAPDH PE=1 SV=3
8. H6VRF8 **Mass:** 66184 **Score:** 142 **Queries matched:** 3 **emPAI:** 0.10
Keratin 1 OS=Homo sapiens GN=KRT1 PE=3 SV=1
Query Observed Mr(expt) Mr(calc) ppm Miss Score Expect Rank Peptide
899 **651.8615 1301.7085 1301.7078 0.50 0 108 2e-010 1 R.SLDLDSIIAEVK.A**
947 **738.3973 1474.7800 1474.7780 1.34 0 34 0.0057 1 R.FLEQQNQVLQTK.W** 946
Proteins matching the same set of peptides:
H6VRF9 **Mass:** 66200 **Score:** 142 **Queries matched:** 3
Keratin 1 OS=Homo sapiens GN=KRT1 PE=3 SV=1
H6VRG0 **Mass:** 66101 **Score:** 142 **Queries matched:** 3
Keratin 1 OS=Homo sapiens GN=KRT1 PE=3 SV=1
H6VRG1 **Mass:** 66257 **Score:** 142 **Queries matched:** 3
Keratin 1 OS=Homo sapiens GN=KRT1 PE=2 SV=1
H6VRG3 **Mass:** 66242 **Score:** 142 **Queries matched:** 3
Keratin 1 OS=Homo sapiens GN=KRT1 PE=3 SV=1
P04264 **Mass:** 66170 **Score:** 142 **Queries matched:** 3
Keratin, type II cytoskeletal 1 OS=Homo sapiens GN=KRT1 PE=1 SV=6
9. P35908 **Mass:** 65678 **Score:** 114 **Queries matched:** 3 **emPAI:** 0.10
Keratin, type II cytoskeletal 2 epidermal OS=Homo sapiens GN=KRT2 PE=1 SV=2
Query Observed Mr(expt) Mr(calc) ppm Miss Score Expect Rank Peptide
877 **627.8076 1253.6006 1253.6001 0.47 0 80 2e-007 1 R.GFSSGSAVVSGGSR.R**
947 738.3973 1474.7800 1474.7780 1.34 0 34 0.0057 1 R.FLEQQNQVLQTK.W 946
10. B0YJC4 **Mass:** 49680 **Score:** 88 **Queries matched:** 2 **emPAI:** 0.13
Vimentin OS=Homo sapiens GN=VIM PE=3 SV=1
Query Observed Mr(expt) Mr(calc) ppm Miss Score Expect Rank Peptide
761 **547.2665 1092.5185 1092.5200 -1.31 0 56 3.6e-005 1 K.FADLSEAANR.N**
1030 **563.6142 1687.8208 1687.8199 0.52 1 32 0.014 1 R.VEVERDNLAEDIMR.L**
Proteins matching the same set of peptides:
B3KRK8 **Mass:** 47062 **Score:** 88 **Queries matched:** 2
cDNA FLJ34494 fis, clone HLUNG2005030, highly similar to VIMENTIN OS=Homo sapiens PE=2 SV=1
V9HWE1 **Mass:** 53676 **Score:** 88 **Queries matched:** 2
Epididymis luminal protein 113 OS=Homo sapiens GN=HEL113 PE=2 SV=1
Q53HU8 **Mass:** 53708 **Score:** 88 **Queries matched:** 2
Vimentin variant (Fragment) OS=Homo sapiens PE=2 SV=1
P08670 **Mass:** 53676 **Score:** 88 **Queries matched:** 2
Vimentin OS=Homo sapiens GN=VIM PE=1 SV=4
11. E2DH89 **Mass:** 21132 **Score:** 48 **Queries matched:** 1 **emPAI:** 0.15
MHC class I antigen (Fragment) OS=Homo sapiens GN=HLA-B PE=3 SV=1
Query Observed Mr(expt) Mr(calc) ppm Miss Score Expect Rank Peptide
1006 **815.4178 1628.8211 1628.8199 0.76 0 48 0.0066 1 R.FIAVGYVDNTQFVR.F**
Proteins matching the same set of peptides:
F2VNK8 **Mass:** 21213 **Score:** 48 **Queries matched:** 1
MHC class I antigen (Fragment) OS=Homo sapiens GN=HLA-B PE=3 SV=1
F2XI33 **Mass:** 31658 **Score:** 48 **Queries matched:** 1

MHC class I antigen (Fragment) OS=Homo sapiens GN=HLA-A PE=3 SV=1
A3RKK0 **Mass:** 21071 **Score:** 48 **Queries matched:** 1
MHC class I antigen (Fragment) OS=Homo sapiens GN=HLA-B PE=3 SV=1
D5FHY9 **Mass:** 21213 **Score:** 48 **Queries matched:** 1
MHC class I antigen (Fragment) OS=Homo sapiens GN=HLA-B PE=3 SV=1
F6IR35 **Mass:** 38417 **Score:** 48 **Queries matched:** 1
MHC class I antigen (Fragment) OS=Homo sapiens GN=HLA-A PE=3 SV=1
12. I0CMK4 **Mass:** 24530 **Score:** 44 **Queries matched:** 1 **emPAI:** 0.13
Beta-tubulin 4Q isoform 1 (Fragment) OS=Homo sapiens GN=TUBB4Q PE=3 SV=1
Query Observed Mr(expt) Mr(calc) ppm Miss Score Expect Rank Peptide
997 **540.9515 1619.8327 1619.8283 2.71 0 44 0.00061 1 R.LHFFMPGFAPLTSR.G**
Proteins matching the same set of peptides:
Q1KSF8 **Mass:** 23896 **Score:** 44 **Queries matched:** 1
XTP3TPA-transactivated protein 1 OS=Homo sapiens GN=XTP3TPATP1 PE=2 SV=1
B2R6L0 **Mass:** 50264 **Score:** 44 **Queries matched:** 1
cDNA, FLJ93005, highly similar to Homo sapiens tubulin, beta polypeptide (TUBB), mRNA OS=Homo sapiens PE=2 SV=1
B3KML9 **Mass:** 44916 **Score:** 44 **Queries matched:** 1
cDNA FLJ11352 fis, clone HEMBA1000020, highly similar to Tubulin beta-2C chain OS=Homo sapiens PE=2 SV=1
B3KS31 **Mass:** 42324 **Score:** 44 **Queries matched:** 1
Tubulin, beta 6, isoform CRA_a OS=Homo sapiens GN=TUBB6 PE=2 SV=1
Q3ZCM7 **Mass:** 50257 **Score:** 44 **Queries matched:** 1
Tubulin beta-8 chain OS=Homo sapiens GN=TUBB8 PE=1 SV=2
B4DE77 **Mass:** 46211 **Score:** 44 **Queries matched:** 1
cDNA FLJ55189, highly similar to Tubulin beta-4 chain OS=Homo sapiens PE=2 SV=1
B4DFH6 **Mass:** 27062 **Score:** 44 **Queries matched:** 1
cDNA FLJ52536, highly similar to Tubulin beta-4 chain OS=Homo sapiens PE=2 SV=1
B4DJ43 **Mass:** 40883 **Score:** 44 **Queries matched:** 1
cDNA FLJ53341, highly similar to Tubulin beta-4 chain OS=Homo sapiens PE=2 SV=1
B4DMU8 **Mass:** 36239 **Score:** 44 **Queries matched:** 1
cDNA FLJ53063, highly similar to Tubulin beta-7 chain OS=Homo sapiens PE=2 SV=1
B4DP54 **Mass:** 47014 **Score:** 44 **Queries matched:** 1
cDNA FLJ52712, highly similar to Tubulin beta-6 chain OS=Homo sapiens PE=2 SV=1
B4DQN9 **Mass:** 42096 **Score:** 44 **Queries matched:** 1
cDNA FLJ50617, highly similar to Tubulin beta-7 chain OS=Homo sapiens PE=2 SV=1
B4DXZ5 **Mass:** 30901 **Score:** 44 **Queries matched:** 1
cDNA FLJ52029, highly similar to Tubulin beta-7 chain OS=Homo sapiens PE=2 SV=1
B4DY90 **Mass:** 52414 **Score:** 44 **Queries matched:** 1
cDNA FLJ56903, highly similar to Tubulin beta-7 chain OS=Homo sapiens PE=2 SV=1
B4E052 **Mass:** 40000 **Score:** 44 **Queries matched:** 1
cDNA FLJ52378, highly similar to Tubulin beta-7 chain OS=Homo sapiens PE=2 SV=1
B4E386 **Mass:** 34289 **Score:** 44 **Queries matched:** 1
cDNA FLJ52847, highly similar to Tubulin beta-6 chain OS=Homo sapiens PE=2 SV=1
F5H0I4 **Mass:** 42233 **Score:** 44 **Queries matched:** 1
Tubulin beta-8 chain OS=Homo sapiens GN=TUBB8 PE=3 SV=1
Q5JP53 **Mass:** 48135 **Score:** 44 **Queries matched:** 1
Tubulin beta chain OS=Homo sapiens GN=TUBB PE=4 SV=1
Q5SQY0 **Mass:** 46033 **Score:** 44 **Queries matched:** 1
Tubulin beta-8 chain OS=Homo sapiens GN=TUBB8 PE=3 SV=1
Q5ST81 **Mass:** 42114 **Score:** 44 **Queries matched:** 1
Tubulin beta chain OS=Homo sapiens GN=TUBB PE=3 SV=1
Q5SU16 **Mass:** 50095 **Score:** 44 **Queries matched:** 1
Beta 5-tubulin OS=Homo sapiens GN=TUBB PE=2 SV=1
A6NKZ8 **Mass:** 42204 **Score:** 44 **Queries matched:** 1
Putative tubulin beta chain-like protein ENSP00000290377 OS=Homo sapiens PE=5 SV=2
Q6LC01 **Mass:** 49362 **Score:** 44 **Queries matched:** 1
MRNA encoding beta-tubulin. (from clone D-beta-1) (Fragment) OS=Homo sapiens PE=2 SV=1
B7Z4N1 **Mass:** 40211 **Score:** 44 **Queries matched:** 1
cDNA FLJ53906, highly similar to Tubulin beta chain OS=Homo sapiens PE=2 SV=1
B7ZAF0 **Mass:** 46936 **Score:** 44 **Queries matched:** 1
cDNA, FLJ79164, highly similar to Tubulin beta-7 chain OS=Homo sapiens PE=2 SV=1
B7ZAK1 **Mass:** 42054 **Score:** 44 **Queries matched:** 1
cDNA, FLJ79215, highly similar to Tubulin beta-7 chain OS=Homo sapiens PE=2 SV=1
K7ESM5 **Mass:** 37870 **Score:** 44 **Queries matched:** 1
Tubulin beta-6 chain (Fragment) OS=Homo sapiens GN=TUBB6 PE=2 SV=1
Q8IWP6 **Mass:** 50177 **Score:** 44 **Queries matched:** 1
Class IVb beta tubulin OS=Homo sapiens PE=2 SV=1
Q8IZ29 **Mass:** 50264 **Score:** 44 **Queries matched:** 1
Tubulin, beta 2C OS=Homo sapiens GN=TUBB2C PE=2 SV=1
Q8N6N5 **Mass:** 50232 **Score:** 44 **Queries matched:** 1
Tubulin, beta 2C OS=Homo sapiens GN=TUBB2C PE=2 SV=1
Q9BUF5 **Mass:** 50281 **Score:** 44 **Queries matched:** 1

Tubulin beta-6 chain OS=Homo sapiens GN=TUBB6 PE=1 SV=1
Q9BUU9 **Mass:** 38690 **Score:** 44 **Queries matched:** 1
TUBB protein (Fragment) OS=Homo sapiens GN=TUBB PE=2 SV=1
Q9BVA1 **Mass:** 50377 **Score:** 44 **Queries matched:** 1
Tubulin beta-2B chain OS=Homo sapiens GN=TUBB2B PE=1 SV=1
Q9UGA2 **Mass:** 37191 **Score:** 44 **Queries matched:** 1
Putative uncharacterized protein DKFZp566F223 (Fragment) OS=Homo sapiens GN=DKFZp566F223 PE=2 SV=1
Q96B85 **Mass:** 30625 **Score:** 44 **Queries matched:** 1
TUBB protein (Fragment) OS=Homo sapiens GN=TUBB PE=2 SV=1
Q96HX0 **Mass:** 26029 **Score:** 44 **Queries matched:** 1
TUBB2C protein (Fragment) OS=Homo sapiens GN=TUBB2C PE=2 SV=1
P04350 **Mass:** 50010 **Score:** 44 **Queries matched:** 1
Tubulin beta-4A chain OS=Homo sapiens GN=TUBB4A PE=1 SV=2
P07437 **Mass:** 50095 **Score:** 44 **Queries matched:** 1
Tubulin beta chain OS=Homo sapiens GN=TUBB PE=1 SV=2
Q13885 **Mass:** 50274 **Score:** 44 **Queries matched:** 1
Tubulin beta-2A chain OS=Homo sapiens GN=TUBB2A PE=1 SV=1
O43209 **Mass:** 38945 **Score:** 44 **Queries matched:** 1
Homo sapiens clone 23678 (Fragment) OS=Homo sapiens PE=2 SV=1
P68371 **Mass:** 50255 **Score:** 44 **Queries matched:** 1
Tubulin beta-4B chain OS=Homo sapiens GN=TUBB4B PE=1 SV=1
I3L2F9 **Mass:** 46054 **Score:** 42 **Queries matched:** 1
Uncharacterized protein OS=Homo sapiens PE=3 SV=1
A6NNZ2 **Mass:** 50168 **Score:** 42 **Queries matched:** 1
Tubulin beta-8 chain-like protein LOC260334 OS=Homo sapiens PE=1 SV=1
13. A0N4V7 **Mass:** 2269 **Score:** 42 **Queries matched:** 15 **emPAI:** 6.57
HCG2039797 (Fragment) OS=Homo sapiens GN=Tcr-alpha PE=4 SV=1
Query Observed Mr(expt) Mr(calc) ppm Miss Score Expect Rank Peptide
496 **421.7581 841.5016 841.5022 -0.63 1 42 0.0013 1 K.GITLSVRP.-** 495 497 498 499 500 501 502 503 504 505 506 507 508 509
14. L0R5A1 **Mass:** 11752 **Score:** 37 **Queries matched:** 3 **emPAI:** 0.28
Alternative protein CSF2RB OS=Homo sapiens GN=CSF2RB PE=4 SV=1
Query Observed Mr(expt) Mr(calc) ppm Miss Score Expect Rank Peptide
533 **428.7661 855.5176 855.5178 -0.28 0 37 0.003 1 R.LASIVTPR.N** 531 532
15. B7Z945 **Mass:** 20378 **Score:** 27 **Queries matched:** 3 **emPAI:** 0.16
Protein LOC100132891 OS=Homo sapiens GN=LOC100132891 PE=2 SV=1
Query Observed Mr(expt) Mr(calc) ppm Miss Score Expect Rank Peptide
895 **648.3401 1294.6656 1294.6629 2.06 1 27 0.024 1 R.VGAQVQNPRAPR.K** 893 894
16. I3L4V6 **Mass:** 26188 **Score:** 27 **Queries matched:** 1 **emPAI:** 0.12
Nucleoredoxin (Fragment) OS=Homo sapiens GN=NXN PE=4 SV=1
Query Observed Mr(expt) Mr(calc) ppm Miss Score Expect Rank Peptide
1083 **913.8988 1825.7831 1825.7854 -1.25 0 27 0.014 1 R.LYGIQDSEDDGESEAAK.Q**
Proteins matching the same set of peptides:
B4DNN6 **Mass:** 14224 **Score:** 27 **Queries matched:** 1
Nucleoredoxin OS=Homo sapiens GN=NXN PE=2 SV=1
17. Q59E90 **Mass:** 113918 **Score:** 25 **Queries matched:** 2 **emPAI:** 0.03
Mannosidase, alpha, class 2B, member 1 variant (Fragment) OS=Homo sapiens PE=2 SV=1
Query Observed Mr(expt) Mr(calc) ppm Miss Score Expect Rank Peptide
869 **613.8600 1225.7054 1225.7030 1.95 0 25 0.021 1 K.LIQLVNAQQAK.G** 868
